# Single cell transcriptomics profiling of the stromal cells in the pathologic association of ribosomal proteins in the ischemic myocardium and epicardial fat

**DOI:** 10.1007/s00441-024-03933-3

**Published:** 2024-12-06

**Authors:** An Thai, Christian Doescher, Nawfal Kamal, Darren Teramoto, Cameron Fung, Ed Cha, Vy La, Pauline Cheng, Sharona Sedighim, Angelo Keklikian, Finosh G. Thankam

**Affiliations:** Department of Translational Research, College of Osteopathic Medicine of the Pacific, Western University of Health Sciences, 309 E. Second Street, Pomona, CA 91766-1854 USA

**Keywords:** Epicardial adipose tissue, Hyperlipidemia, Left ventricular myocardium, Myocardial ischemia, Ribosomal proteins

## Abstract

Sustenance of ischemia in the surviving cardiac tissue following myocardial infarction (MI) elicits a proinflammatory milieu resulting in subsequent pathological episodes. Also, the activation and release of ribosomal proteins under ischemic insults have been unveiled; however, their extra ribosomal functions are unknown. We identified the ribosomal proteins including RPL10A, RPL14, RPL30, RPS18, FAU-40 (RPS30), and RPSA (Laminin Receptor, LR) in the vesicles of ischemia challenged epicardial adipose tissue derived stromal cells (EATDS). The present study aimed to assess the association of these proteins in the epicardial adipose tissues (EAT) and left ventricular (LV) myocardium and isolated stromal cells (EATDS and LVSCs) from hyperlipidemic (HL), MI and coronary artery bypass graft (CABG) swine models. The findings revealed an upregulation of RPL10A, RPL14, RPL30, RPS18, RPS30, and RPSA in the LV tissues of CABG and HL swine with a concomitant reduction in the MI group. RPS30 displayed similar upregulation in EAT, whereas the expression of other ribosomal proteins was not significantly altered. Additionally, the ischemic LVSCs and EATDS displayed altered expression status of these genes compared to the control. Also, the RPS18 + , RPL30 + and RPSA + LVSCs favored ischemia and revealed similar anti-inflammatory and regenerative sub-phenotypes reflecting the protective/survival mechanisms. Further understanding regarding the underlying molecular mechanisms and functions of these ribosomal proteins offers immense translational opportunities in the better management of ischemic cardiac complications.

**Supplementary Information:**

The online version contains supplementary material available at 10.1007/s00441-024-03933-3.

## Introduction

Sustenance of ischemia in the surviving cardiac tissue following myocardial infarction (MI) elicits a proinflammatory milieu resulting in subsequent pathological episodes (Thankam and Agrawal 2020). Even though coronary artery bypass graft (CABG) remains to be the primary treatment modality in MI, previous research shows evidence of increased pro-inflammatory pool within the vein graft and myocardium surrounding the anastomoses impeding the resolution of inflammation hurdling the functional recovery of the damaged myocardium (Thankam et al. 2022c). In addition, the intimate association/co-morbidity of hyperlipidemia (HL) further exacerbates the overall pathology in the ischemic myocardium (Pluijmert et al. 2019) (Zhang et al. 2022b). Moreover, the communication among multiple stromal cell populations including the epicardial adipose tissue derived stromal cells (EATDS) and left ventricular stromal cells (LVSC) plays a crucial role in the survival responses mainly through the secretory mediators (Thankam et al. 2022b) (Gui et al. 2021) (Fang et al. 2021).

Importantly, the EATDS-derived exosomes have been unveiled for their potential cardiac responses and our previous findings identified major ribosomal proteins including RPL10A, RPL14, RPL30, RPS18, FAU-40 (RPS30), and RPSA (Laminin Receptor, LR) in the vesicles of ischemia-challenged EATDS (Thankam et al. 2022a). Interestingly, the pathophysiological response of extracellular ribosomal proteins in promoting anti-inflammatory responses have been unveiled (Poddar et al. 2013); however, information regarding the regenerative function of extracellular ribosomal proteins are largely unknown. Apart from the structural and regulatory roles in the assembly of the ribosome, the ribosomal proteins have been reported to be involved in the regulation of cell growth, proliferation, differentiation, immune signaling, DNA repair and apoptosis which are directed by non-ribosomal cellular components (Kang et al. 2021). Moreover, the damaged cells release ribosomal proteins to the ECM which elicit potential biological effects. For instance, extracellular RPS19 inhibits the proinflammatory effect of macrophage migration inflammatory factor (MIF), a chemoattractant for monocytes, by preventing its interaction with chemokine receptors and CD74 (Gough 2009). However, the protective role of ribosomal proteins in ischemic cardiac pathology is largely unknown. On this background, the present descriptive study focuses on assessing the association of RPL10A, RPL14, RPL30, RPS18, RPS30 (FAU-40), and LR in the tissues and isolated stromal cells from epicardial adipose tissues (EAT) and left ventricular (LV) myocardium of hyperlipidemic, MI and CABG swine models. The study identifies the expression status of ribosomal proteins in driving unique phenotypes of stromal cell population for cardiac regeneration offering promise for novel translational for myocardial management.

## Methodology

### Animal models for MI and CABG

Animal protocol for establishing the minimally invasive swine-MI model was approved by the Institutional Animal Care and Use Committee (IACUC) of Western University of Health Sciences, Pomona, California, USA (Protocol# R21IACUC012) and for hyperlipidemia (HL) and CABG were approved by the IACUC of Creighton University, Omaha, Nebraska, USA (Protocol# 1052). Yucatan microswine (*Sus scorfa*, from Sinclair bioresources) were utilized for developing the HL by feeding the animals with 1-pound of high cholesterol high calorie feed twice daily for 6–12 months, and their weight gain was monitored. Blood analyses such as CBC (complete blood count), Complete Metabolic Panel (CMP), Lipid profile, CRP, coagulation profile and cardiac enzymes were performed as reported in our previous publication (Radwan et al. 2022). All the animals were provided with uninterrupted access to water throughout the study. In four HL pigs, CABG was performed at diagonal branch of left anterior descending (LAD) artery with left internal mammalian artery (LIMA) using the SEV (superficial epigastric vein) graft as detailed in our previous publications (Radwan et al. 2022) (Thankam et al. 2020). Acute MI was induced by angiography guided occlusion of coronary artery using a balloon catheter following our recent report (3) in Yucatan miniswine where the MI pigs were maintained on a normal diet. The HL pigs were sacrificed after 4–6 months following the feeding of high cholesterol high calorie diet, the CABG pigs were sacrificed 6–8 months following the procedure and the MI pigs within a week following MI. The ischemic left ventricular (LV) tissue at the distal anastomosis (LV-CABG group) and the infarct zone tissue from MI pigs (LV-MI group) were harvested for further studies. Similarly, the epicardial adipose tissue (EAT) was harvested from CABG and MI pigs forming the respective EAT-CABG and EAT-MI groups. The LV and EAT tissues harvested from similar anatomical locations of HL pigs and mini pigs without any interventions served as control (LV-C and LV-HL and EAT-C and EAT-HL, respectively).

### Tissue immunofluorescence

Expression level of the ribosomal protein mediators RPL10A, RPS30, RPL30, Laminin Receptor (LR), RPS18, and RPL14 and the cardiac biomarker Myosin Light Chain (MLC) in the LV and EAT were determined by immunofluorescence (Thankam et al. 2020). The LV and EAT of 5 µm thickness were used and antibodies against Myosin Light Chain (ab48003), FAU40S (ab234835), RPL-10A (ab187998), RPL-30 (ab170930), Laminin Receptor (ab137388), RPS-18 (ab224579) and RLP-14 (ab181200) at a dilution of 1:300 with corresponding fluorochrome secondary antibodies at 1:400 dilution were used for the staining. Nuclei were counterstained with 4′,6-diamidino-2-phenylindole (DAPI) (H-1200), imaged in a fluorescent slide scanner system (Leica, Thunder) at 20 × magnification, mean fluorescence intensity (MFI) was quantified using ImageJ software, MFI was normalized to the number of cells and the results were expressed as log_2_ fold-change (FC) normalized to normal control group as reported in our previous publication (Fang et al. 2022).

### Cell isolation, culture, and maintenance

The LV stromal cells (LVSC) and EATDS were isolated respectively from LV tissues and EAT, characterized, cultured, and grouped into control (no treatment), ischemic (ISC) (challenged with ischemia), or ischemic reperfusion (ISC/R) (challenged with ischemia for 2 h and reperfused with complete media) following our previously reported protocols (Thankam et al. 2022b) (Thankam and Agrawal 2021) (Thankam et al. 2023). LVSC and EATDS were maintained in DMEM under standard conditions prior to the treatments.

#### qRT-PCR

TRIzole method was used to isolate total cellular RNA from each group of LVSC and EATDS, cDNA was prepared using cDNA synthesis kit (Promega Madison, WI), mRNA transcripts for RPL10A, MLC, FAU40S, RPL10A, RPL30, LR, RPS18, and RLP-14 were amplified using specific set of primers (Table 1) and quantified by real-time PCR (Applied Biosystems, Waltham, MA) employing SYBR Green chemistry following our established protocols (Fang et al. 2022). GAPDH was used as a housekeeping reference gene and the results were represented as log_2_ FC with respect to the control.

**Table 1:** List of primers used for qRT-PCR analysis

Primers	Sequence
RPS30 F	TGCGCAAAGCGTAAGGAGG
RPS30 R	ACAAAGAGCTGCATGTTGGCG
RPS18 F	ATCGATGGGCGGCGGAAA
RPS18 R	TGGTGAGGTCGATGTCTGCT
RPL10A F	TGCGCGAGCTAGTCACTTT
RPL10A R	AGCGTTTGTCCTTCTGTGGGT
RPSA F	AAACTTCTGTTGGCGGCTCGT
RPSA R	GGCCAGCAATAGGAGTGGCT
RPL30 F	TGCGAAGAGCTTGGCATTGT
RPL30 R	TAACCAGCTGGAGCCGAGAG
RPL14 F	AGCCACAAGGTGGGCCAAGAA
RPL14 R	TCAGGAGAGCTGCCTTTTGAAGTT
GAPDH F	CAGACAGCCGTGTGTTCCGT
GAPDH R	CGATGCGGCCAAATCCGTT

#### Immunofluorescence

LVSC and EATDS were cultured in 8-well chamber slides and ISC and ISC/R were induced as mentioned above. The cells were formalin fixed for 1 h and immunostaining for RPL10A, MLC, FAU40S, RPL30, LR, RPS18 and RLP14 where the imaging and quantification were performed as mentioned above. The primary and secondary antibodies were used in a dilution of 1:300 and 1:500, respectively.

#### Western blot

The LVSC and EATDS were treated in T75 flasks, lysed using M-per lysis buffer (Cat# 78501, Thermo Fischer) and 100 µg sample was reduced in Laemmli buffer, denatured at 95 °C, separated by SDS-PAGE, transferred to PVDF membrane at 20 V for 25 min (TRANS BLOT, Bio-Rad), blocked with 5% nonfat dry milk in PBS-T (Bio-Rad, Cat #1706404) for 3 h, incubated with primary antibodies RPL10A, MLC, RPL30, LR, RPS18 and RLP14 (1:1000 in PBS-T) overnight at 4 °C, and imaged following the incubation with fluorochrome-conjugated secondary antibodies for 2 h. The band area was calculated using ImageJ software and normalized to GAPDH (ab8245), and Log_2_ fold-change based on control was calculated (Thankam et al. 2022b). FAU40S was exempted due to the unavailability of swine specific antibodies for WB.

### Single cell RNA sequencing (scRNA-seq)

scRNAseq was performed to assess the expression profile at single cell dimension to phenotype LVSC and EATDS based on the upregulation of RPL30, LR, and RPS18 in EATDS and LVSC under ISC and ISC/R compared to control. Single-cell library preparation on the LVSC and EATDS was performed using the Chromium Single Cell 5′ v2 chemistry at commercially available 10 × Genomics Chromium System (Children's Hospital Los Angeles SC2 Core, CA), considering ~ 10,000 cells per sample following our previous protocols (Thankam and Agrawal 2021) (Thankam et al. 2022b) (Thankam et al. 2023). The processed raw data was assessed using sus_scrofa_11 database (https://uswest.ensembl.org/Sus_scrofa/Info/Index) for scoring the gene expression and analyzed in Loupe Browser 5.0.1. using graph-based analysis mode. The cell clusters expressing the specific set of genes were analyzed in LibraryId mode and categorized based on the log_2_ fold-change to screen upregulated and downregulated genes (Thankam et al. 2023).

### Pathway analysis

The highly altered genes (FC > 2 and FC < 2) were assessed for the pathways and gene interactions based on the expression status in each cluster. The lists of genes given in the descending order of the FC in the cluster of interest (compared with other clusters) were converted to ‘.txt’ files and was analyzed in the program PATHVIEW (https://pathview.uncc.edu) following the instructions provided. SYMBOL was chosen for gene input and the analysis was based on human genome using KEGG database. The FC range for the analysis was set according to the FC of the highly altered genes. The pathways were created automatically by the program and were downloaded as png files (Luo and Brouwer 2013).

### Statistical analysis

The protein expression using immunofluorescence from LV and EAT was quantified using ImageJ and represented as average ± standard deviation of log2 FC and the statistical significance was determined by Multiple *‘t’* tests using GraphPad Prism software 8.2.1 (441). Also, the log_2_ FC for immunostaining, WB, and qRT-PCR in LVSC and EATDS was expressed as mean ± SEM. The statistical significance for all experiments was evaluated by one way ANOVA using Tukey’s multiple comparison test employing GraphPad Prism software 8.2.1 (441). The mean for each specimen/experimental replicate was the average MFI of at least 3 images randomly acquired images which was used for calculating log_2_ FC in the immunostaining. The samples with folds or background were exempted from analysis. *P* < 0.05 was set as statistically significant for all experiments.

## Results

### RPS18

**Fig. 1:**
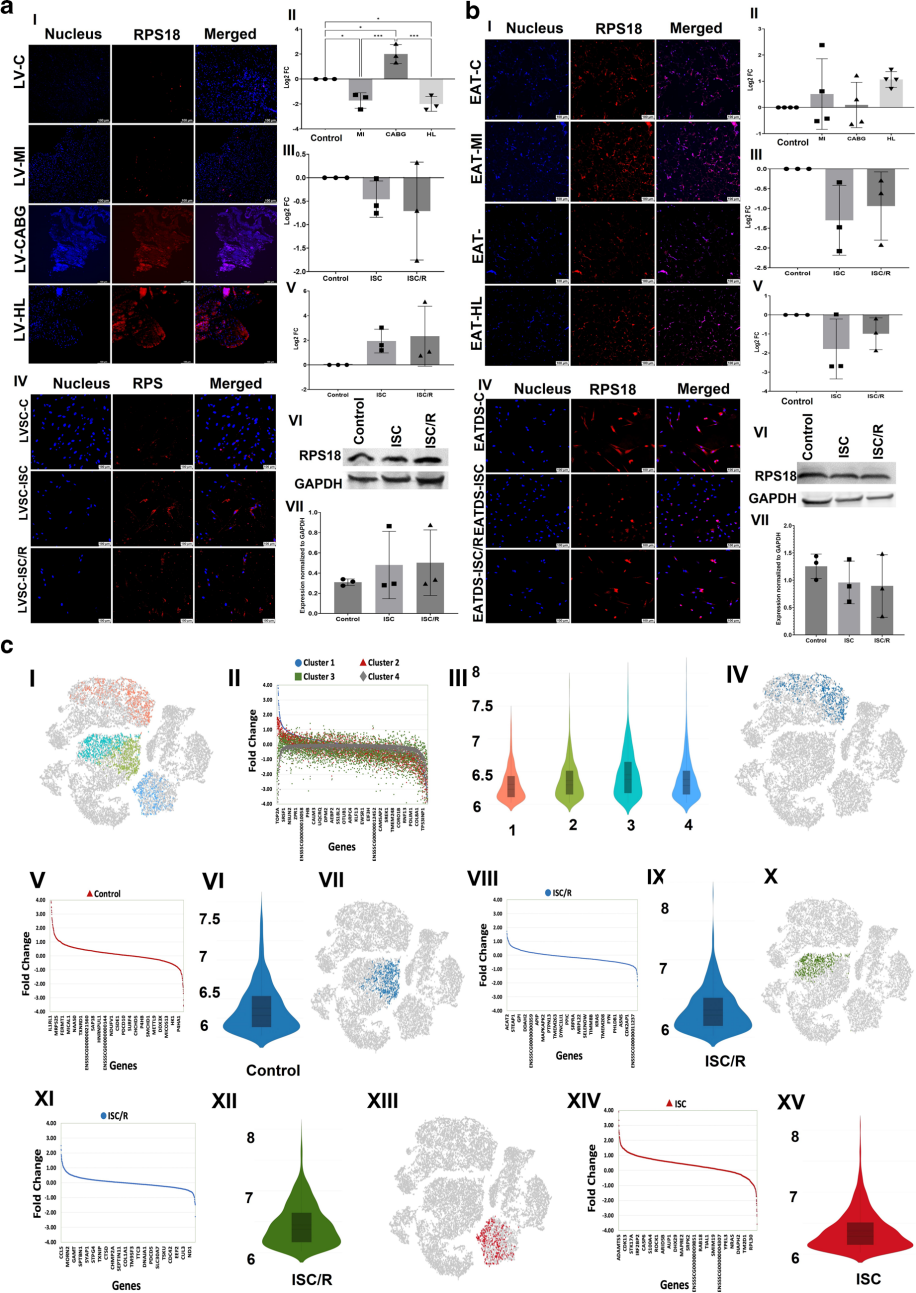
**a** Representative images for the immunofluorescence analysis for the expression of RPS18 in the LV tissues (**1a-I**) (*N* = 3) and the quantification of protein expression of RPS18 in LV tissues calculated from MFI (**1a-II**). (**1a-III**). Gene expression analysis of RPS18 in LVSC (*N* = 3) following ISC and ISC/R challenge using qRT-PCR relative to the housekeeping gene GAPDH normalized to the control and presented as Log_2_ FC. Representative images for the immunofluorescence analysis for the expression of RPS18 in the LVSC (**1a-IV**) (*N* = 3) following the treatment with ISC and ISC/R and (**1a.V**) the quantification of protein expression calculated from MFI. (**1a.VI**) Representative image of Western blot showing the expression status of RPS18 in the ISC and ISC/R groups compared to the control in LVSC (*N* = 3) and (**1a.VI**) quantification represented as fold change relative to GAPDH normalized to control. **b** Representative images for the immunofluorescence analysis for the expression of RPS18 in EAT (**1b-I**) (*N* = 4) and the quantification of protein expression of RPS18 in EAT tissues calculated from MFI (**1b-II**). (**1b-III**). Gene expression analysis of RPS18 in EATDS (*N* = 3) following ISC and ISC/R challenge using qRT-PCR relative to the housekeeping gene GAPDH normalized to the control and presented as Log_2_ FC. Representative images for the immunofluorescence analysis for the expression of RPS18 in the EATDS (**1b-IV**) (*N* = 3) following treatment with ISC and ISC/R and (**1b.V**) the quantification of protein expression calculated from MFI. (**1b.VI**) Representative image of Western blot showing the expression status of RPS18 in the ISC and ISC/R groups compared to the control in EATDS (*N* = 3) and (**1b.VII**) quantification represented as fold change relative to GAPDH normalized to control. **c** Single cell genomics analysis of RPS18 + LVSC: (**1c-I**) t-SNE plot showing the distribution of cells within all clusters based on the global expression of 3360 genes. (**1c-II**) Scatter plot showing the FC expression indicating the alteration of all genes in all clusters. (**1c-III**) Violin plot showing the similar distribution of the genes in all clusters between FC 6 and 8. (**1c-IV**) t-SNE plot of the Control group showing the distribution of cells within Cluster 1 based on the global expression of 2445 genes. (**1c-V**) Scatter plot showing the FC expression indicating the alteration of Cluster 1 genes in the Control group. (**1c-VI**) Violin plot showing the level of distribution of upregulated genes between FC 6 and 7.5 in the Control group. (**1c-VII**) t-SNE plot of ISC/R group showing the distribution of cells within Cluster 2 based on the global expression of 2118 genes. (**1c-VIII**) Scatter plot showing the FC expression indicating the alteration of Cluster 2 genes in ISC/R group. (**1c-IX**) Violin plot showing the level of distribution of upregulated genes between FC 6 and 8 in the ISC/R group. (**1c-X**) t-SNE plot of the ISC/R group showing the distribution of cells within Cluster 3 based on the global expression of 2342 genes. (**1c-XI**) Scatter plot showing the FC expression indicating the alteration of Cluster 3 genes in the ISC/R group. (**1c-XII**) Violin plot showing the level of distribution of upregulated genes between FC 6 and 8 in the ISC/R group. (**1c-XIII**) t-SNE plot of the ISC group showing the distribution of cells within Cluster 4 based on the global expression of 2966 genes. (**1c-XIV**) Scatter plot showing the FC expression indicating the alteration of Cluster 4 genes in the ISC group. (**1c-XV**) Violin plot showing the level of distribution of upregulated genes between FC 6 and 8 in the ISC group

The level of RPS18 was significantly decreased in the LV-MI (*P* = 0.0256) and LV-HL (*P* = 0.0118) groups and was significantly increased in the LV-CABG group (*P* = 0.0113) compared to the control. Also, the LV-CABG displayed significantly increased expression of RPS18 compared to LV-MI (*P* = 0.0002) and LV-HL (*P* = 0.0001) groups and the variation between LV-MI and LV-HL groups was statistically not significant (*P* = 0.9371) (Fig. 1a-I and a-II). The mRNA transcripts of RPS18 were decreased in the ISC (*P* = 0.6764) and ISC/R LVSC (*P* = 0.4189) compared to the control. The extent of decrease was more in the ISC/R (*P* = 0.8810) group; however, was statistically not significant compared to the ISC group (Fig. 1a-III). The level of RPS18 was increased in the ISC (*P* = 0.3278) and ISC/R (*P* = 0.2224) groups of cultured LVSC compared to the control; however, the alterations between ISC and ISC/R groups were statistically not significant (*P* = 0.9463) as evident from immunostaining (Fig. 1a-IV and aV). Western Blot analysis of RPS18 in LVSC displayed increased expression in ISC/R (*P* = 0.6729) and ISC (*P* = 0.7327) when compared to control and between ISC and ISC/R groups (*P* = 0.9939) (Fig. 1a-VI and aVII).

RPS18 was increased in EAT-MI (*P* = 0.8111), EAT-CABG (*P* = 0.9983) and EAT-HL (*P* = 0.2963) groups compared to the control; however, the alterations among the groups were statistically not significant (Fig. 1b-I and b-II). The mRNA transcripts of RPS18 were decreased in ISC (*P* = 0.1435) and ISC/R EATDS (*P* = 0.3109) groups compared to the control; however, the increase was statistically not significant in ISC/R group. Also, the extent of decrease of RPS18 transcripts in ISC was statistically not significant (*P* = 0.8153) compared to ISC/R group (Fig. 1b-III). Immunostaining revealed that RPS18 was decreased in both ISC (*P* = 0.1636) and ISC/R EATDS (*P* = 0.5039) compared to the control; however, this difference was statistically not significant. Also, the extent of expression was statistically not significant in the ISC (*P* = 0.6336) compared to ISC/R group (Fig. 1b-IV and b-V). Western Blot analysis of RPS18 in EATDS displayed decreased expression in ISC (*P* = 0.6813) and ISC/R (*P* = 0.5757) when compared to control; however, was statistically not significant. Also, the decrease in ISC/R was statistically not significant compared to ISC groups (*P* = 0.9809) (Fig. 1b-VI and b-VII).

Single cell genomics revealed that 3309 LVSC were positive for RSP18, 953 cells were in the control group, 519 cells in the ISC group, and 1837 cells in the ISC/R group. Overall, the RSP18 + LVSC favored the ISC/R group and were mapped in 4 cluster displaying 3360 genes (Fig. 1c-I, c-II, and c-III) (Supplementary File 1). Cluster 1 displayed 953 RSP18 + LVSC where 100% cells were displayed in the Control group (Fig. 1c-IV), and the violin plot and scatterplot revealed the distribution of 2445 genes in cluster 1 based on expression status in the Control group (Fig. 1c-V, and c-VI) (Supplementary File 1). IL1RL1 (Interleukin 1 receptor-like 1) (FC = 5.40, *P* < 0.0001), CCNB1 (FC = 5.30, *P* < 0.0001), and TOP2A (Type IIA topoisomerase) (FC = 5.25, *P* < 0.0001), were highly upregulated in the Control group of cluster 1 RSP18 + LVSC (Supplementary Table 1). On the other hand, IGFBP2 (FC = -3.59, *P* < 0.0001), MGP (FC = -3.53, *P* < 0.0001), and BNIP3 (FC = -2.68, *P* < 0.0001) were significantly downregulated in the Control group of cluster 1 (Supplementary Table 1). Cluster 2 RSP18 + LVSC displayed 935 which were mapped in the ISC/R group (Fig. 1c-VII) and the violin plot and scatterplot revealed the distribution of 2118 genes in cluster 2 RSP18 + LVSC based on expression status in the ISC/R group (Fig. 1c-VIII, and c-IX) (Supplementary File 1). Interestingly, none of the genes were significantly upregulated in cluster 2 RSP18 + LVSC. On the other hand, DES (FC = -2.25, *P* < 0.0001) and SFRP2 (FC = -2.24, *P* < 0.0001) were significantly downregulated in the ISC/R group of cluster 2 RSP18 + LVSC (Supplementary Table 1). Cluster 3 displayed 902 RSP18 + LVSC where 100% cells were displayed in the ISC/R group (Fig. 1c-X), and the violin plot and scatterplot revealed the distribution of 2342 genes in cluster 3 based on expression status in the ISC/R group (Fig. 1c-XI and c-XII) (Supplementary File 1). CCL5 (FC = 2.49, *P* = 0.002), IGFBP2 (FC = 5.30, *P* < 0.0001), and MGP (FC = 2.18, *P* < 0.0001), were highly upregulated in the ISC/R group of cluster 3 RSP18 + LVSC (Supplementary Table 1). On the other hand, AREG (FC = -2.28, *P* < 0.0001) was the only gene significantly downregulated in the ISCR group of cluster 3 RSP18 + LVSC (Supplementary Table 1). Cluster 4 RSP18 + LVSC displayed 519 cells where 100% cells were displayed in the ISC group (Fig. 1c-XIII), and the violin plot and scatterplot revealed the distribution of 2966 genes in cluster 4 based on expression status in the ISC group (Fig. 1c-XIV and c-XV) (Supplementary File 1). ADAMTS5 (FC = 5.18, *P* < 0.0001), IGFBP5 (FC = 4.93, *P* < 0.0001), and IGF1 (FC = 4.09, *P* < 0.0001), were highly upregulated in the ISC group of cluster 4 RSP18 + LVSC (Supplementary Table 1). On the other hand, RPL22L1 (FC = -3.56, *P* < 0.0001), ISG15 (FC = -3.01, *P* < 0.0001), and MGP (FC = -2.70, *P* < 0.0001) were significantly downregulated in the ISC group of cluster 4 RSP18 + LVSC (Supplementary Table 1).

### RPSA

The level of RPSA was significantly decreased in LV-MI (*P* = 0.0034) and LV-CABG (*P* = 0.6839) and increased in LV-HL (*P* = 0.4266) compared to the control; however, alterations in LV-CABG and LV-HL were statistically not significant. Also, the LV-MI displayed significantly decreased expression of RPSA compared to LV-CABG (*P* = 0.0241) and LV-HL (*P* = 0.0003) groups and the variation between LV-CABG and LV-HL groups was statistically not significant (*P* = 0.0794) (**2a-I and 2a-II**). The mRNA transcripts of RPSA were increased in the ISC (*P* = 0.9817); however, was statistically not significant and was significantly decreased in ISC/R LVSC (*P* = 0.0004) compared to the control. Also, ISC (*P* = 0.0003) group displayed significantly increased transcription of RPSA compared to the ISC/R group (**2a-III**). The level of RPSA was significantly increased in the ISC (*P* = 0.0078) and was significantly decreased in ISC/R (*P* = 0.0009) groups of cultured LVSC compared to the control; however, the alterations between ISC and ISC/R groups were statistically not significant (*P* = 0.1058) as evident from immunostaining (**2a-IV and 2a-V**). Western Blot analysis of RPSA in LVSC displayed similar level of expression in ISC (*P* = 0.9995) and ISC/R (*P* = 0.8030) when compared to control and between ISC and ISC/R groups (*P* = 0.7874) (**2a-VI and 2a-VII**).

RPSA was decreased in EAT-MI (*P* = 0.7045), EAT-CABG (*P* = 0.9595) and EAT-HL (*P* = 0.5520) groups compared to the control; however, were statistically not significant. Also, the LV- CABG displayed increased expression of RPSA compared to LV-MI (*P* = 0.9319) and LV-HL (*P* = 0.9933) groups and were statistically not significant (**2b-I and 2b-II**). The mRNA transcripts of RPSA were decreased in ISC (*P* = 0.1092) and ISC/R EATDS (*P* = 0.0618) groups compared to the control; however, was statistically not significant. Also, the extent of decrease of RPSA transcripts in ISC was statistically not significant (*P* = 0.8959) compared to ISC/R group (**2b-III**). Immunostaining revealed that RPSA was decreased in both ISC (*P* = 0.9873) and ISC/R EATDS (*P* = 0.3314) compared to the control; however, was statistically not significant. Also, the extent of expression was statistically not significant in the ISC (*P* = 0.3806) compared to ISC/R group (**2b-IV and 2b-V**). Western Blot analysis of RPSA in EATDS displayed similar level of expression in ISC (*P* = 0.9118) and ISC/R (*P* = 0.6533) when compared to control and between ISC and ISC/R groups (*P* = 0.4337) (**2b-VI and 2b-VII**).

**Fig. 2:**
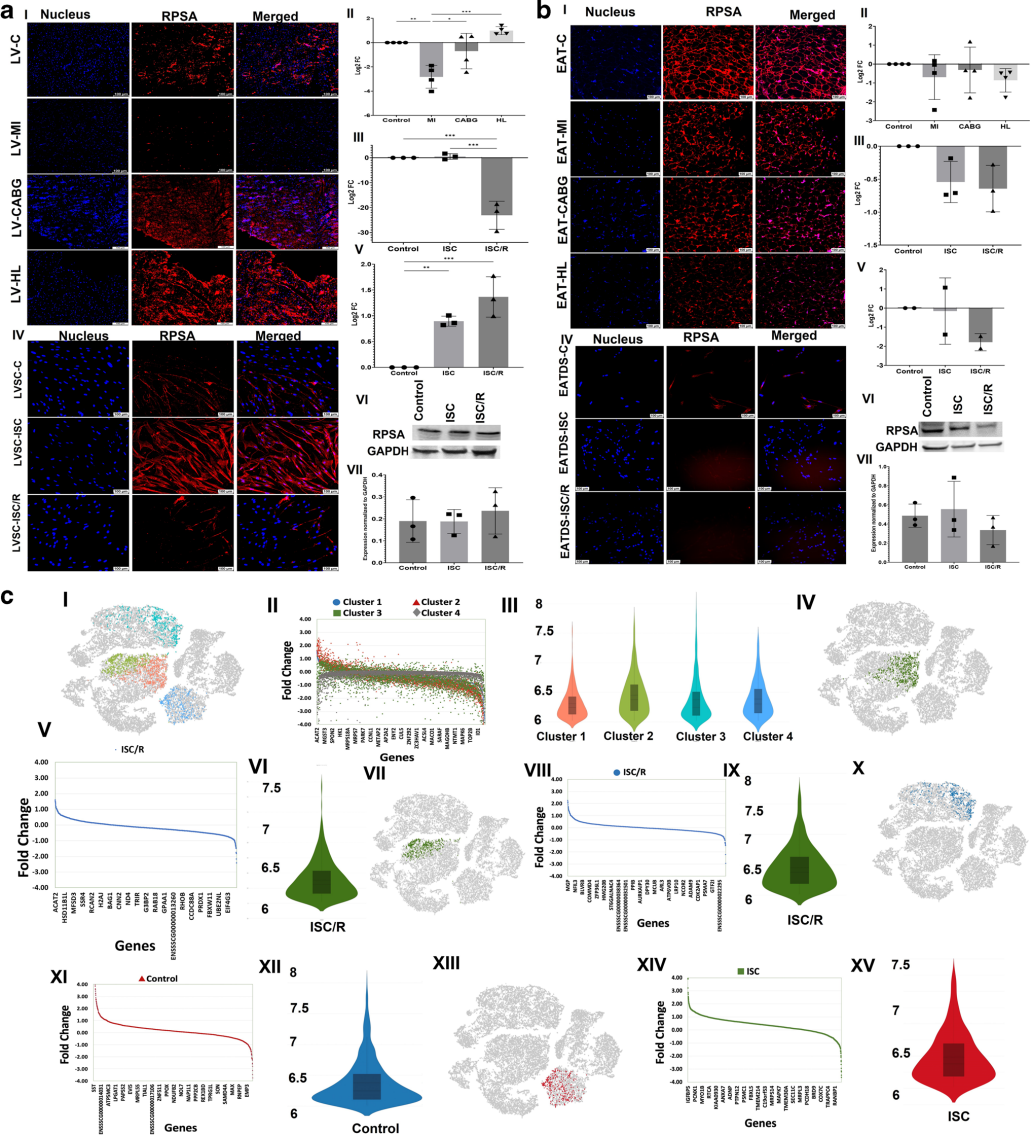
**a** Representative images for the immunofluorescence analysis for the expression of RPSA in the LV tissues (**2a-I**) (*N* = 4) and the quantification of protein expression of RPSA in LV tissues calculated from MFI (**2a-II**). (**2a-III**). Gene expression analysis of RPSA in LVSC (*N* = 3) following ISC and ISC/R challenge using qRT-PCR relative to the housekeeping gene GAPDH normalized to the control and presented as Log_2_ FC. Representative images for the immunofluorescence analysis for the expression of RPSA in the LVSC (**2a-IV**) (*N* = 3) following the treatment with ISC and ISC/R and (**2a.V**) the quantification of protein expression calculated from MFI. (**2a.VI**) Representative image of Western blot showing the expression status of RPSA in the ISC and ISC/R groups compared to the control in LVSC (*N* = 3) and (**2a.VI**) quantification represented as fold change relative to GAPDH normalized to control. **b** Representative images for the immunofluorescence analysis for the expression of RPSA in the EAT (**2b-I**) (*N* = 4) and the quantification of protein expression of RPSA in EAT tissues calculated from MFI (**2b-II**). (**2b-III**). Gene expression analysis of RPSA in EATDS (*N* = 3) following ISC and ISC/R challenge using qRT-PCR relative to the housekeeping gene GAPDH normalized to the control and presented as Log_2_ FC. Representative images for the immunofluorescence analysis for the expression of RPSA in the EATDS (**2b-IV**) (*N* = 3) following the treatment with ISC and ISC/R and (**2b.V**) the quantification of protein expression calculated from MFI. (**2b.VI**) Representative image of Western blot showing the expression status of RPSA in the ISC and ISC/R groups compared to the control in EATDS (*N* = 3) and (**2b.VII**) quantification represented as fold change relative to GAPDH normalized to control. **c** Single cell genomics analysis of RPSA + LVSC: (**2c-I**) t-SNE plot showing the distribution of cells within all clusters based on the global expression of 3624 genes. (**2c-II**) Scatter plot showing the FC expression indicating the alteration of all genes in all clusters. (**2c-III**) Violin plot showing the similar distribution of the genes in all clusters between FC 6 and 8. (**2c-IV**) t-SNE plot of ISC/R group showing the distribution of cells within Cluster 1 based on the global expression of 2320 genes. (**2c-V**) Scatter plot showing the FC expression indicating the alteration of Cluster 1 genes in ISC/R group. (**2c-VI**) Violin plot showing the level of distribution of upregulated genes between FC 6 and 7.5 in the ISC/R group. (**2c-VII**) t-SNE plot of ISC/R group showing the distribution of cells within Cluster 2 based on the global expression of 2558 genes. (**2c-VIII**) Scatter plot showing the FC expression indicating the alteration of Cluster 2 genes in ISC/R group. (**2c-IX**) Violin plot showing the level of distribution of upregulated genes between FC 6 and 8 in the ISC/R group. (**2cX**) t-SNE plot of the Control group showing the distribution of cells within Cluster 3 based on the global expression of 2664 genes. (**2c-XI**) Scatter plot showing the FC expression indicating the alteration of Cluster 3 genes in the Control group. (**2c-XII**) Violin plot showing the level of distribution of upregulated genes between FC 6 and 8 in the Control group. (**2c-XIII**) t-SNE plot of the ISC group showing the distribution of cells within Cluster 4 based on the global expression of 3188 genes. (**2c-XIV**) Scatter plot showing the FC expression indicating the alteration of Cluster 4 genes in the ISC group. (**2c-XV**) Violin plot showing the level of distribution of upregulated genes between FC 6 and 7.5 in the ISC group

Single cell genomics revealed that 2397 LVSC were positive for RPSA, 615 cells were in the control group, 358 cells in the ISC group, and 1424 cells in the ISC/R group. Overall, the RPSA + LVSC favored the ISC/R group and were mapped in 4 cluster based on the expression of 3624 genes (Fig. 2c-I, c-II, and c-III). Cluster 1 RPSA + LVSC displayed 713 cells where 100% cells were displayed in the ISC/R group (Fig. 2c-IV), and the violin plot and scatterplot revealed the distribution of 2320 genes in cluster 1 RPSA + LVSC based on expression status in ISC/R group (Fig. 2c-V and c-VI) (Supplementary File 1). DES (Desmin) (FC = -2.43, *P* < 0.0001) and SFRP2 (Secreted Frizzled Related Protein 2) (FC = -2.17, *P* < 0.0001) were the major genes significantly downregulated in the ISC/R group of cluster 1 (Supplementary Table 1). Cluster 2 RPSA + LVSC displayed 711 cells where all the cells were mapped in the ISC/R group (Fig. 2c-VII) and the violin plot and scatterplot revealed the distribution of 2558 genes in cluster 2 RPSA + LVSC based on expression status in the ISC/R group (Fig. 2c-VIII and c-IX) (Supplementary File 1). MGP (Matrix Gla protein) (FC = 2.26, *P* < 0.0001), CCL5 (C–C Motif Chemokine Ligand 5) (FC = 2.15, *P* = 0.0781), and IGFBP2 (Insulin Like Growth Factor Binding Protein 2) (FC = 2.08, *P* < 0.0001) were highly upregulated; however, the upregulation of CCL5 was statistically not significant (Supplementary Table 1). On the other hand, SFRP2 (FC = -2.95, *P* < 0.0001) and AREG (Amphiregulin) (FC = -2.22, *P* < 0.0001) were significantly downregulated in the ISC/R group of cluster 2 RPSA + LVSC (Supplementary Table 1). Cluster 3 RPSA + LVSC displayed 615 cells where all the cells were mapped in the Control group (Fig. 2c-X) and the violin plot and scatterplot revealed the distribution of 2664 genes in cluster 3 based on expression status in the Control group (Fig. 2c-XI and cXII) (Supplementary File 1). SST (Somatostatin) (FC = 9.44, *P* < 0.0001), CCNB1 (Cyclin B1) (FC = 5.43, *P* < 0.0001), and SHCBP1 (SHC Binding And Spindle Associated 1) (FC = 5.42, *P* < 0.0001) were highly upregulated (Supplementary Table). On the other hand, IGFBP2 (FC = -3.74, *P* < 0.0001), MGP (Amphiregulin) (FC = -3.47, *P* < 0.0001), and BNIP3 (BCL2 Interacting Protein 3) (FC = -2.78, *P* < 0.0001) were significantly downregulated in the Control group of cluster 3 (Supplementary Table 1). Cluster 4 RPSA + LVSC displayed 358 cells where all the cells were mapped in the ISC group (Fig. 2c-XIII) and the violin plot and scatterplot revealed the distribution of 3188 genes in cluster 4 RPSA + LVSC based on expression status in the ISC group (Fig. 2c-XIV and c-XV) (Supplementary File 1). IGFBP5 (insulin like growth factor binding protein 5) (FC = 5.25, *P* < 0.0001), ADAMTS5 (disintegrin and metalloproteinase with thrombospondin motifs) (FC = 5.10, *P* < 0.0001), and IGF1 (insulin-like growth factor 1) (FC = 4.01, *P* < 0.0001) were highly upregulated (Supplementary Table 1). On the other hand, RPL22L1 (Ribosomal Protein L22 Like 1) (FC = -3.63, *P* < 0.0001), ISG15 (Interferon-stimulated gene 15) (FC = -3.47, *P* < 0.0001), and MGP (FC = -3.21, *P* < 0.0001) were significantly downregulated in the Control group of cluster 3 RPSA + LVSC (Supplementary Table 1).

### RPL30

**Fig. 3:**
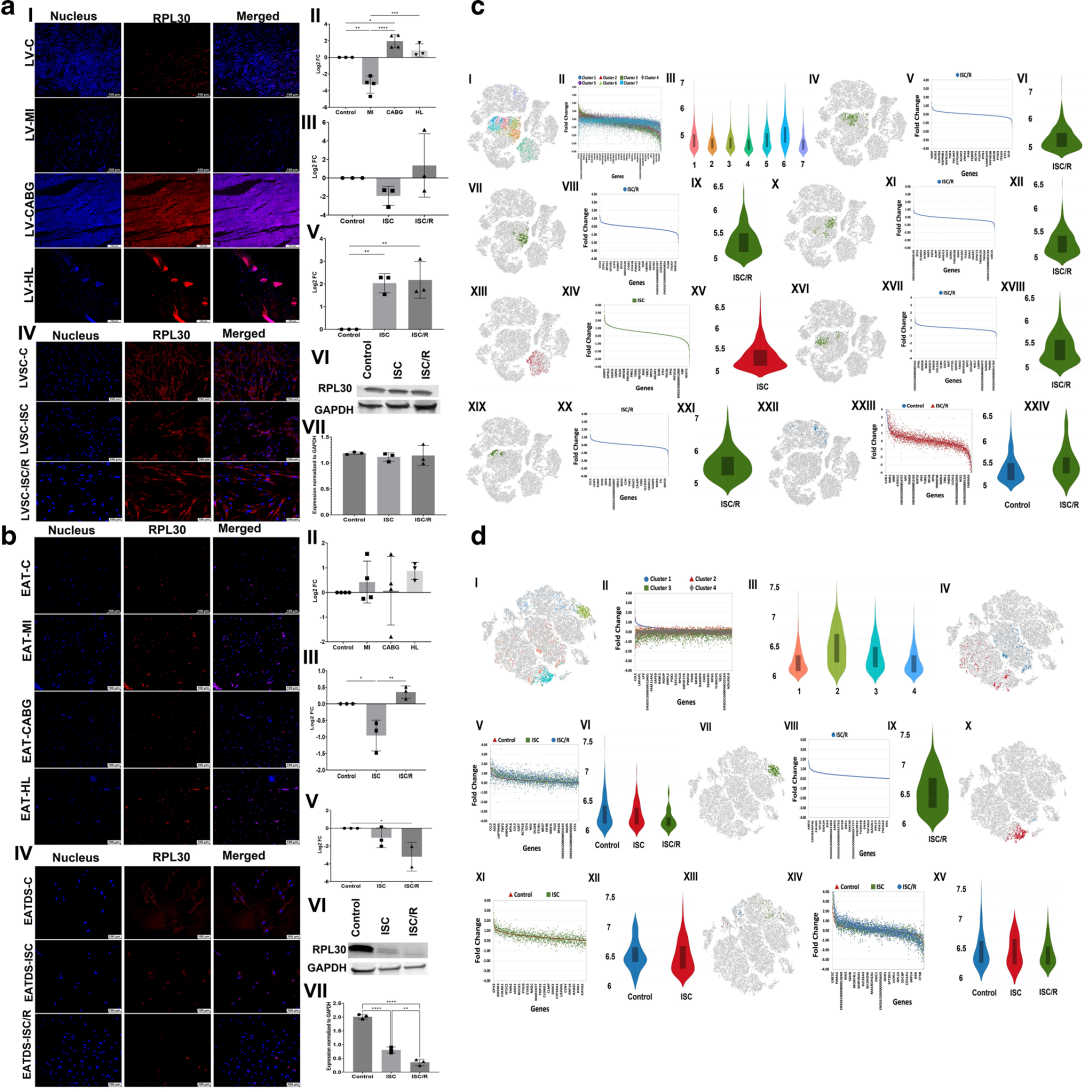
**a** Representative images for the immunofluorescence analysis for the expression of RPL30 in the LV tissues (**3a-I**) (*N* = 3) and the quantification of protein expression of RPL30 in LV tissues calculated from MFI (**3a-II**). (**3a-III**). Gene expression analysis of RPL30 in LVSC (*N* = 3) following ISC and ISC/R challenge using qRT-PCR relative to the housekeeping gene GAPDH normalized to the control and presented as Log_2_ FC. Representative images for the immunofluorescence analysis for the expression of RPL30 in the LVSC (**3a-IV**) (*N* = 3) following the treatment with ISC and ISC/R and (**3a.V**) the quantification of protein expression calculated from MFI. (**3a.VI**) Representative image of Western blot showing the expression status of RPL30 in the ISC and ISC/R groups compared to the control in LVSC (*N* = 3) and (**3a.VI**) quantification represented as fold change relative to GAPDH normalized to control. **b** Representative images for the immunofluorescence analysis for the expression of RPL30 in the EAT (**3b-I**) (*N* = 4) and the quantification of protein expression of RPL30 in EAT tissues calculated from MFI (**3b-II**). (**3b-III**). Gene expression analysis of RPL30 in EATDS (*N* = 3) following ISC and ISC/R challenge using qRT-PCR relative to the housekeeping gene GAPDH normalized to the control and presented as Log_2_ FC. Representative images for the immunofluorescence analysis for the expression of RPL30 in the EATDS (**3b-IV**) (*N* = 3) following the treatment with ISC and ISC/R and (**3b.V**) the quantification of protein expression calculated from MFI. (**3b.VI**) Representative image of Western blot showing the expression status of RPL30 in the ISC and ISC/R groups compared to the control in EATDS (*N* = 3) and (**3b.VII**) quantification represented as fold change relative to GAPDH normalized to control. **c** Single cell genomics analysis of RPL30 + LVSC: (**3c-I**) t-SNE plot showing the distribution of cells within all clusters based on the global expression of 3526 genes. (**3c-II**) Scatter plot showing the FC expression indicating the alteration of all genes in all clusters. (**3c-III**) Violin plot showing the similar distribution of the genes in all clusters between FC 4 and 6. (**3c-IV**) t-SNE plot of the Control group showing the distribution of cells within Cluster 1 based on the global expression of 2373 genes. (**3c-V**) Scatter plot showing the FC expression indicating the alteration of Cluster 1 genes in the ISC/R group. (**3c-VI**) Violin plot showing the level of distribution of upregulated genes between FC 6 and 7.5 in the ISC/R group. (**3c-VII**) t-SNE plot of ISC/R group showing the distribution of cells within Cluster 2 based on the global expression of 2146 genes. (**3c-VIII**) Scatter plot showing the FC expression indicating the alteration of Cluster 2 genes in ISC/R group. (**3c-IX**) Violin plot showing the level of distribution of upregulated genes between FC 5 and 6 in the ISC/R group. (**3c-X**) t-SNE plot of the ISC/R group showing the distribution of cells within Cluster 3 based on the global expression of 2267 genes. (**3c-XI**) Scatter plot showing the FC expression indicating the alteration of Cluster 3 genes in the ISC/R group. (**3c-XII**) Violin plot showing the level of distribution of upregulated genes between FC 5 and 6 in the ISC/R group. (3**c-XIII**) t-SNE plot of the ISC group showing the distribution of cells within Cluster 4 based on the global expression of 3084 genes. (**3c-XIV**) Scatter plot showing the FC expression indicating the alteration of Cluster 4 genes in the ISC group. (**3c-XV**) Violin plot showing the level of distribution of upregulated genes between FC 5 and 6 in the ISC group. (**3c-XVI**) t-SNE plot of the ISC/R group showing the distribution of cells within Cluster 5 based on the global expression of 2466 genes. (**3c-XVII**) Scatter plot showing the FC expression indicating the alteration of Cluster 5 genes in the ISC/R group. (**3c-XVIII**) Violin plot showing the level of distribution of upregulated genes between FC 5 and 6 in the ISC/R group. (**3c-XIX**) t-SNE plot of the ISC/R group showing the distribution of cells within Cluster 6 based on the global expression of 2453 genes. (**3c-XX**) Scatter plot showing the FC expression indicating the alteration of Cluster 6 genes in the ISC/R group. (**3c-XXI**) Violin plot showing the level of distribution of upregulated genes between FC 5 and 6 in the ISC/R group. (**3c-XXII**) t-SNE plot of the Control and ISC/R groups showing the distribution of cells within Cluster 7 based on the global expression of 2979 genes. (**3cXXIII**) Scatter plot showing the FC expression indicating the alteration of Cluster 7 genes in the Control and ISC/R group. (**3c-XXIV**) Violin plot showing the level of distribution of upregulated genes between FC 5 and 6 in the Control and ISC/R group. **d** Single cell genomics analysis of RPL30 + EATDS: (**3d-I**) t-SNE plot showing the distribution of cells within all clusters based on the global expression of 1960 genes. (**3d-II**) Scatter plot showing the FC expression indicating the alteration of all genes in all clusters. (**3d-III**) Violin plot showing the similar distribution of the genes in all clusters between FC 5 and 7. (**3d-IV**) t-SNE plot of the Control, ISC and ISC/R groups showing the distribution of cells within Cluster 1 based on the global expression of 1802 genes. (**3d-V**) Scatter plot showing the FC expression indicating the alteration of Cluster 1 genes in the Control, ISC and ISC/R group. (**3d-VI**) Violin plot showing the level of distribution of upregulated genes between FC 6 and 7.5 in the Control, ISC and ISC/R group. (**3d-VII**) t-SNE plot of ISC/R group showing the distribution of cells within Cluster 2 based on the global expression of 1761 genes. (**3d-VIII**) Scatter plot showing the FC expression indicating the alteration of Cluster 2 genes in ISC/R group. (**3c-IX**) Violin plot showing the level of distribution of upregulated genes between FC 5 and 7 in the ISC/R group. (**3c-X**) t-SNE plot of the Control and ISC groups showing the distribution of cells within Cluster 3 based on the global expression of 1516 genes. (**3d-XI**) Scatter plot showing the FC expression indicating the alteration of Cluster 3 genes in the Control and ISC groups. (**3d-XII**) Violin plot showing the level of distribution of upregulated genes between FC 5 and 6 in the Control and ISC groups. (3**d-XIII**) t-SNE plot of the Control, ISC and ISC/R groups showing the distribution of cells within Cluster 4 based on the global expression of 2153 genes. (**3d-XIV**) Scatter plot showing the FC expression indicating the alteration of Cluster 4 genes in the Control, ISC and ISC/R groups. (**3c-XV**) Violin plot showing the level of distribution of upregulated genes between FC 5 and 6 in the Control, ISC and ISC/R groups

The level of RPL30 was significantly decreased in LV-MI (*P* = 0.0015) and was significantly increased in LV-CABG (*P* = 0.0406) and the increase in LV-HL (*P* = 0.5884) was statistically not significant compared to the control. Also, the LV-MI displayed significantly decreased expression of RPL30 compared to LV-CABG (*P* < 0.0001) and LV-HL (*P* = 0.0003) groups and the variation between LV-CABG and LV-HL groups was statistically not significant (*P* = 0.3237) (Fig. 3a-I and a-II). The mRNA transcripts of RPL30 were decreased in the ISC (*P* = 0.5211) and ISC/R LVSC (*P* = 0.7148) compared to the control. The extent of decrease was more in the ISC (*P* = 0.2044) group; however, was statistically not significant compared to the ISC/R group (Fig. 3a-III). The level of RPL30 was significantly increased in the ISC (*P* = 0.0076) and ISC/R (*P* = 0.0055) groups of cultured LVSC compared to the control; however, the alterations between ISC and ISC/R groups were statistically not significant (*P* = 0.9421) as evident from immunostaining (Fig. 3a-IV and a-V). Western Blot analysis of RPL30 in LVSC displayed similar level of expression in ISC (*P* = 0.7568) and ISC/R (*P* = 0.9004) when compared to control and between ISC and ISC/R groups (*P* = 0.9556) (Fig. 3a-VI and a-VII).

RPL30 was increased in EAT-MI (*P* = 0.8975), EAT-CABG (*P* = 0.9995) and EAT-HL (*P* = 0.5641) groups compared to the control; however, the alterations among the groups were statistically not significant (Fig. 3b-I and b-II). The mRNA transcripts of RPL30 were significantly decreased in ISC (*P* = 0.0156) and increased in ISC/R EATDS (*P* = 0.3542) groups compared to the control; however, the increase was statistically not significant in ISC/R group. Also, the extent of decrease of RPL30 transcripts in ISC was statistically significant (*P* = 0.0034) compared to ISC/R group (Fig. 3b-III). Immunostaining revealed that RPL30 was decreased in both ISC (*P* = 0.4567) and ISC/R EATDS (*P* = 0.0398) compared to the control; however, was statistically not significant in the ISC group. Also, the extent of expression was statistically not significant in the ISC compared to ISC/R group (*P* = 0.1440) (Fig. 3b-IV and b-V). Western Blot analysis of RPL30 in EATDS displayed significantly decreased expression in ISC (*P* < 0.0001) and ISC/R (*P* < 0.0001) when compared to control. The decrease in ISC/R was statistically significant compared to ISC groups (*P* = 0.0041) (Fig. 3b-VI and b-VII).

Single cell genomics revealed that 2794 LVSC cells were positive for RPL30, 201 cells were in the control group, 375 cells in the ISC group, and 2218 cells in the ISC/R group. Overall, the RPL30 cells favored the ISC/R group and were mapped in 7 cluster based on 3526 genes (Fig. 3c-I, c-II, and c-III) (Supplementary File 1). Cluster 1 displayed 585 cells where 100% cells were displayed in the ISC/R group (Fig. 3c-IV), and the violin plot and scatterplot revealed the distribution of 2373 genes in cluster 1 RPL30 + LVSC based on expression status in ISC/R group (Fig. 3c-V and c-VI) (Supplementary Table 1). However, none of the genes was significantly upregulated. SFRP2 (FC = -2.18, *P* < 0.0001) was the only major gene significantly downregulated in the cluster 1 (Supplementary Table 1). Cluster 2 LVSC displayed 536 cells where all the cells were mapped in the ISC/R group (Fig. 3c-VII) and the violin plot and scatterplot revealed the distribution of 2146 genes in cluster 2 based on expression status in the ISC/R group (Fig. 3c-VIII and c-IX) (Supplementary File 1). Interestingly, none of the genes was significantly upregulated or downregulated. Cluster 3 LVSC displayed 477 cells where all the cells were mapped in the ISC/R group (Fig. 3c-X) and the violin plot and scatterplot revealed the distribution of 2267 genes in cluster 3 RPL30 + LVSC based on expression status in the ISC/R group (Fig. 3c-XI and c-XII) (Supplementary File 1). Interestingly, none of the genes was significantly upregulated or downregulated. Cluster 4 LVSC displayed 375 cells where all the cells were mapped in the ISC group (Fig. 3c-XIII) and the violin plot and scatterplot revealed the distribution of 2085 genes in cluster 4 LVSC based on expression status in the ISC group (Fig. 3c-XIV and c-XV) (Supplementary File 1). IGFBP5 (insulin like growth factor binding protein 5) (FC = 5.56, *P* < 0.0001), ADAMTS5 (disintegrin and metalloproteinase with thrombospondin motifs) (FC = 4.50, *P* < 0.0001), and ARL4D (DP Ribosylation Factor Like GTPase 4D) (FC = 4.23, *P* < 0.0001) were highly upregulated (Supplementary Table 1). On the other hand, ISG15 (FC = -3.59, *P* < 0.0001), RPL22L1 (FC = -3.58, *P* < 0.0001), and ISG12(A) (FC = -3.02, *P* < 0.0001) were significantly downregulated in the ISC group of cluster 4 RPL30 + LVSC (Supplementary Table 1). Cluster 5 RPL30 + LVSC displayed 336 cells where all the cells were mapped in the ISC/R group (Fig. 3c-XVI) and the violin plot and scatterplot revealed the distribution of 2466 genes in based on expression status in the ISC/R group (Fig. 3c-XVII and c-XVIII) (Supplementary File 1). None of the genes was significantly upregulated. SFRP2 (FC = -2.08, *P* = 0.003) was the only major gene significantly downregulated in the ISC/R group of cluster 5 RPL30 + LVSC (Supplementary Table 1). Cluster 6 RPL30 + LVSC displayed 268 cells where all the cells were mapped in the ISC/R group (Fig. 3c-XIX) and the violin plot and scatterplot revealed the distribution of 2453 genes based on expression status in the ISC/R group (Fig. 3c-XX and c-XXI) (Supplementary File 1). Interestingly, none of the genes was significantly upregulated whereas SFRP2 (FC = -2.22, *P* = 0.001) was the only major gene significantly downregulated in the ISC/R group of cluster 6 RPL30 + LVSC (Supplementary Table 1). Cluster 7 RPL30 + LVSC displayed 217 cells where 93% of the cells were mapped in the Control group and 7% of the cells were mapped in ISC/R (Fig. 3c-XXII) and the violin plot and scatterplot revealed the distribution of 2979 genes based on expression status in the Control group (Fig. 3c-XXIII and c-XXIV) (Supplementary File 1). IL1RL1 (Interleukin 1 receptor-like 1) (FC = 5.69, *P* < 0.0001), CCNB1 (FC = 5.34, *P* < 0.0001), and CDCA3 (Cell Division Cycle Associated 3) (FC = 5.34, *P* < 0.0001), were highly upregulated in the Control group of cluster 7 RPL30 + LVSC (Supplementary Table 1). On the other hand, HPS5 (Hermansky-Pudlak syndrome-5) (FC = -6.64, *P* < 0.0001), IFIT1 (Interferon Induced Protein With Tetratricopeptide Repeats 1) (FC = -6.35, *P* < 0.0001), and ISG15 (FC = -5.94, *P* < 0.0001) were significantly downregulated in the Control group of cluster 7 RPL30 + LVSC (Supplementary Table 1).

Single cell genomics revealed that 1572 EATDS cells were positive for RPL30, 309 cells were in the control group, 700 cells in the ISC group, and 563 cells in the ISC/R group. Overall, the RPL30 cells favored the ISC group and were mapped in 4 clusters based on the expression status of 1960 genes (Fig. 3d-I, d-II, and d-III) (Supplementary File 1). Cluster 1 displayed 561 cells where 52% cells were displayed in the Control group, 45% in the ISC group, and 4% in the ISC/R group (Fig. 3d-IV), and the violin plot and scatterplot revealed the distribution of 1802 genes in cluster 1 RPL30 + EATDS based on expression status in Control group (Fig. 3d-V and d-VI) (Supplementary Table 1). CCL5 (C–C motif chemokine ligand 5) (FC = 3.28, *P* = 0.45), IFIT1 (Interferon Induced Protein With Tetratricopeptide Repeats 1) (FC = 2.64, *P* < 0.0001), and HPS5 (Hermansky-Pudlak syndrome-5) (FC = 2.46, *P* = 0.20) were the major genes upregulated in the Control group of cluster 1 RPL30 + EATDS; however, the upregulation of CCL5 and HPS5 were not statistically significant (Supplementary Table 1). Cluster 2 RPL30 + EATDS displayed 543 cells where all the cells were mapped in the ISC/R group (Fig. 3d-VII) and the violin plot and scatterplot revealed the distribution of 1761 genes based on expression status in the ISC/R group (Fig. 3d-VIII and d-IX) (Supplementary File 1). Interestingly, none of the genes was significantly upregulated or downregulated suggesting basal expression and normal phenotypes. Cluster 3 RPL30 + EATDS displayed 468 cells where all the cells were mapped in the ISC group (Fig. 3d-X) and the violin plot and scatterplot revealed the distribution of 1516 genes in cluster 3 based on expression status in the ISC group (Fig. 3d-XI and d-XII) (Supplementary File 1). None of the genes was significantly upregulated or downregulated suggesting basal expression and normal phenotypes. Cluster 4 RPL30 + EATDS displayed 238 cells where 36% the cells were mapped in the Control group, 7% were mapped in the ISC group, and 58% were mapped in the ISC/R group (Fig. 3d-XIII). The violin plot and scatterplot revealed the distribution of 2153 genes based on expression status in the ISC/R group (Fig. 3d-XIV and d-XV) (Supplementary File 1). DHRS3 (Dehydrogenase/Reductase 3) (FC = -2.88, *P* = 1.000) and ALDH1A1 (Aldehyde Dehydrogenase 1 Family Member A1) (FC = -2.43, *P* = 1.000) were the major genes upregulated in the ISC/R group of cluster 4 RPL30 + EATDS; however, they were statistically insignificant. Notably, none of the genes was significantly upregulated.

### RPL10A

**Fig. 4:**
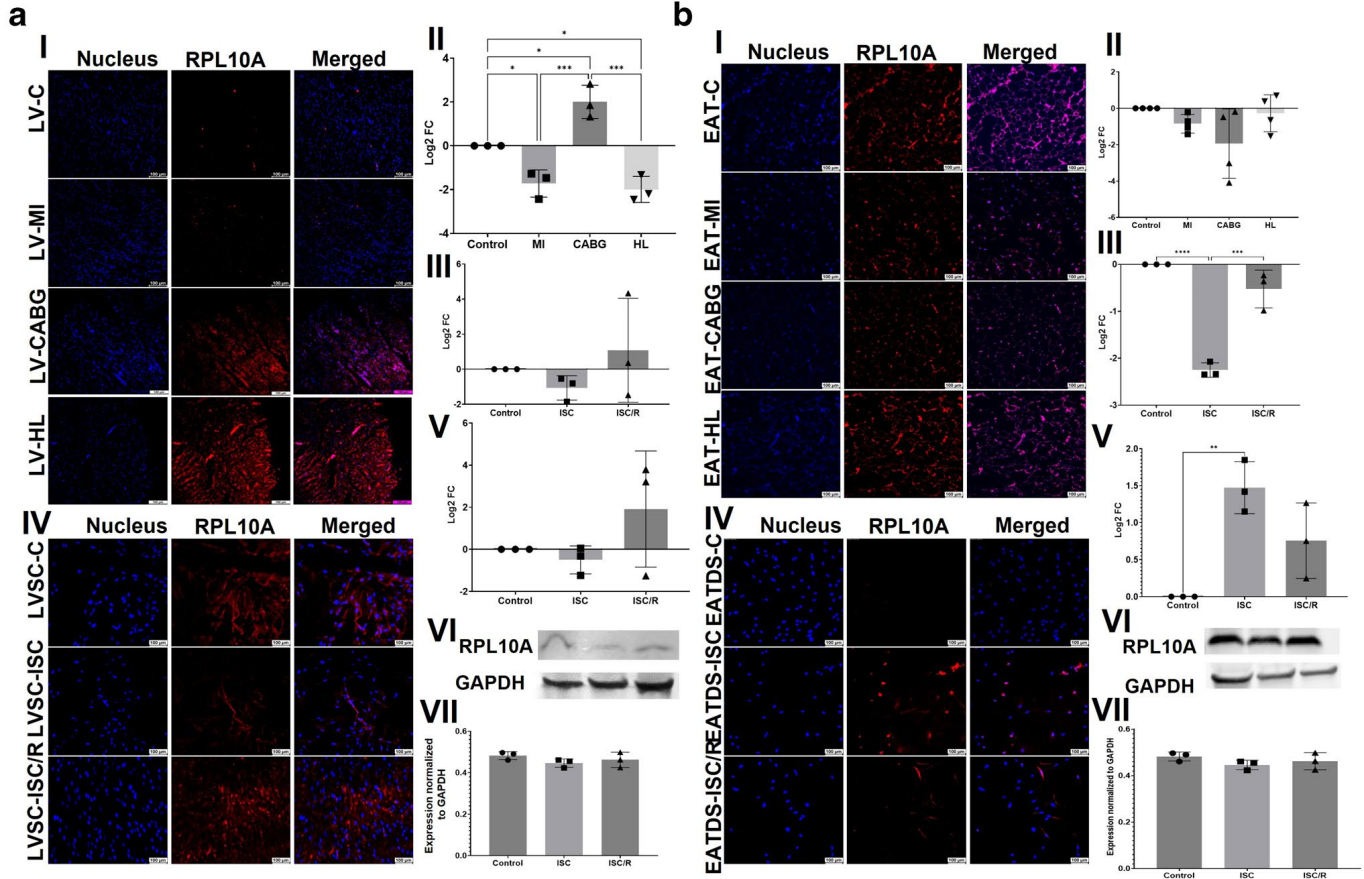
**a** Representative images for the immunofluorescence analysis for the expression of RPL10A in the LV tissues (**4aA-I**) (*N* = 3) and the quantification of protein expression of RPL10A in LV tissues calculated from MFI (**4a-II**). (**4a-III**). Gene expression analysis of RPL10A in LVSC (*N* = 3) following ISC and ISC/R challenge using qRT-PCR relative to the housekeeping gene GAPDH normalized to the control and presented as Log_2_ FC. Representative images for the immunofluorescence analysis for the expression of RPL10A in the LVSC (**4a-IV**) (*N* = 3) following the treatment with ISC and ISC/R and (**4a.V**) the quantification of protein expression calculated from MFI. (**4a.VI**) Representative image of Western blot showing the expression status of RPL10A in the ISC and ISC/R groups compared to the control in LVSC (*N* = 3) and (**4a.VI**) quantification represented as fold-change relative to GAPDH normalized to control. **b** Representative images for the immunofluorescence analysis for the expression of RPL10A in the EAT (**4b-I**) (*N* = 4) and the quantification of protein expression of RPL10A in EAT tissues calculated from MFI (**4b-II**). (**4b-III**). Gene expression analysis of RPL10A in EATDS (*N* = 3) following ISC and ISC/R challenge using qRT-PCR relative to the housekeeping gene GAPDH normalized to the control and presented as Log_2_ FC. Representative images for the immunofluorescence analysis for the expression of RPL10A in the EATDS (**4b-IV**) (*N* = 3) following the treatment with ISC and ISC/R and (**4b.V**) the quantification of protein expression calculated from MFI. (**4b.VI**) Representative image of Western blot showing the expression status of RPL10A in the ISC and ISC/R groups compared to the control in EATDS (*N* = 3) and (**4b.VII**) quantification represented as fold-change relative to GAPDH normalized to control

The level of RPL10A was significantly decreased in both LV-MI (*P* = 0.0256) and LV-HL (*P* = 0.0118) and was significantly increased in LV-CABG (*P* = 0.0113) compared to the control. Also, the LV-CABG displayed significantly higher expression of RPL10A compared to LV-MI (*P* = 0.0002) and LV-HL (*P* = 0.0001) groups and the variation between LV-MI and LV-HL group was statistically not significant (*P* = 0.9371) (Fig. 4a-I and a-II). The mRNA transcripts of RPL10A were decreased in the ISC (*P* = 0.7474) and ISC/R LVSC (*P* = 0.7460) compared to the control. The extent of decrease was more in the ISC/R (*P* = 0.3584) group; however, was statistically not significant compared to the ISC group (Fig. 4a-III). Similarly, the level of RPL10A was decreased in the ISC group (*P* = 0.9264) of cultured LVSC and increased in the ISC/R group (*P* = 0.3854) compared to the control and was decreased in the ISC group than ISC/R group (*P* = 0.2464) as evident from immunostaining; however, the alterations were statistically not significant (Fig. 4a-IV and a-V). Western Blot analysis of RPL10A in LVSC displayed similar level of expression in ISC (*P* = 0.2932) and ISC/R (*P* = 0.6357) when compared to control and ISC; however, were statistically not significant. ISC and ISC/R groups displayed similar level of RPL10A (*P* = 0.7616) (Fig. 4a-VI and a-VII).

RPL10A was decreased in EAT-MI (*P* = 0.7058), EAT-CABG (*P* = 0.1157) and EAT-HL (*P* = 0.9845) groups compared to the control; however, the alterations among the groups were statistically not significant (Fig. 4b-I and b-II). The mRNA transcripts of RPL10A were decreased in the ISC (*P* < 0.0001) and ISC/R EATDS (*P* = 0.0912) compared to the control; however, the decrease in ISC/R group was statistically not significant. Also, the extent of decrease was significantly more in the ISC/R (*P* = 0.0004) compared to ISC group (Fig. 4b-III). Immunostaining revealed that RPL10A was increased in the ISC (*P* = 0.0056) and ISC/R EATDS (*P* = 0.0908) compared to the control; however, the increase in ISC/R group was statistically not significant. Also, the extent of increase was not significantly more in the ISC (*P* = 0.1085) compared to ISC/R group (Fig. 4b-IV and b-V). Western Blot analysis of RPL10A in EATDS displayed similar level of expression in ISC (*P* = 0.2932) and ISC/R (*P* = 0.6357). Also, the level of RPL10A was not significantly decreased in ISC/R (*P* = 0.7616) compared to ISC (Fig. 4b-VI and b-VII).

### RPL14

**Fig. 5:**
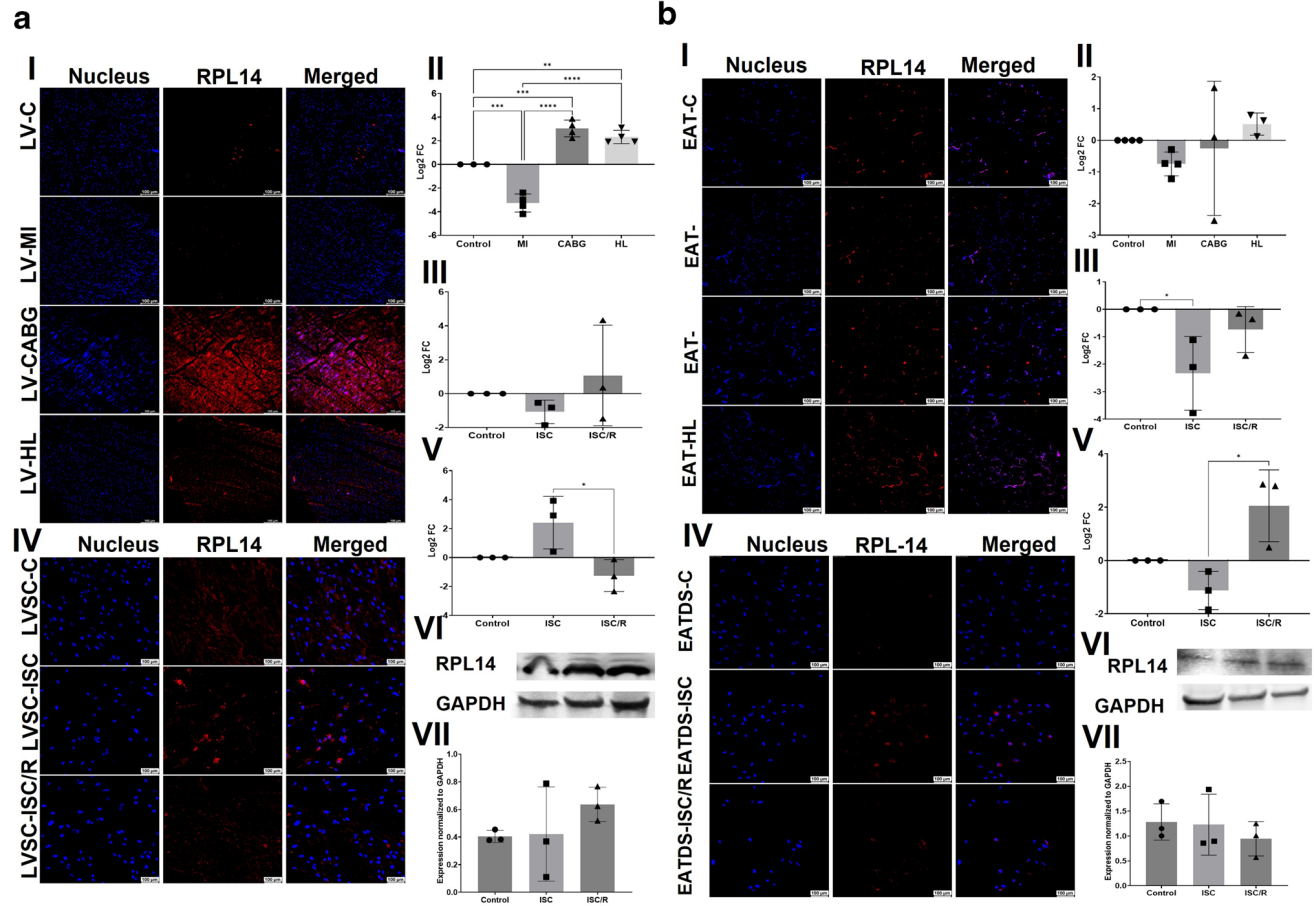
**a** Representative images for the immunofluorescence analysis for the expression of RPL14 in the LV tissues (**5a-I**) (*N* = 3) and the quantification of protein expression of RPL14 in LV tissues calculated from MFI (**5a-II**). (**5a-III**). Gene expression analysis of RPL14 in LVSC (*N* = 3) following ISC and ISC/R challenge using qRT-PCR relative to the housekeeping gene GAPDH normalized to the control and presented as Log_2_ FC. Representative images for the immunofluorescence analysis for the expression of RPL14 in the LVSC (**5a-IV**) (*N* = 3) following the treatment with ISC and ISC/R and (**5a.V**) the quantification of protein expression calculated from MFI. (**5a.VI**) Representative image of Western blot showing the expression status of RPL14 in the ISC and ISC/R groups compared to the control in LVSC (*N* = 3) and (**5a.VI**) quantification represented as fold change relative to GAPDH normalized to control. **b** Representative images for the immunofluorescence analysis for the expression of RPL14 in the EAT (**5b-I**) (*N* = 4) and the quantification of protein expression of RPL14 in EAT tissues calculated from MFI (**5b-II**). (**5b-III**). Gene expression analysis of RPL14 in EATDS (*N* = 3) following ISC and ISC/R challenge using qRT-PCR relative to the housekeeping gene GAPDH normalized to the control and presented as Log_2_ FC. Representative images for the immunofluorescence analysis for the expression of RPL14 in the EATDS (**5b-IV**) (*N* = 3) following the treatment with ISC and ISC/R and (**5b.V**) the quantification of protein expression calculated from MFI. (**5b.VI**) Representative image of Western blot showing the expression status of RPL14 in the ISC and ISC/R groups compared to the control in EATDS (*N* = 3) and (**5b.VII**) quantification represented as fold change relative to GAPDH normalized to control

The level of RPL14 was significantly decreased in LV-MI (*P* = 0.0001) and was significantly increased in LV-CABG (*P* = 0.0002) and LV-HL (*P* = 0.0022) compared to the control. Also, the LV-MI displayed significantly decreased expression of RPL14 compared to LV-CABG (*P* < 0.0001) and LV-HL (*P* < 0.0001) groups and the variation between LV-CABG and LV-HL group was statistically not significant (*P* = 0.3991) (Fig. 5a-I and a-II). The mRNA transcripts of RPL14 were decreased in the ISC (*P* = 0.9617) and ISC/R LVSC (*P* = 0.8716) compared to the control. The extent of decrease was more in the ISC/R (*P* = 0.7323) group; however, was statistically not significant compared to the ISC group (Fig. 5a-III). The level of RPL14 was increased in the ISC group (*P* = 0.1142) of cultured LVSC and decreased in the ISC/R group (*P* = 0.4704) compared to the control; however, the alterations were statistically not significant as evident from immunostaining. Also, RPL14 displayed a significant increase in ISC group than ISC/R group (*P* = 0.0246) (Fig. 5a-IV and a-V). Western Blot analysis of RPL14 in LVSC displayed increased level of expression in ISC (*P* = 0.9947) and ISC/R (*P* = 0.4252) when compared to control; however, were statistically not significant. ISC and ISC/R groups displayed similar level of RPL14 (*P* = 0.4729) (Fig. 5a-VI and a-VII).

RPL14 was decreased in EAT-MI (*P* = 0.7115), EAT-CABG (*P* = 0.9858) and increased in EAT-HL (*P* = 0.9022) groups compared to the control; however, the alterations among the groups were statistically not significant (Fig. 5b-I and b-II). The mRNA transcripts of RPL14 were decreased in ISC (*P* = 0.0466) and ISC/R EATDS (*P* = 0.6120) groups compared to the control; however, the decrease was statistically not significant in ISC/R group. Also, the extent of decrease of RPL14 transcripts in ISC was statistically not significant (*P* = 0.1613) compared to ISC/R group (Fig. 5b-III). Immunostaining revealed that RPL14 was decreased in the ISC (*P* = 0.3296) and increased in the ISC/R EATDS (*P* = 0.0655) compared to the control; however, was statistically not significant. Also, the extent of increase was statistically significant in the ISC (*P* = 0.0107) compared to ISC/R group (Fig. 5b-IV and b-V). Western Blot analysis of RPL14 in EATDS displayed similar level of expression in ISC (*P* = 0.9884) and ISC/R (*P* = 0.6550) when compared to control and between ISC and ISC/R groups (*P* = 0.7374) (Fig. 5b-VI and b-VII).

### RPS30 (FAU-40)

**Fig. 6:**
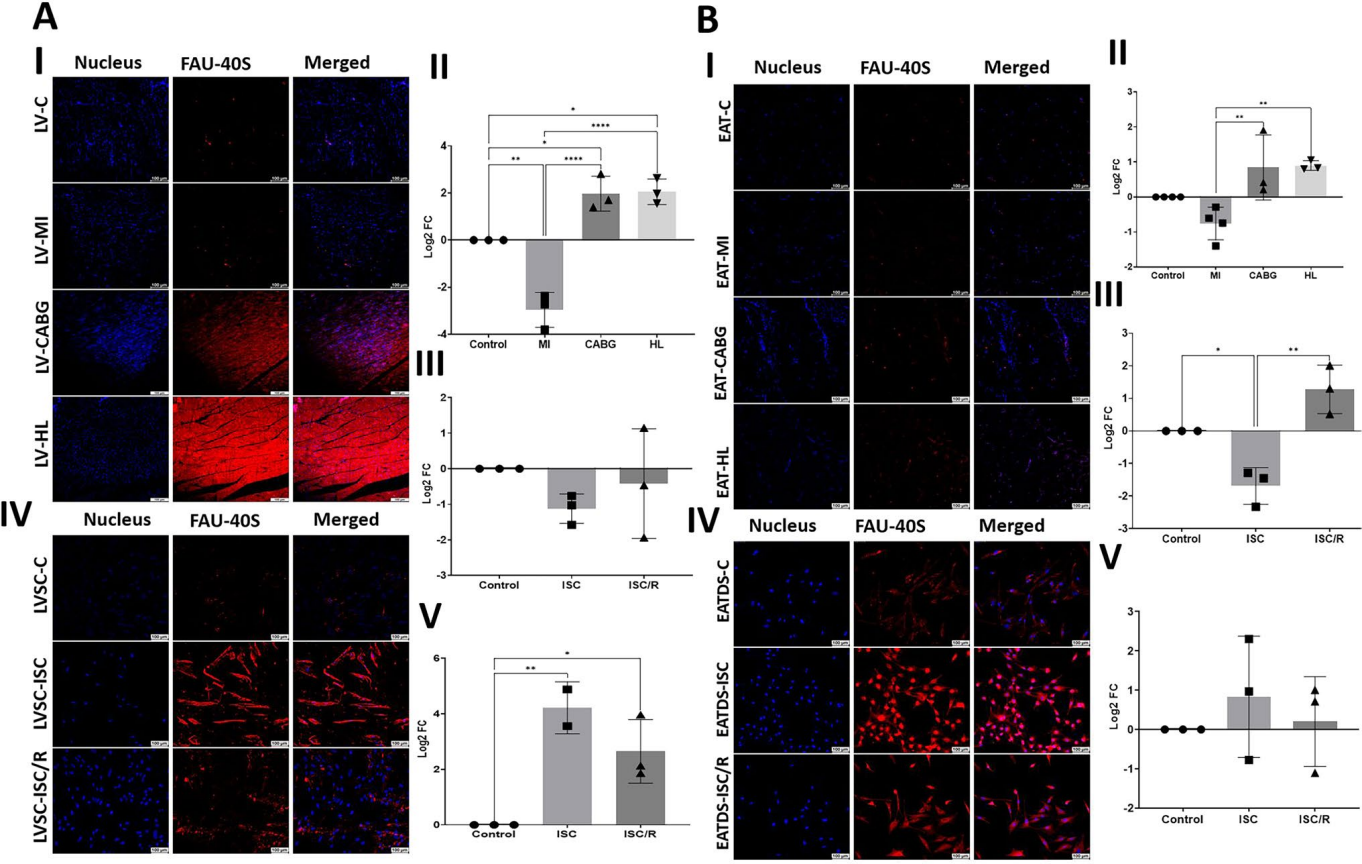
**a** Representative images for the immunofluorescence analysis for the expression of RPS30 (FAU-40) in the LV tissues (**6a-I**) (*N* = 3) and the quantification of protein expression of RPS30 in LV tissues calculated from MFI (**6a-II**). (**6a-III**). Gene expression analysis of RPS30 in LVSC (*N* = 3) following ISC and ISC/R challenge using qRT-PCR relative to the housekeeping gene GAPDH normalized to the control and presented as Log_2_ FC. Representative images for the immunofluorescence analysis for the expression of RPS30 in the LVSC (**6a-IV**) (*N* = 3) following the treatment with ISC and ISC/R and (**6a.V**) the quantification of protein expression calculated from MFI. **b** Representative images for the immunofluorescence analysis for the expression of RPS30 in the EAT (**6b-I**) (*N* = 4) and the quantification of protein expression of RPS30 in EAT tissues calculated from MFI (**6b-II**). (**6b-III**) Gene expression analysis of RPS30 in EATDS (*N* = 3) following ISC and ISC/R challenge using qRT-PCR relative to the housekeeping gene GAPDH normalized to the control and presented as Log_2_ FC. Representative images for the immunofluorescence analysis for the expression of RPS30 in the EATDS (**6b-IV**) (*N* = 3) following the treatment with ISC and ISC/R and (**6b.V**) the quantification of protein expression calculated from MFI

The level of RPS30 was significantly decreased in LV-MI (*P* = 0.0013) and significantly increased in LV-HL (*P* = 0.0120) and LV-CABG (*P* = 0.0148) compared to the control. Also, the LV-MI displayed significantly decreased expression of RPS30 compared to LV-CABG (*P* < 0.0001) and LV-HL (*P* < 0.0001) groups and the variation between LV-CABG and LV-HL groups was statistically not significant (*P* = 0.9985) (Fig. 6a-I and a-II). The mRNA transcripts of RPS30 were decreased in the ISC (*P* = 0.3586) and ISC/R LVSC (*P* = 0.8493) compared to the control. The extent of decrease was more in the ISC (*P* = 0.6371) group; however, was statistically not significant compared to the ISC/R group (Fig. 5a-III). The level of RPS30 was significantly increased in the ISC (*P* = 0.0062) and ISC/R (*P* = 0.0262) groups of cultured LVSC compared to the control; however, the alterations between ISC and ISC/R groups were statistically not significant (*P* = 0.1954) as evident from immunostaining (Fig. 5a-IV and a-V).

RPS30 expression was decreased in EAT-MI (*P* = 0.1925) and increased in EAT-CABG (*P* = 0.1762) and EAT-HL (*P* = 0.1418) groups compared to the control; however, were statistically not significant. Also, the LV-MI displayed significantly decreased expression of RPS30 compared to LV-CABG (*P* = 0.0075) and LV-HL (*P* = 0.0060) groups and the variation between LV-CABG and LV-HL groups was statistically not significant (*P* = 0.9990) (Fig. 5b-I and b-II). The mRNA transcripts of RPS30 were significantly decreased in ISC (*P* = 0.0200) and increased in ISC/R EATDS (*P* = 0.0613) groups compared to the control; however, the increase was statistically not significant in ISC/R group. Also, the extent of decrease of RPS30 transcripts in ISC was statistically significant (*P* = 0.0013) compared to ISC/R group (Fig. 5b-III). Immunostaining revealed that RPS30 was increased in both ISC (*P* = 0.6503) and ISC/R EATDS (*P* = 0.9728) compared to the control; however, was statistically not significant. Also, the extent of expression was statistically not significant in the ISC (*P* = 0.7762) compared to ISC/R group (Fig. 5b-IV and 5b-V).

### Pathway analysis

The KEGG pathway analysis (based on the altered expression of key genes) revealed that RPS18 + LVSCs in Cluster 1 favored cell cycle by upregulating the DNA synthesis suggesting a proliferative population (Supplementary Fig. 1). Additionally, the RPS18 + LVSCs in Cluster 1 operate via TLR/s, JAK-STAT, and PI3K-Akt pathways resulting in cytokine surge and signaling, chemotaxis and activation of inflammatory cells, and complement activation suggest the proinflammatory responses (Supplementary Fig. 2). Hence, Cluster 1 cells represent proliferative pro-inflammatory phenotype. Interestingly, Cluster 2 RPS18 + LVSCs (Supplementary Fig. 3) displayed the pathways involved in glucose metabolism suggesting metabolically active phenotypes capable of withstanding the deleterious effects of ischemia. Cluster 3 RPS18 + LVSCs displayed cell cycle dependent proliferative phenotypes as in Cluster 1 cells (Supplementary Fig. 4); however, without proinflammatory features. Hence, Cluster 3 cells represent actively proliferating LVSCs. Importantly, Cluster 4 RPS18 + LVSCs displayed hypoxia responsive pathways via HIF1α and HIF1β signaling characterized with the operation of biological events associated with increased oxygen delivery (erythropoiesis, iron metabolism and angiogenesis) with a concomitant reduction in oxygen consuming events (promoting anerobic metabolism, inhibiting TCA cycle, promoting cell proliferation, and inhibiting the apoptosis) (Supplementary Fig. 5). Additionally, Cluster 4 RPS18 + LVSCs revealed cell proliferation signaling via accelerating the DNA synthesis similar to Cluster 1 and Cluster 3 cells (Supplementary Fig. 6). Hence, Cluster 4 RPS18 + LVSCs represent a pro-survival/regenerative phenotype capable of withstanding the pathological events associated with ischemic injury. Further characterization of Cluster 4 RPS18 + LVSCs is warranted for unveiling the regenerative mechanisms and their translational applications for regenerative cardiology.

The KEGG pathway analysis for Cluster 1 RPSA + LVSCs were characterized by the pathways associated with glucose metabolism suggesting metabolically active phenotypes like Cluster 2 RPS18 + LVSCs (Supplementary Fig. 7). Additionally, RPSA + LVSCs in Cluster 2 favored cell cycle by upregulating the DNA synthesis suggesting a proliferative population similar to Cluster 1 RPS18 + LVSCs (Supplementary Fig. 8). Importantly, Cluster 3 RPSA + LVSCs displayed TNF signaling wit TNFR1 via downstream MAPK signaling resulting in leukocyte recruitment and activation, cytokine release, intercellular adhesion and communication, ECM remodeling, and vascular effects. Similarly, Cluster 3 RPSA + LVSCs operate through TNFR2 receptors to elicit PI3K-Akt signaling to promote cell survival (Supplementary Fig. 9). Also, RPSA + LVSCs in Cluster 3 favored cell cycle by upregulating the DNA synthesis suggesting a proliferative population similar to Cluster 2 (Supplementary Fig. 10). These pathways reflect the proliferative pro-survival phenotype of Cluster 3 RPSA + LVSCs which suggests the protective effects in the reperfused cells. Favored by ischemia, Cluster 4 RPSA + LVSCs operate p53 signaling (Supplementary Fig. 11) activating the cell survival responses suggesting a protective phenotype (Ozaki and Nakagawara 2011).

The pathway analysis revealed that Cluster 1 RPL30 + LVSCs operate p53 signaling (Supplementary Fig. 12) activating the cell survival responses suggesting a protective phenotype (Ozaki and Nakagawara 2011). Interestingly, Cluster 2 RPL30 + LVSCs displayed RNA degradation and mRNA stabilization pathways (Supplementary Fig. 13) and hypoxia responsive signaling for cell survival (Supplementary Fig. 14). Additionally, Cluster 2 RPL30 + LVSCs showed ERBB signaling leading to cell proliferation, migration, adhesion, survival, and angiogenesis operating through mTOR pathway and MAPK signaling (Supplementary Fig. 15). The operation of these pathways signifies the cardioprotective Cluster 2 RPL30 + LVSCs phenotype. Cluster 3 and 5 RPL30 + LVSCs displayed the active DNA synthesis pathways suggesting the proliferative phenotype (Supplementary Fig. 16). Cluster 4 RPL30 + LVSCs displayed lipid biosynthesis pathways (Supplementary Fig. 17) and PPAR signaling (Supplementary Fig. 18) to activate lipid metabolism and fatty acid degradation, cell survival, and angiogenesis mediated through multiple adipokine signaling. Since Cluster 4 RPL30 + LVSCs favored ischemia, operation of these pathways suggests the metabolic adaptation of LVSCs under starvation. Cluster 6 RPL30 + LVSCs exhibited hypoxia responsive signaling for cell survival as in Cluster 2 RPL30 + LVSCs (Supplementary Fig. 19). Cluster 7 RPL30 + LVSCs displayed lipid biosynthesis pathways as in Cluster 4 RPL30 + LVSCs (Supplementary Fig. 20). Additionally, Cluster 7 RPL30 + LVSCs operate through TNFR2 receptors to elicit PI3K-Akt signaling to promote cell survival (Supplementary Fig. 21). Contrastingly, the RPL30 + LVSCs in Cluster 7 displayed TLR/s, JAK-STAT, and PI3K-Akt pathways resulting in cytokine surge and signaling suggesting the proinflammatory responses (Supplementary Fig. 22). Hence, Cluster 7 RPL30 + LVSCs represent a viable proinflammatory population with active lipid metabolism.

Interestingly, Cluster 2 RPL30 + EATDS (Supplementary Fig. 23) displayed multiple pathways involved in glucose metabolism suggesting metabolically active phenotypes capable of withstanding the deleterious effects of ischemia following reperfusion. Cluster 3 RPL30 + EATDS showed multiple pro-inflammatory cytokine interactions (Supplementary Fig. 24), chemokine activation for inflammation (Supplementary Fig. 25), and smooth muscle contraction signaling (Supplementary Fig. Supplementary Figure 26) suggesting the pro-inflammatory contractile phenotype. Overall, RPS18 + LVSCs, RPSA + LVSCs, RPL30 + LVSCs and RPL30 + EATDS predominated with diverse pathways suggesting the existence of versatile sub-populations.

## Discussion

Extracellular/ribosomal functions of ribosomal proteins including the regulation of cell growth, proliferation and differentiation and apoptosis have been highlighted in the pathology of multiple diseases; however, the underlying molecular mechanisms are largely unknown (Kang et al. 2021) (Lu et al. 2015) (Wang et al. 2015). Interestingly, the extracellular ribosomal proteins elicit antimicrobial activity by increasing the reactive oxygen species (ROS) in target cells inducing membrane damage and subsequent inactivation/death (Hurtado-Rios et al. 2022). Furthermore, extracellular ribosomal proteins are capable of inducing stem cell like features suggesting their role in the pro-pathogenic and pro-healing potential (Hide et al. 2022). However, the potential extracellular association of ribosomal proteins in MI pathology and ischemic episodes are yet to be unveiled. Recently, we reported the regenerative potential of extracellular ribosomal proteins secreted in the vesicles of EATDS challenged with ischemia (Thankam et al. 2022a). Herein, we aimed in the descriptive analysis regarding the association of major ribosomal proteins (RPL10A, RPL14, RPL30, RPS18, FAU-40/RPS30, and RPSA/LR) contained in the EVs of ischemic EATDS in the infarcted myocardium, EAT and respective stromal cells (EATDS and LVSCs) (Thankam et al. 2022a).

RPS18 has been associated with increased inflammatory response mediated through cofilin, an actin binding protein that increases chemotaxis, superoxide production and phagocytosis in activated phagocytes through cytoskeletal reorganization (Kusui et al. 2004). RPS18 acts directly at the actin binding site of phagocytes under physiological stress and overexpression of RPS18 often lead to early cell cycle arrest (Kusui et al. 2004) (Noma et al. 2017). Additionally, RPS18 demonstrated immune response against pathogens such as bacteria by increasing cell-mediated immune response and upregulating T cells (Chen et al. 2021) (Roy et al. 2020). Our previous findings phenotyped RPS18 + EATDS unveiling their potential role in cardiac regeneration (Thankam et al. 2022a). Interestingly, the trend of RPS18 expression was contrasting in EAT/EATDS and LV/LVSCs suggesting the superior regenerative potential of EATDS. Also, RPS18 + LVSCs were characterized by four distinct sub-populations where the sub-population-1 favored Control, sub-populations-2 and -4 favored ISC/R and sub-population-4 favored ISC groups. The sub-population-4 of LVSCs represents regenerative phenotype as evident from the upregulation of ADAMTS5 which regulates the profibrotic versican (Barallobre-Barreiro et al. 2021), and IGF1 signaling (Heinen et al. 2019). Additionally, MGP was downregulated in the sub-population-4 of LVSCs suggesting an anti-inflammatory phenotype as MGP is increased in severe inflammation and calcification (Brnic et al. 2020) (Viegas et al. 2017) (Thomsen et al. 2010). Further investigations on RPS18 + LVSCs are warranted to elucidate the regenerative/pathological phenotypes and to establish the underlying molecular mechanisms.

RPSA promotes cardiomyocyte viability and differentiation and interacts with basement membrane proteins, particularly integrin, to promote PI3K and FAK activity promoting the embryonic stem cells to differentiate into cardiomyocytes (Knöll et al. 2007). RPSA has been upregulated by TGF-beta, reducing the inflammatory response by regulating apoptosis, fibrosis, chemotaxis, and cell differentiation suggesting the anti-fibrotic and pro-healing effects (Wenzel et al. 2010). Importantly, RPSA is greatly important in cardiac remodeling by facilitating cardiomyocyte stiffness and ultimately allowing for better compliance of cardiac tissue post MI (Wang et al. 2019). Importantly, impaired RPSA increases cardiomyopathy and in cardiomyocyte degeneration, as new cardiomyocytes lose their structure in the absence of laminin (Hochman-Mendez et al. 2020). Similar to RPS18, the trend of RPSA was contrasting in EAT/EATDS and LV/LVSCs and RPSA + EATDS were anti-inflammatory and regenerative as reported in our previous findings (Thankam et al. 2022a). As in RPS18 + LVSCs, RPSA + cells were characterized by four distinct sub-populations where the sub-population-1 favored Control, sub-populations-2 and -4 favored ISC/R and sub-population-4 favored ISC groups. Similarly, the sub-population-4 of LVSCs is characterized by the upregulation of ADAMTS5 (Barallobre-Barreiro et al. 2021), and IGF1 signaling (Heinen et al. 2019) and the downregulation of MGP suggesting an anti-inflammatory regenerative phenotype (Brnic et al. 2020) (Viegas et al. 2017) (Thomsen et al. 2010). Logically, similar mechanisms programing the LVSCs, possibly by upregulating and secreting RPS18 and RPSA to survive the ischemic insults are being activated; however, warranting further research to establish the underlying molecular mechanisms.

RPL30, the house keeping gene, upregulates during stress-associated responses and the knockdown of RPL30 has been increased the level of p53 activity in cells, markedly decreasing the cell cycle (Ruggero and Pandolfi 2003). Also, the RPL30 driven p53 activation occurs through MDM2, a negative regulator of p53 which correlates with cardiomyocyte hypertrophy and apoptosis in end stage human heart failure (Mak et al. 2017). Importantly, RPL30 has demonstrated adverse outcomes with inflammatory disorders including cancer (De Bortoli et al. 2006, p. 8) (Sun et al. 2010). Even though similar trend was observed in the EAT and LV, the level of RPL30 was significantly decreased in EATDS in the ISC group whereas LVSCs displayed similar level of expression compared to the control. Unfortunately, the information on extra ribosomal functions of RPL30 in cardiac ischemia is currently available. Among 7 sub-populations in LVSCs, only the sub-population-4 favored ISC which is characterized by the upregulation of ADAMTS5, and IGF1 signaling as in RPS18 + and RPSA + LVSCs suggesting the similar regenerative phenotype. On the other hand, EATDS displayed 7 sub-populations and the sub-population-1 favoring the control group was significant. The sub-population-1 of EATDS is characterized by the significant upregulation of IFIT1 suggesting a pro-inflammatory phenotype owing to the cardiac inflammatory responses elicited by IFIT1 (Zhang et al. 2022a) (Wang et al. 2020).

RPL10A is reported to be expressed during early injury response and regeneration of the myocardium and is a mediator of cell proliferation, embryogenesis, and organogenesis through Wnt pathway. Also, RPL10A is upregulated and is a major determinant in T-cell development under physiological stress demonstrating its wide range of function (Genuth et al. 2022) (Wang et al. 2018). Considering the protective effects of RPL10A, the significantly increased expression in LV-CABG tissues suggests that persistence of ischemia upregulates RPL10A following the CABG. Unfortunately, the extra ribosomal functions of RPL10A in cardiac tissues are largely unknown. Similarly, despite very few reports regarding the association of RPL14 in cancer progression, the information regarding the extra ribosomal functions of RPL14 is largely unavailable. The EAT, EATDS and LVSCs displayed basal level of RPL14 expression and the increased level of RPL14 in the CABG and HL LV tissues suggests their association with chronic inflammation. Additionally, FAU-40S (RPS30) is a ubiquitin like protein mediated by the pro-apoptotic Bcl-G which in turn is part of the Bcl-2 family of genes that play significant role in aggravating MI pathology (Pickard et al. 2011) (Korshunova et al. 2021); however, the actual function of FAU-40S in cardiac tissue pathology/regeneration is largely unknown. Interestingly, similar level of increase was observed for FAU-40S in the EAT and LV tissues and the respective stromal cells. Even though the association of RPL10A, RPL14 and FAU-40S have been evident from our findings, the underlying molecular mechanisms warrant further detailed investigation. Additionally, these genes were not identified in the database of single cell genomics.

Generally, the cellular phenotypes are determined by the pattern of active signaling pathways based on the differential expression of key genes (Zhang et al. 2015). Interestingly, the stochasticity in gene expression and the resulting signaling drive phenotypic heterogeneity of stromal cells (Műzes and Sipos 2016). Hence, the pathways predominating in the stromal cells can be assessed to define their phenotypes. Notably, the PATHVIEW analysis unveiled multiple phenotypes including secretory, pro-survival and inflammatory based on their transcription profile at single cell resolution. Additionally, the pathway analysis revealed a contractile phenotype of EATDS suggesting the possible differentiation towards cardiomyocyte lineage reflecting their immense cardiac regenerative potential. Overall, the phenotypes elucidated in this study pose multiple signature genes unveiling the opportunities to sort and harvest the unique phenotypes for translational applications.

Interestingly, the trend of ribosomal protein expression in LV MI tissues were significantly lower than the control, whereas most of the CABG and HL tissues displayed increased expression suggesting that the possible influence of hyperlipidemia and chronic inflammation. Also, the alterations of the ribosomal proteins in EAT tissues were basal, except for RPS30, suggesting their housekeeping functions revealing the resistance to pathologic/ischemic insults. Importantly, the ischemic LVSCs displayed closely similar anti-inflammatory and regenerative sub-phenotypes reflecting the protective/survival mechanisms. Unfortunately, the impact of hyperlipidemia and the extra ribosomal functions of ribosomal proteins are largely unknown. However, the findings from our study throw valuable insights into the association of ribosomal proteins in diverse aspects of ischemic myocardial injury. Despite the promising data, our study is not exempted from limitations. This is a descriptive study where the mechanistic aspects on the impact of HL warrants further investigations, smaller size of ischemic tissues hurdled for detailed analysis, ischemic treatment was solely considered in scRNA-seq data owing to the bulk of information and other targets may be possible, lack of technology to quantify the extra ribosomal level of ribosomal proteins, smaller sample size, and unavailability of specific antibodies (especially for RPS30 WB). Nonetheless, our results revealed the association of a panel of ribosomal protein mediators and unveiled unique sub-phenotypes of LVSCs and EATDS in the ischemic myocardial pathology. Further understanding regarding the underlying molecular mechanisms and functions offers immense translational opportunities in the management of ischemic cardiac complications.

## Conclusions

The expression of RPL10A, RPL14, RPL30, RPS18, FAU-40 (RPS30), and RPSA (Laminin Receptor, LR) was assessed in the myocardial and EAT tissues of MI, CABG and HL swine models and in LVSCs and EATDS challenged with ischemia. The expression level of RPL10A, RPL14, RPL30, RPS18, RPS30, and RPSA were significantly upregulated in the LV tissues of CABG and HL swine with a concomitant reduction in MI group. RPS30 displayed similar upregulation in EAT whereas all the expression of other genes was not significantly altered. Additionally, the ischemic LVSCs and EATDS displayed altered expression status of these genes compared to the control. Interestingly, the RPS18 + , RPL30 + and RPSA + LVSCs favored ischemia and revealed similar anti-inflammatory and regenerative sub-phenotypes reflecting the protective/survival mechanisms. Further understanding regarding the underlying molecular mechanisms and functions of these ribosomal proteins offers immense translational opportunities in the management of ischemic cardiac complications.

## Supplementary Information

Below is the link to the electronic supplementary material.

Supplementary file1 (XLSX 3708 KB)

Supplementary file2 (PNG 30 KB)

Supplementary file3 (PNG 30 KB)

Supplementary file4 (PNG 22 KB)

Supplementary file5 (PNG 30 KB)

Supplementary file6 (PNG 29 KB)

Supplementary file7 (PNG 30 KB)

Supplementary file8 (PNG 22 KB)

Supplementary file9 (PNG 29 KB)

Supplementary file10 (PNG 41 KB)

Supplementary file11 (PNG 30 KB)

Supplementary file12 (PNG 35 KB)

Supplementary file13 (PNG 34 KB)

Supplementary file14 (PNG 111 KB)

Supplementary file15 (PNG 29 KB)

Supplementary file16 (PNG 28 KB)

Supplementary file17 (PNG 29 KB)

Supplementary file18 (PNG 33 KB)

Supplementary file19 (PNG 28 KB)

Supplementary file20 (PNG 29 KB)

Supplementary file21 (PNG 33 KB)

Supplementary file22 (PNG 30 KB)

Supplementary file23 (PNG 41 KB)

Supplementary file24 (PNG 21 KB)

Supplementary file25 (PNG 63 KB)

Supplementary file26 (PNG 38 KB)

Supplementary file27 (PNG 30 KB)

Supplementary file28 (DOCX 71 KB)

## Data Availability

No datasets were generated or analysed during the current study.
