# Supplementary figures and images for: Single cell transcriptomics profiling of the stromal cells in the pathologic association of ribosomal proteins in the ischemic myocardium and epicardial fat
Source: Cell Tissue Res. 2024 Dec 6;399(2):173–92. doi: 10.1007/s00441-024-03933-3 (PMC11787193; doi:10.1007/s00441-024-03933-3)

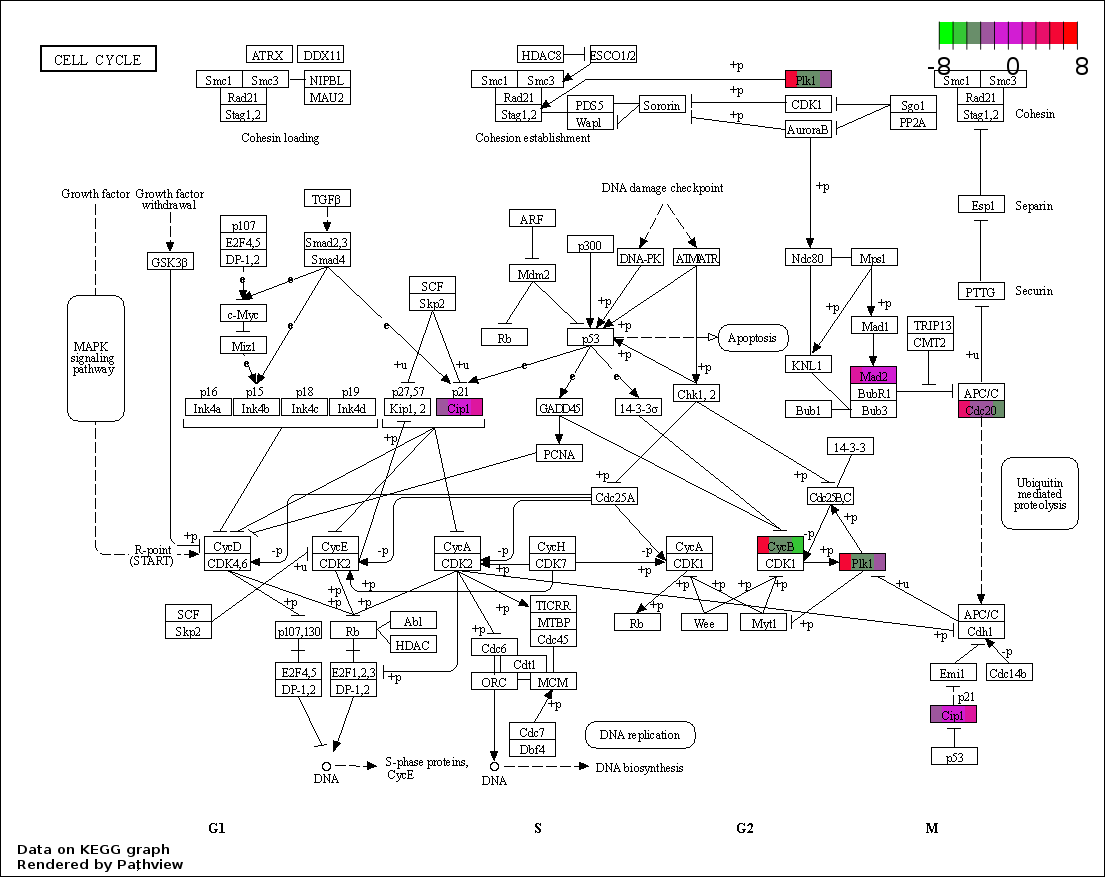

Supplement: Supplementary file 2 — Supplementary file2 (PNG 30 KB) [file 441_2024_3933_MOESM2_ESM.png]

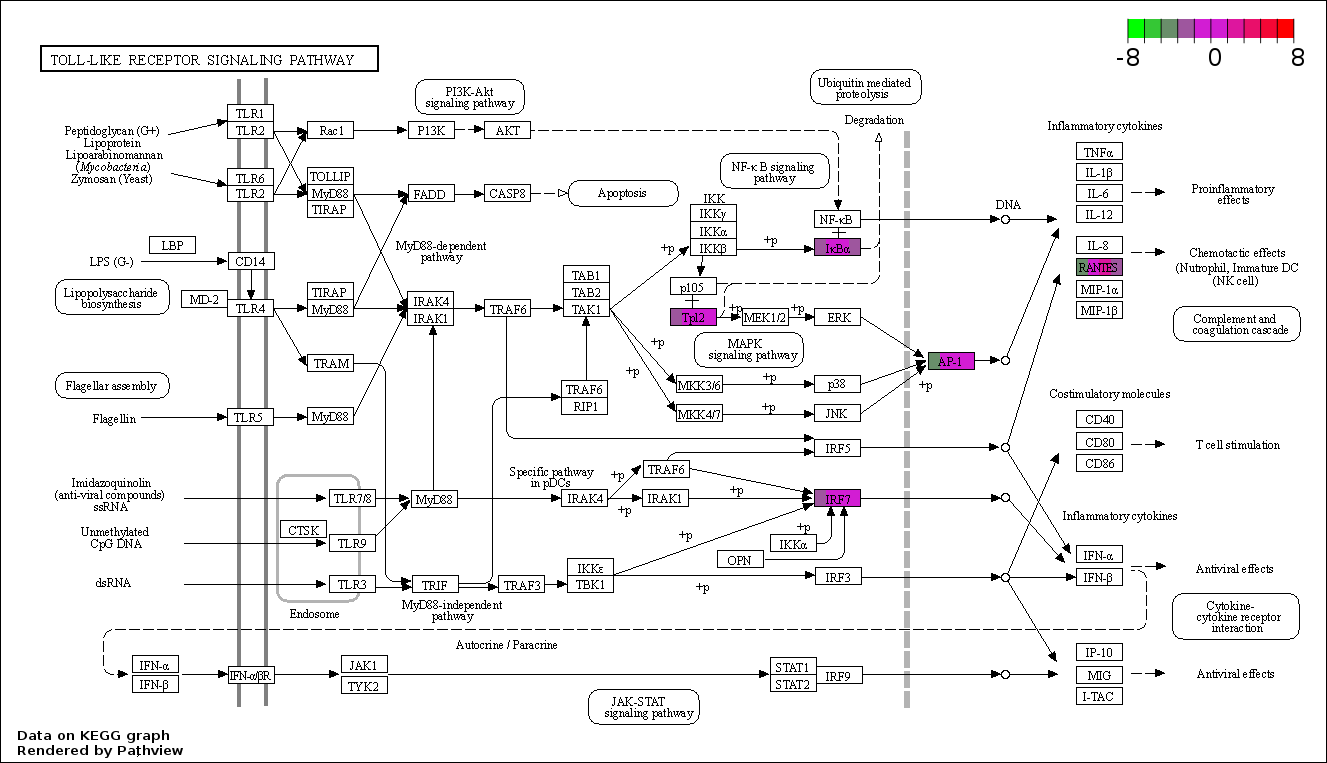

Supplement: Supplementary file 3 — Supplementary file3 (PNG 30 KB) [file 441_2024_3933_MOESM3_ESM.png]

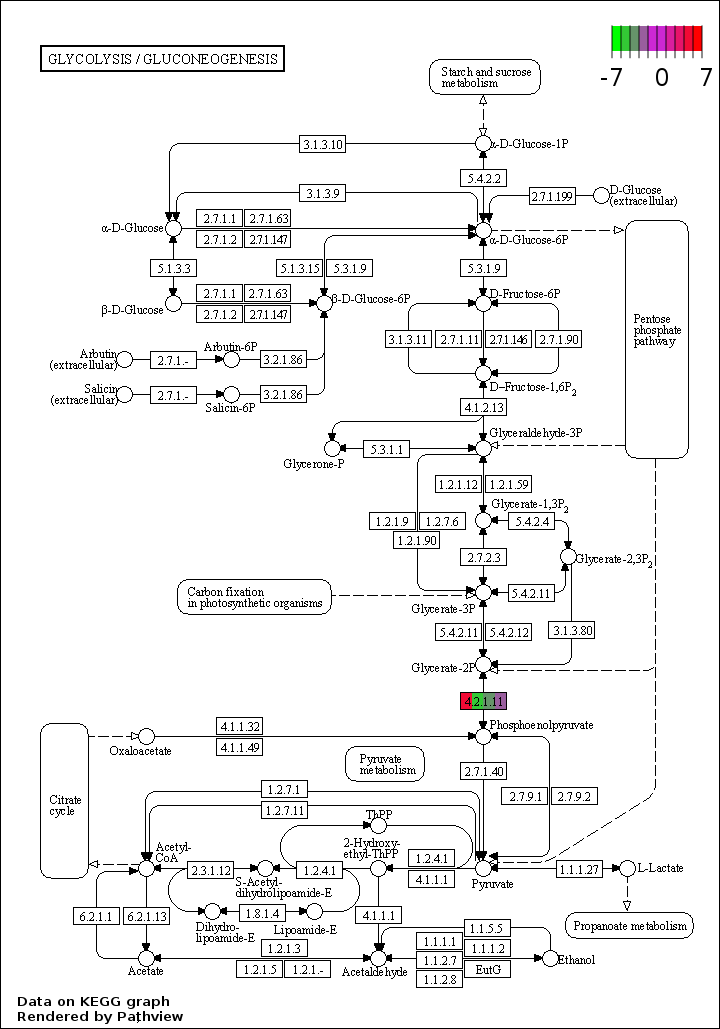

Supplement: Supplementary file 4 — Supplementary file4 (PNG 22 KB) [file 441_2024_3933_MOESM4_ESM.png]

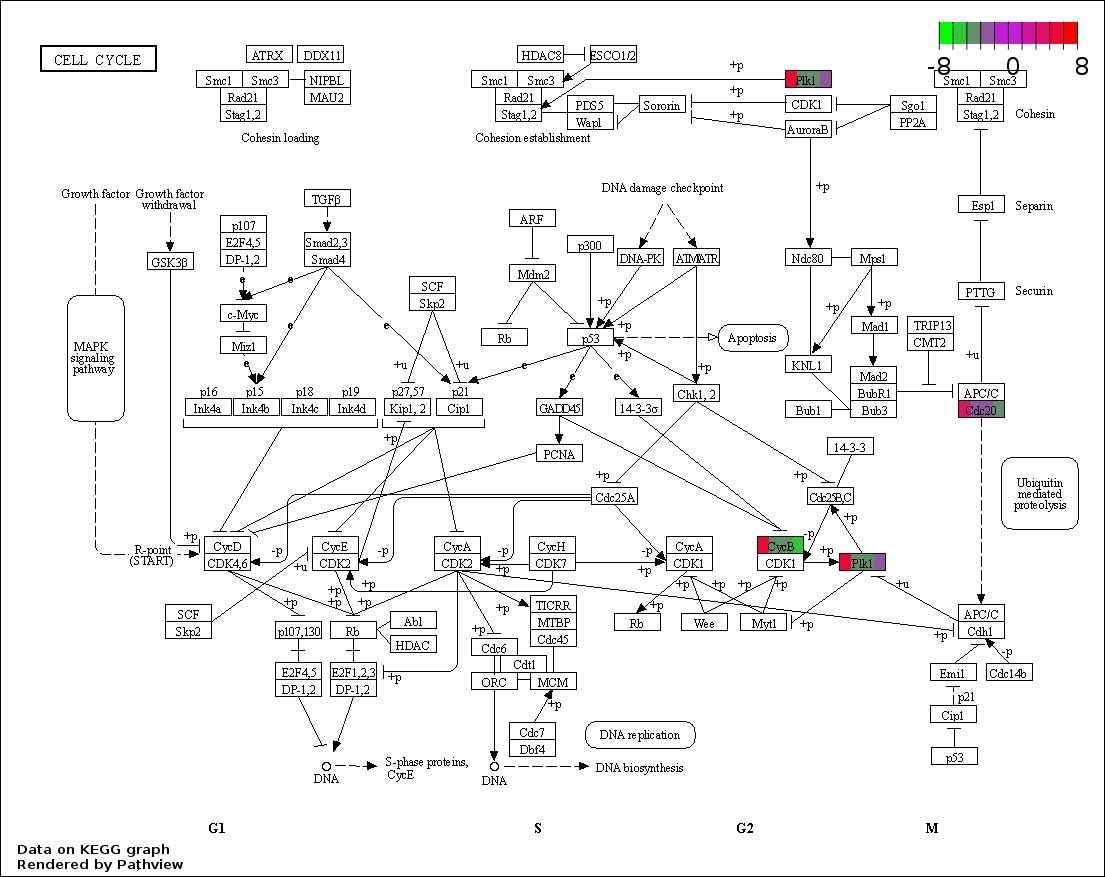

Supplement: Supplementary file 5 — Supplementary file5 (PNG 30 KB) [file 441_2024_3933_MOESM5_ESM.png]

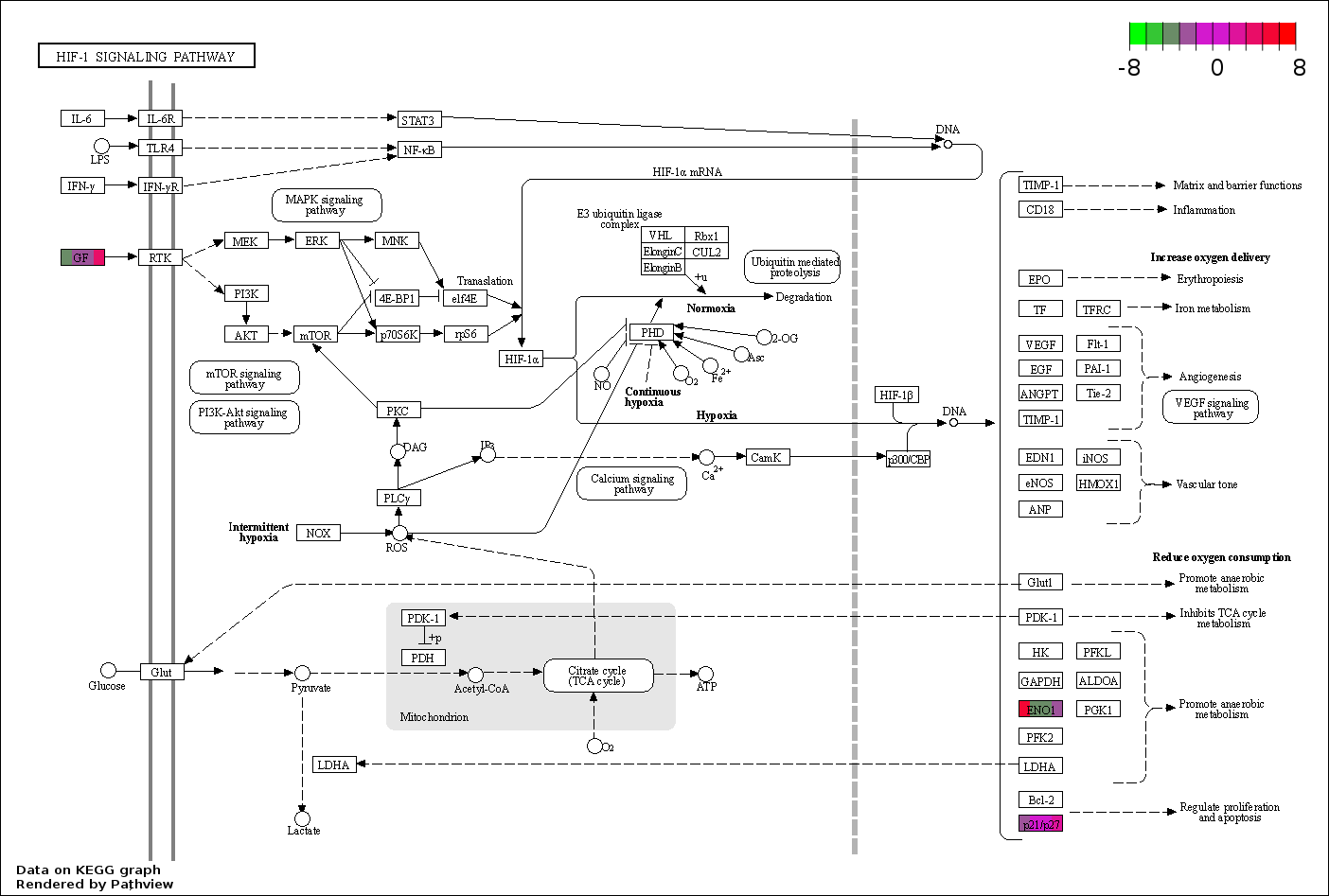

Supplement: Supplementary file 6 — Supplementary file6 (PNG 29 KB) [file 441_2024_3933_MOESM6_ESM.png]

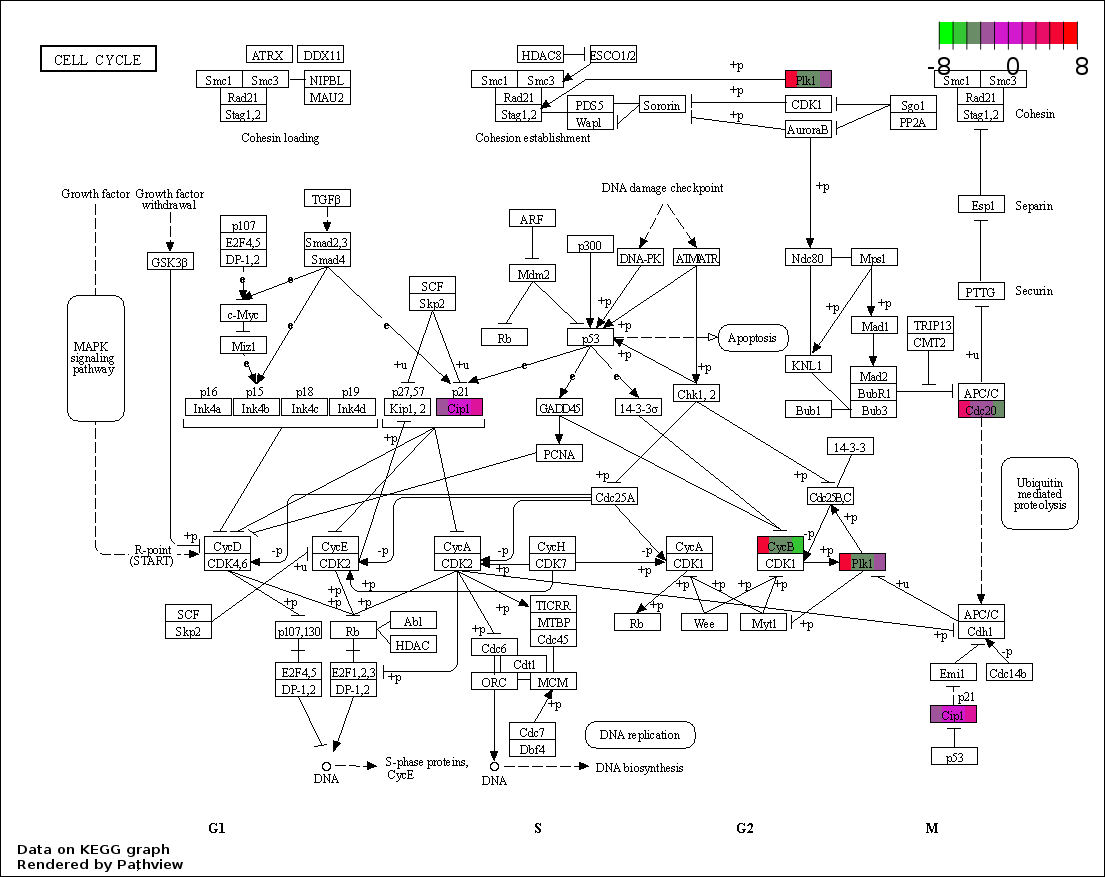

Supplement: Supplementary file 7 — Supplementary file7 (PNG 30 KB) [file 441_2024_3933_MOESM7_ESM.png]

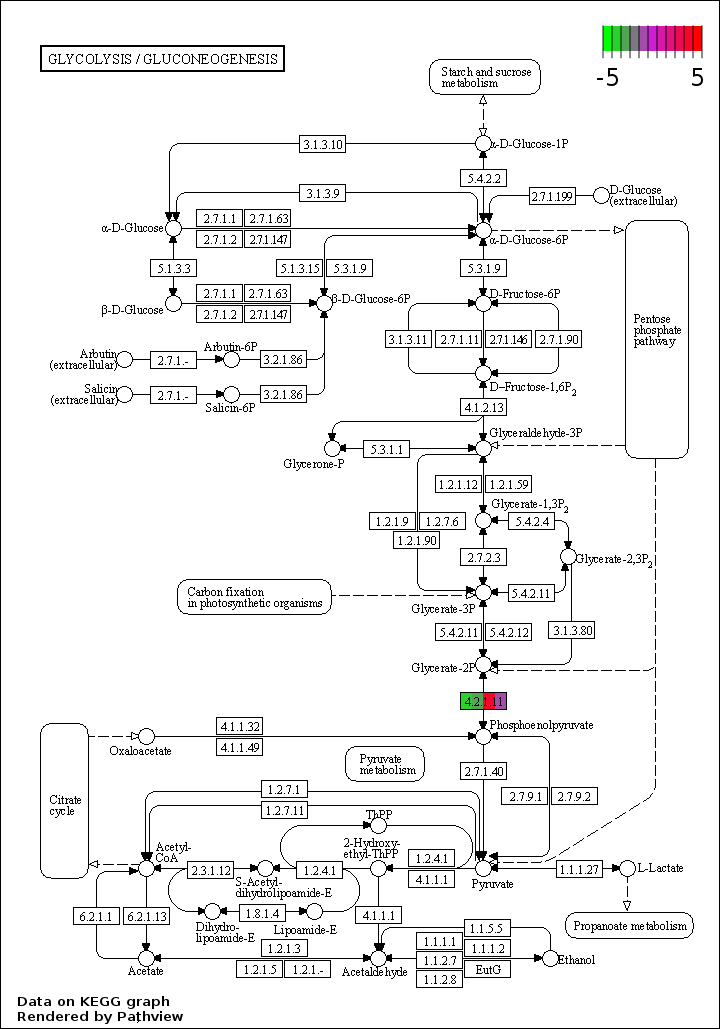

Supplement: Supplementary file 8 — Supplementary file8 (PNG 22 KB) [file 441_2024_3933_MOESM8_ESM.png]

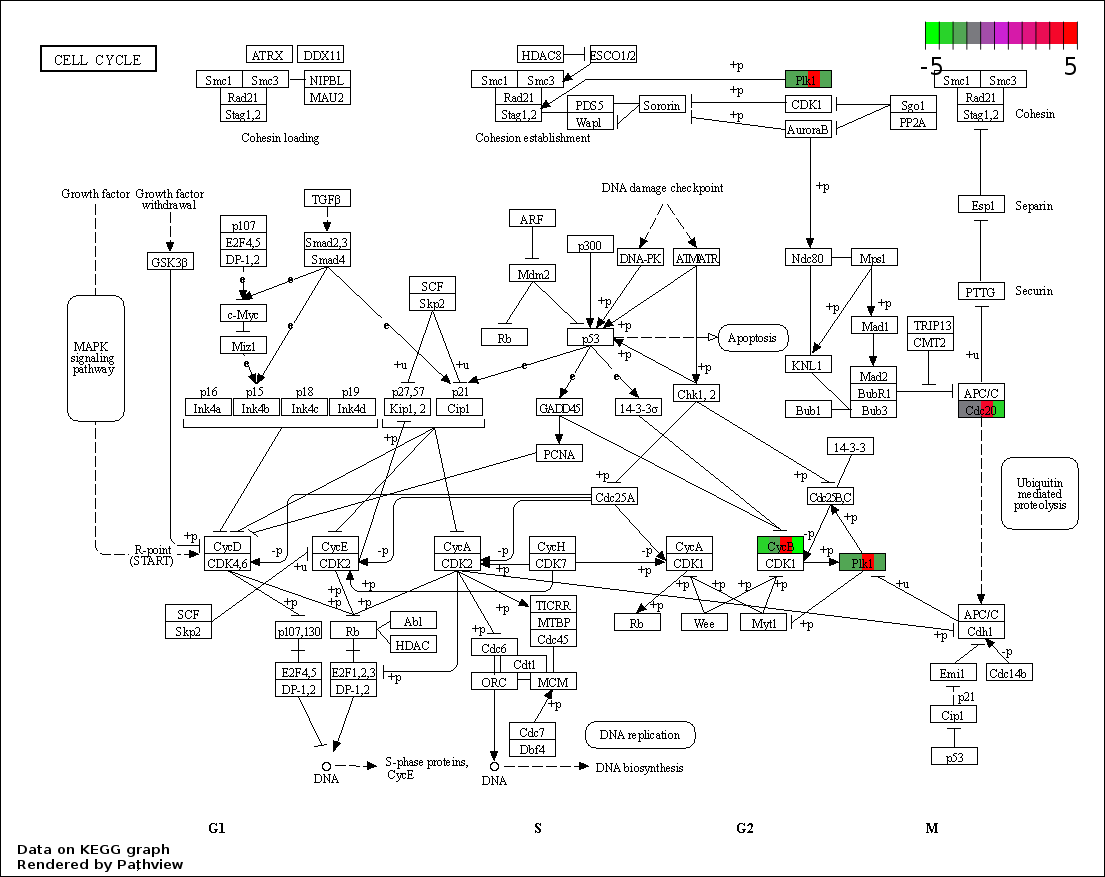

Supplement: Supplementary file 9 — Supplementary file9 (PNG 29 KB) [file 441_2024_3933_MOESM9_ESM.png]

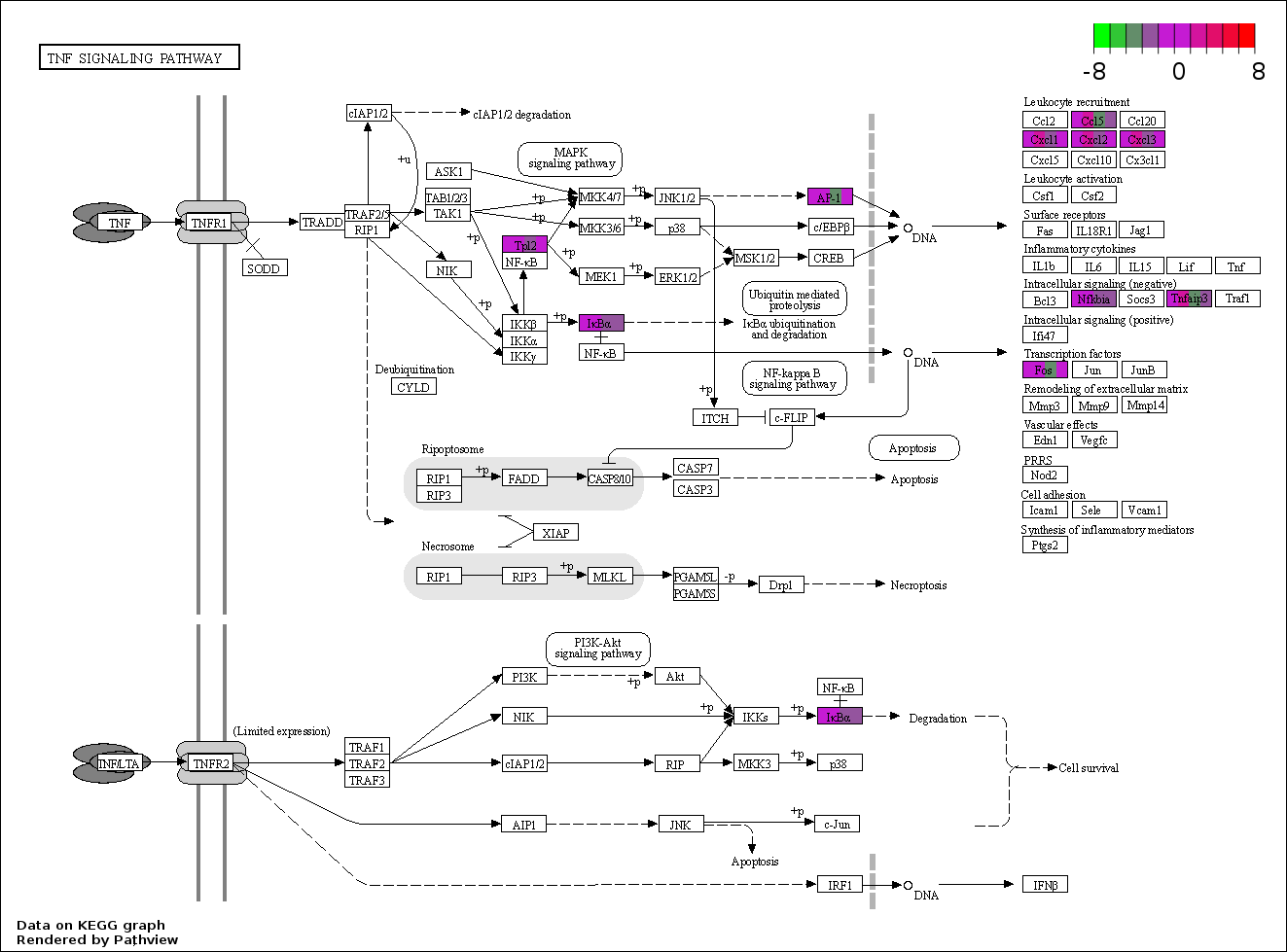

Supplement: Supplementary file 10 — Supplementary file10 (PNG 41 KB) [file 441_2024_3933_MOESM10_ESM.png]

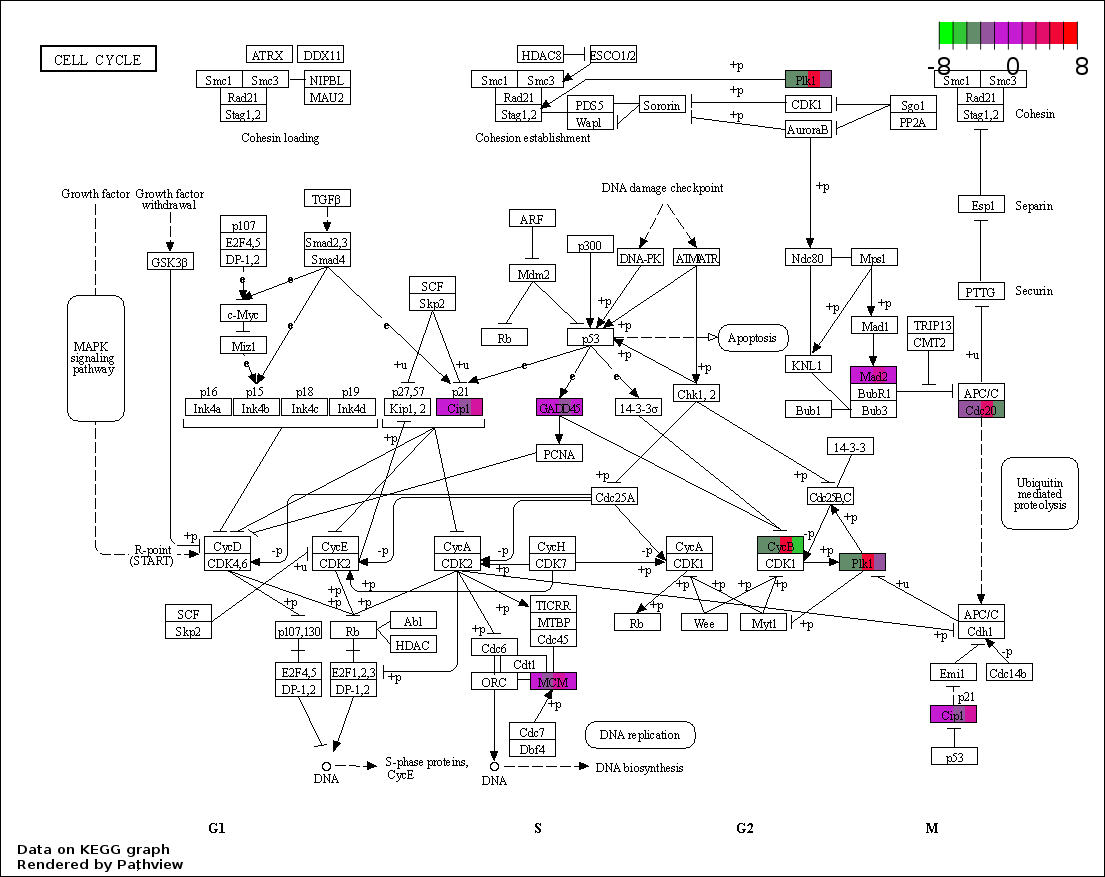

Supplement: Supplementary file 11 — Supplementary file11 (PNG 30 KB) [file 441_2024_3933_MOESM11_ESM.png]

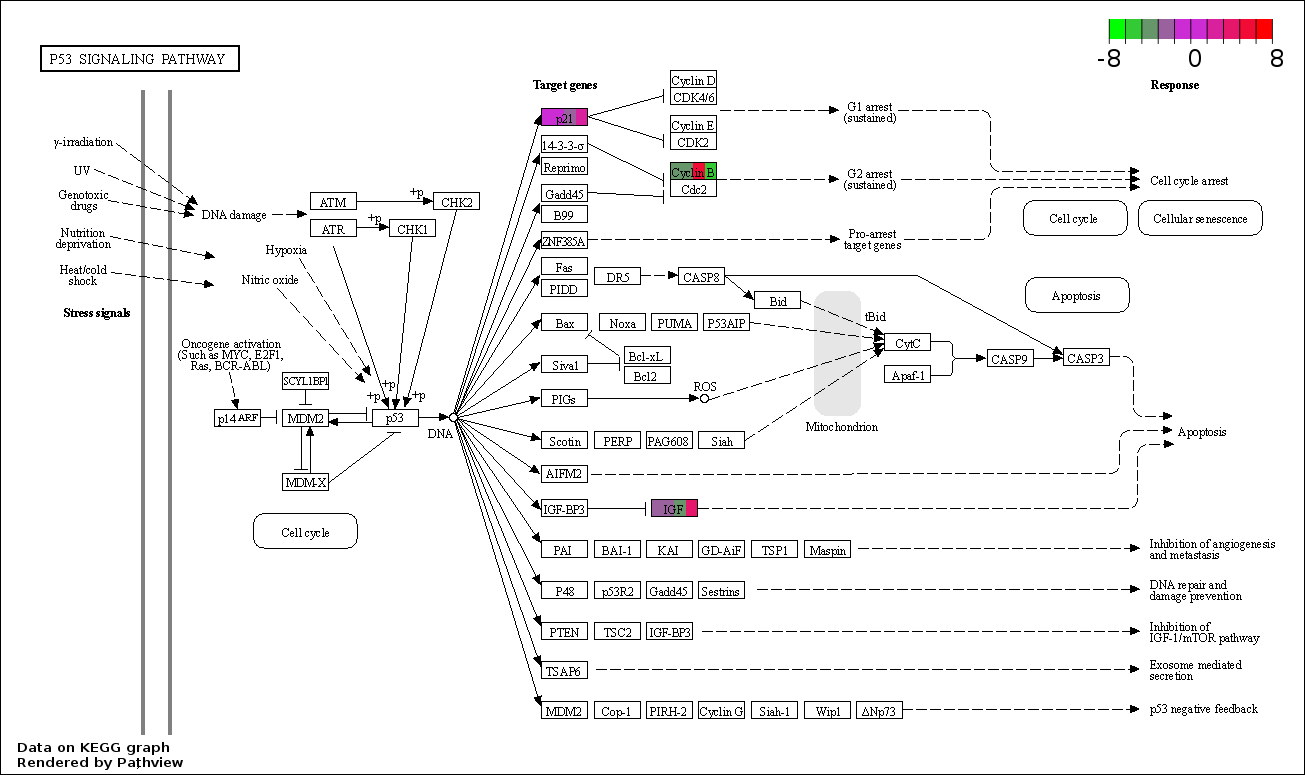

Supplement: Supplementary file 12 — Supplementary file12 (PNG 35 KB) [file 441_2024_3933_MOESM12_ESM.png]

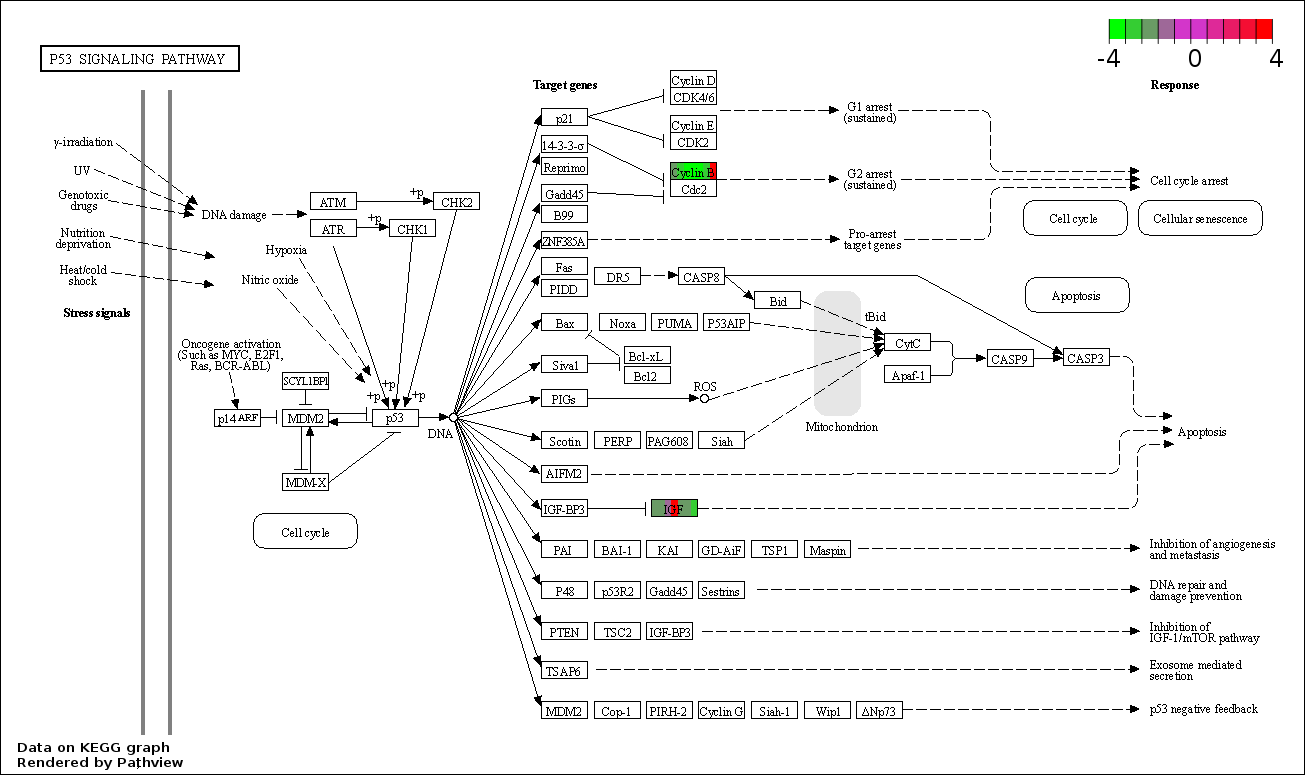

Supplement: Supplementary file 13 — Supplementary file13 (PNG 34 KB) [file 441_2024_3933_MOESM13_ESM.png]

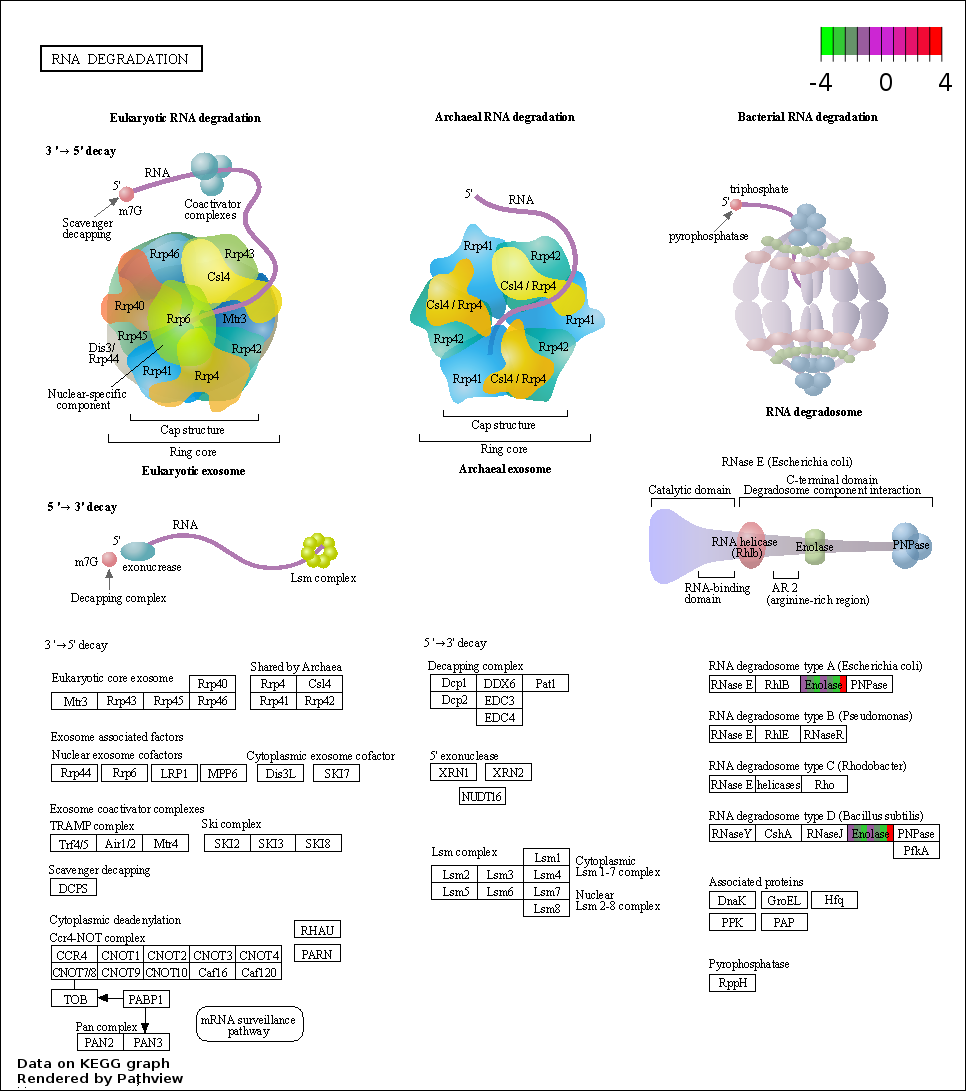

Supplement: Supplementary file 14 — Supplementary file14 (PNG 111 KB) [file 441_2024_3933_MOESM14_ESM.png]

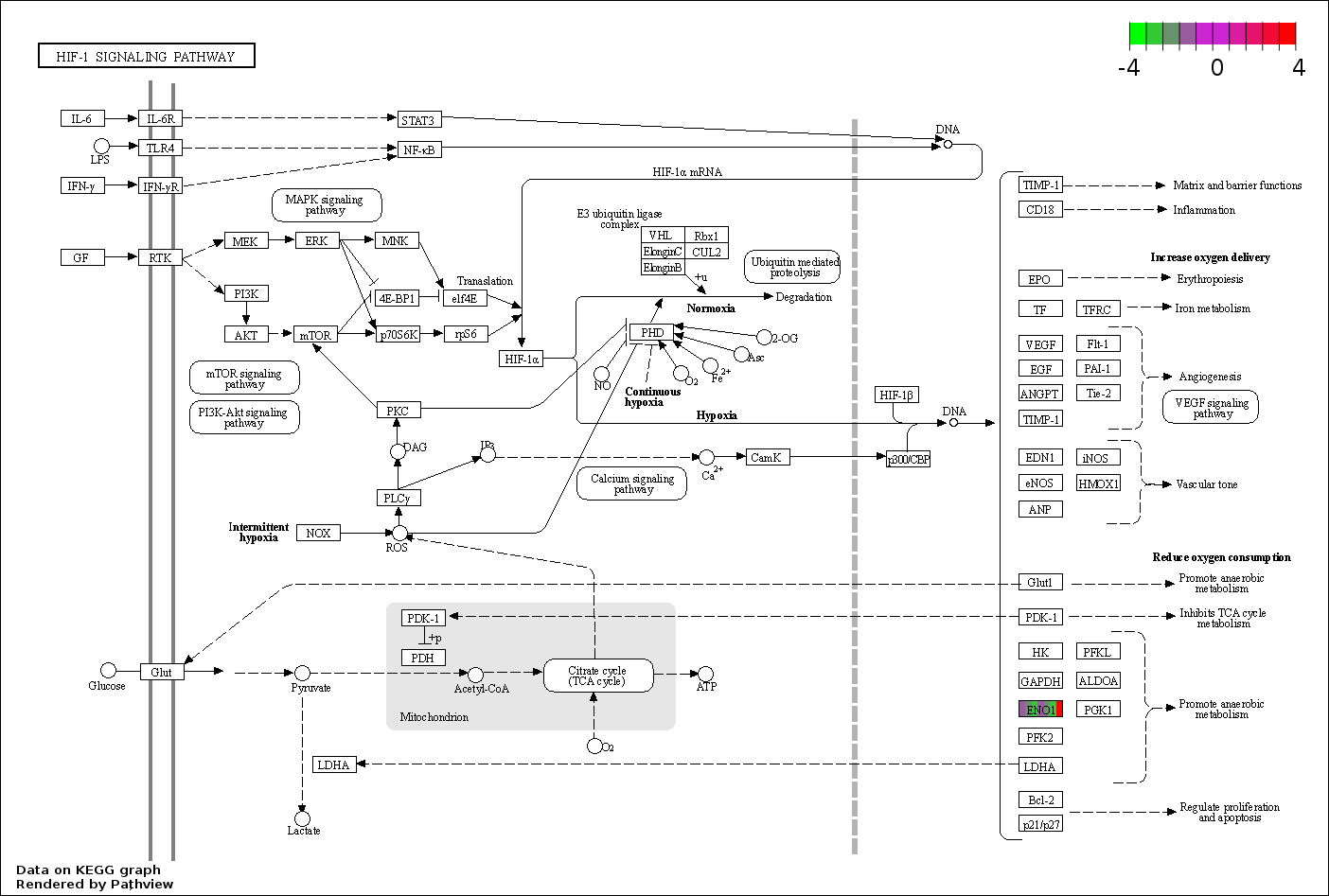

Supplement: Supplementary file 15 — Supplementary file15 (PNG 29 KB) [file 441_2024_3933_MOESM15_ESM.png]

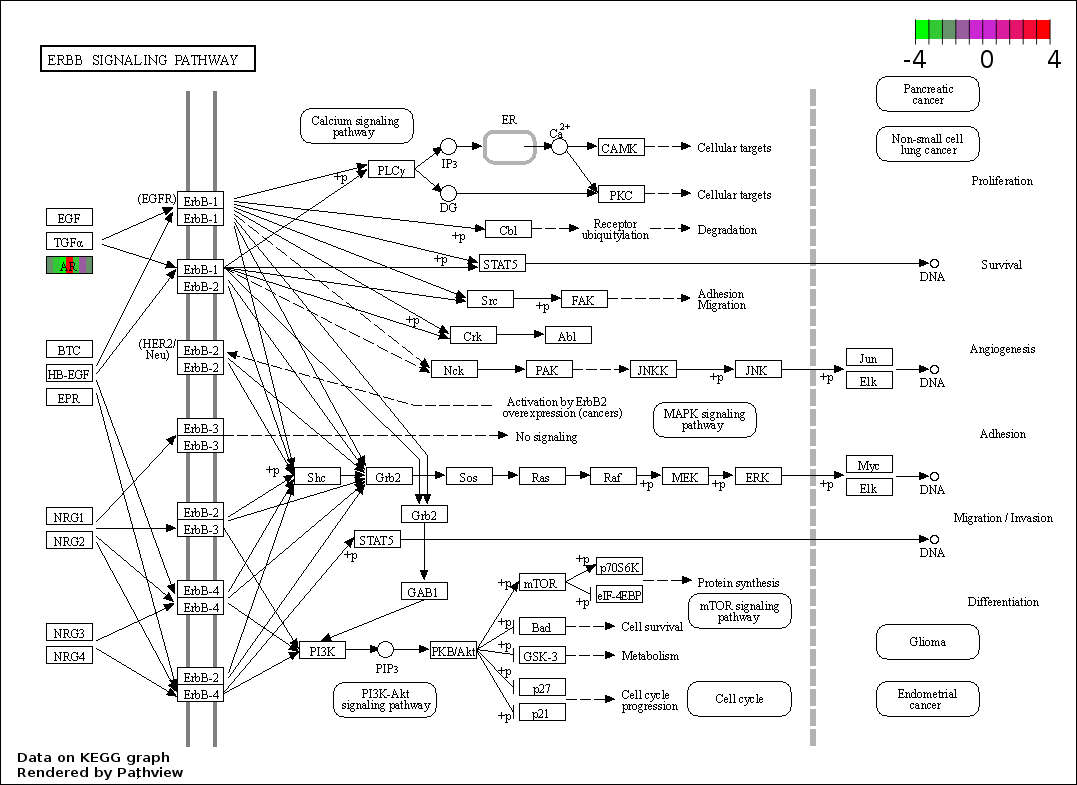

Supplement: Supplementary file 16 — Supplementary file16 (PNG 28 KB) [file 441_2024_3933_MOESM16_ESM.png]

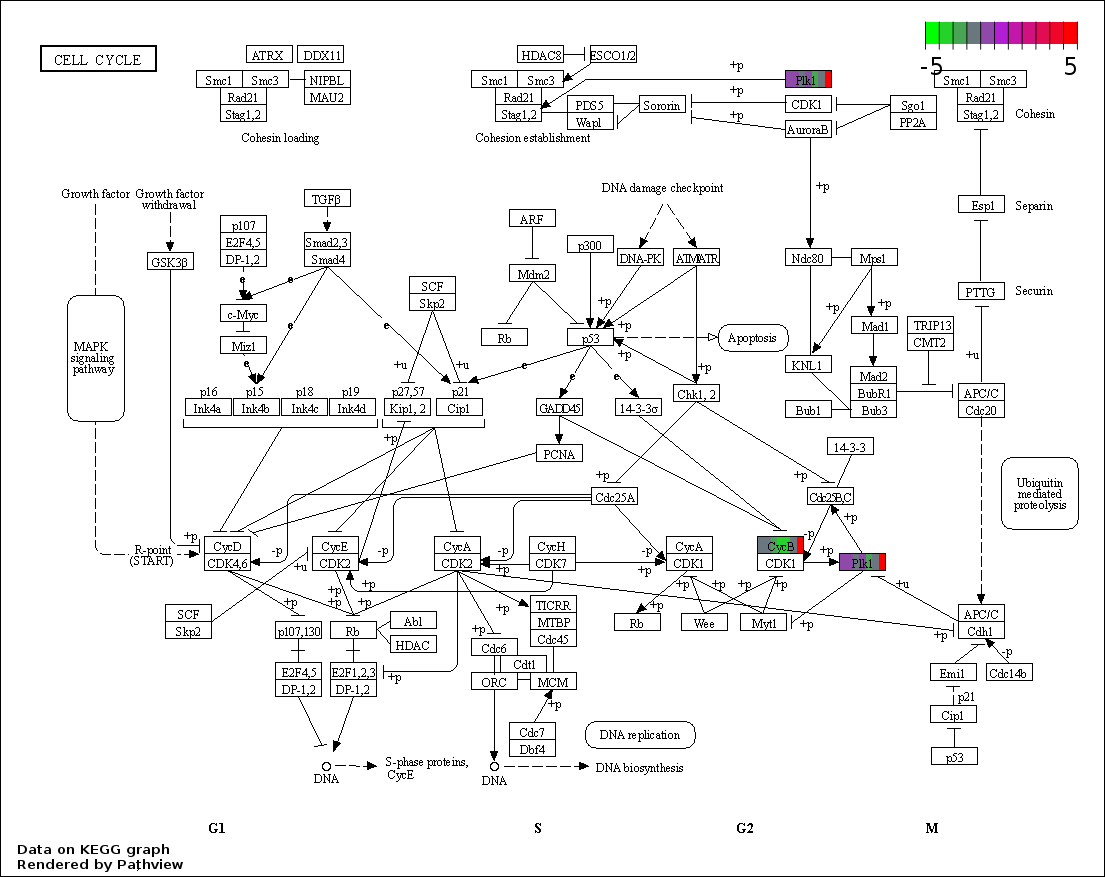

Supplement: Supplementary file 17 — Supplementary file17 (PNG 29 KB) [file 441_2024_3933_MOESM17_ESM.png]

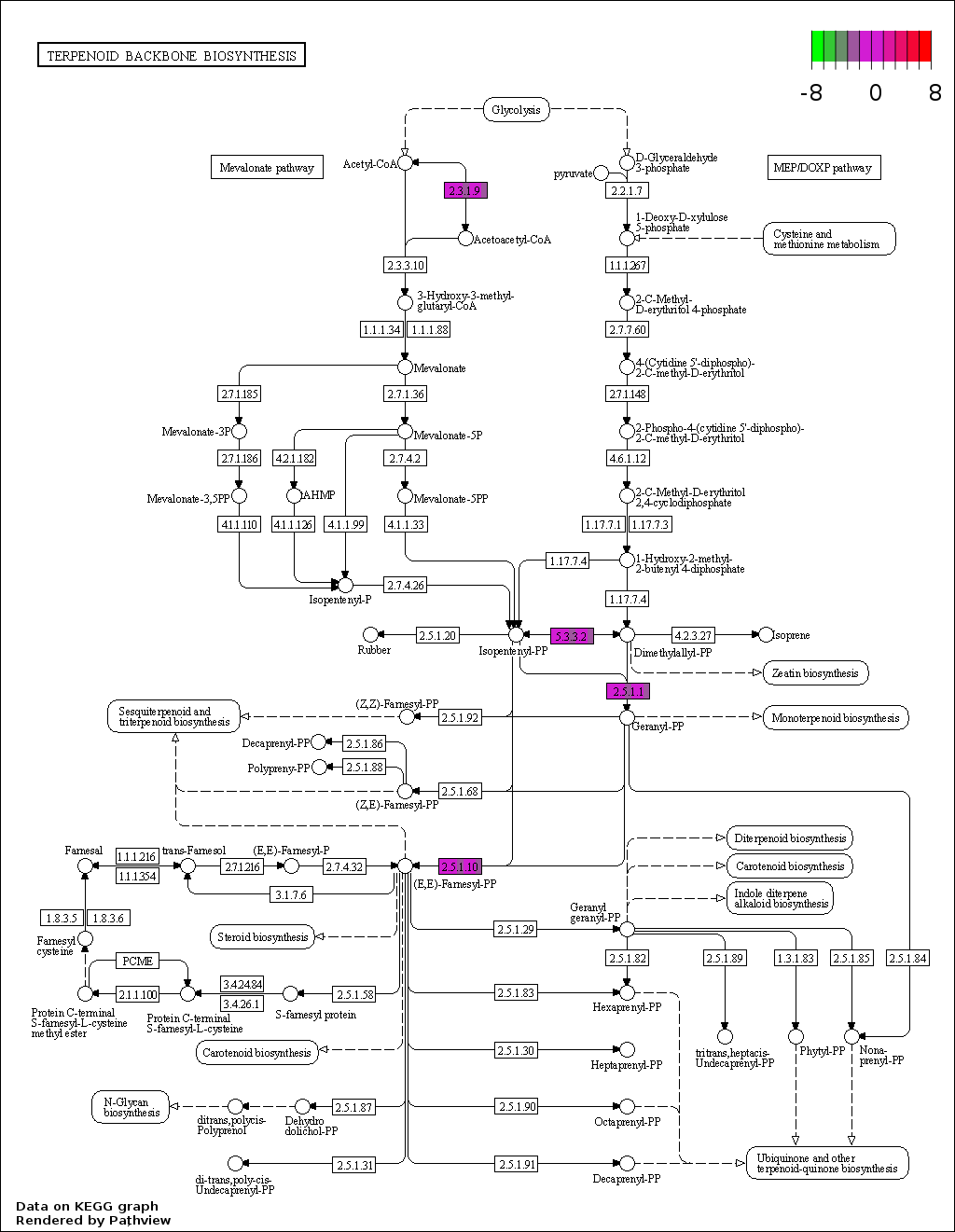

Supplement: Supplementary file 18 — Supplementary file18 (PNG 33 KB) [file 441_2024_3933_MOESM18_ESM.png]

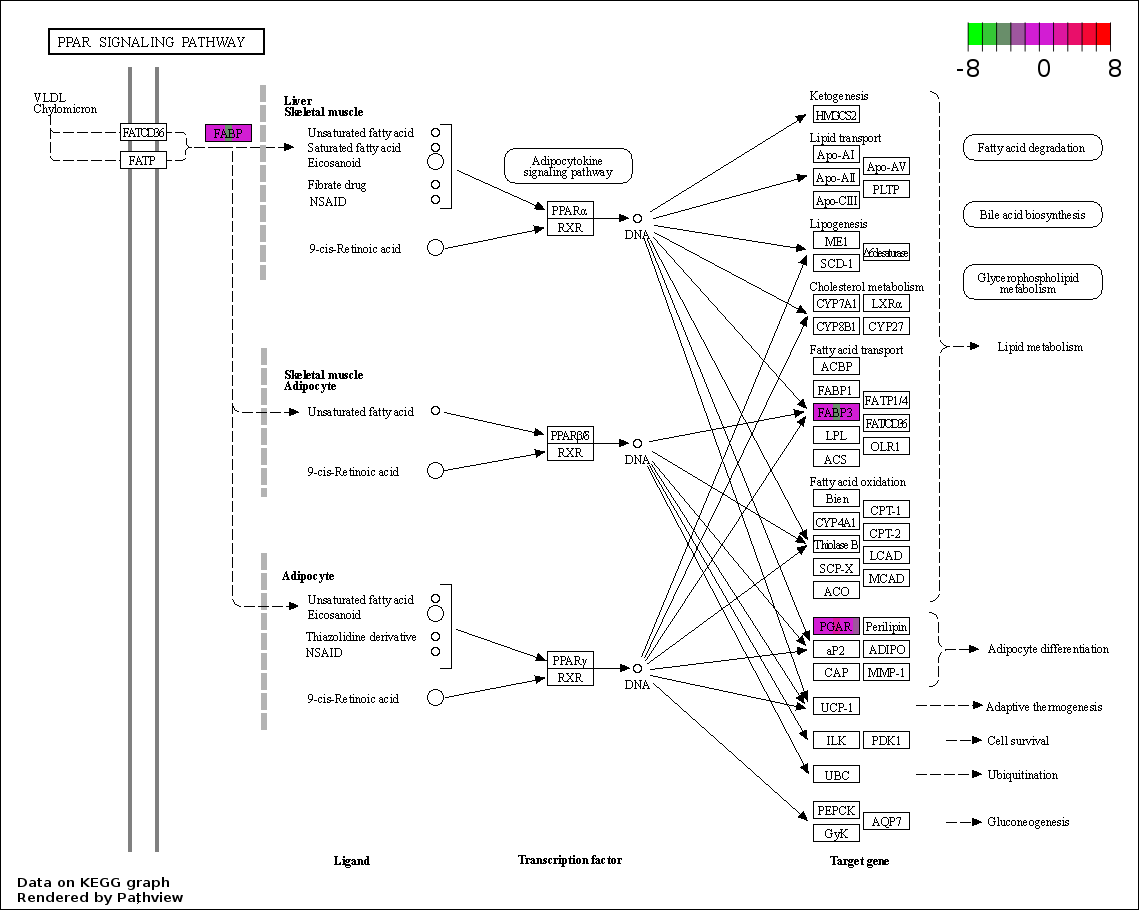

Supplement: Supplementary file 19 — Supplementary file19 (PNG 28 KB) [file 441_2024_3933_MOESM19_ESM.png]

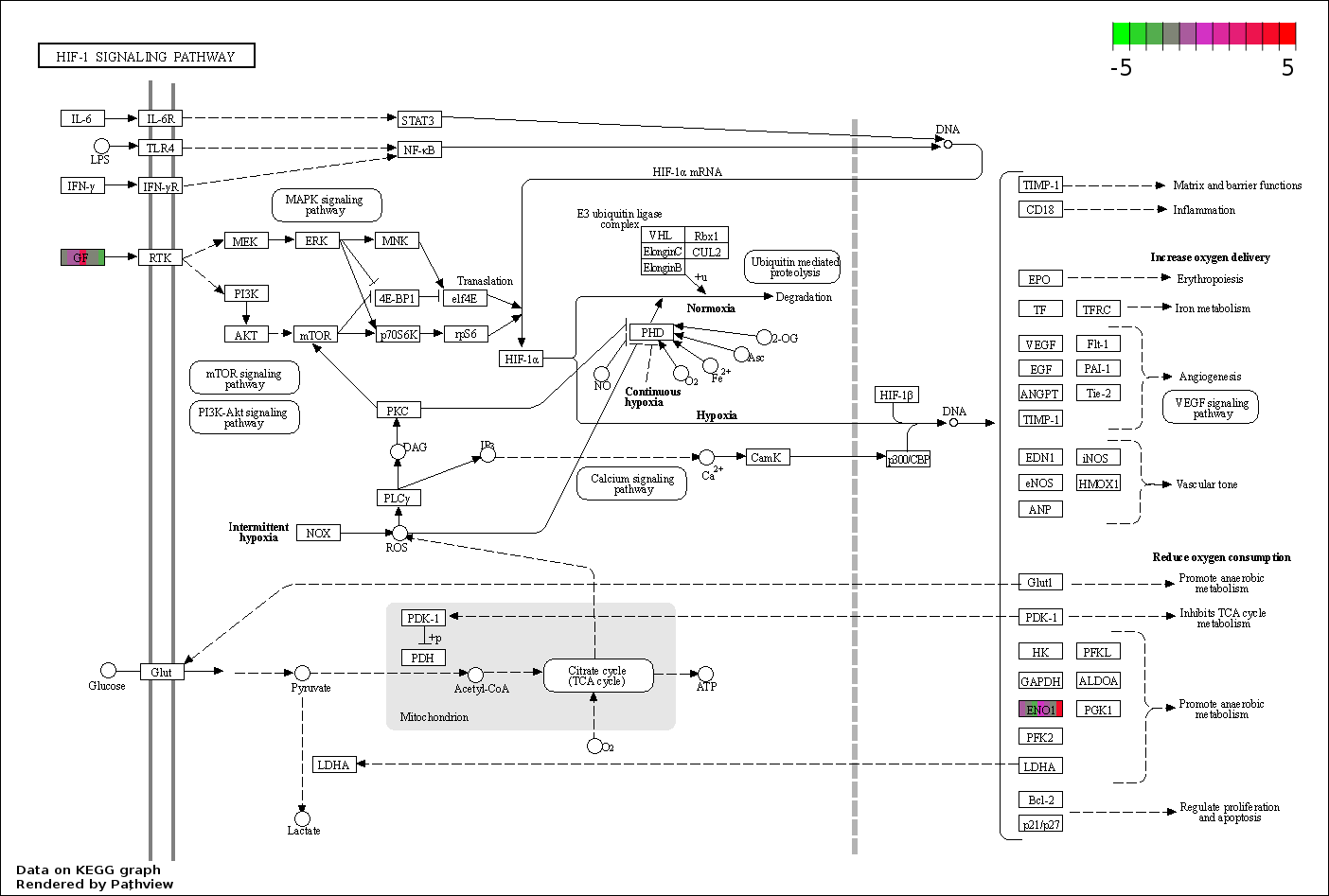

Supplement: Supplementary file 20 — Supplementary file20 (PNG 29 KB) [file 441_2024_3933_MOESM20_ESM.png]

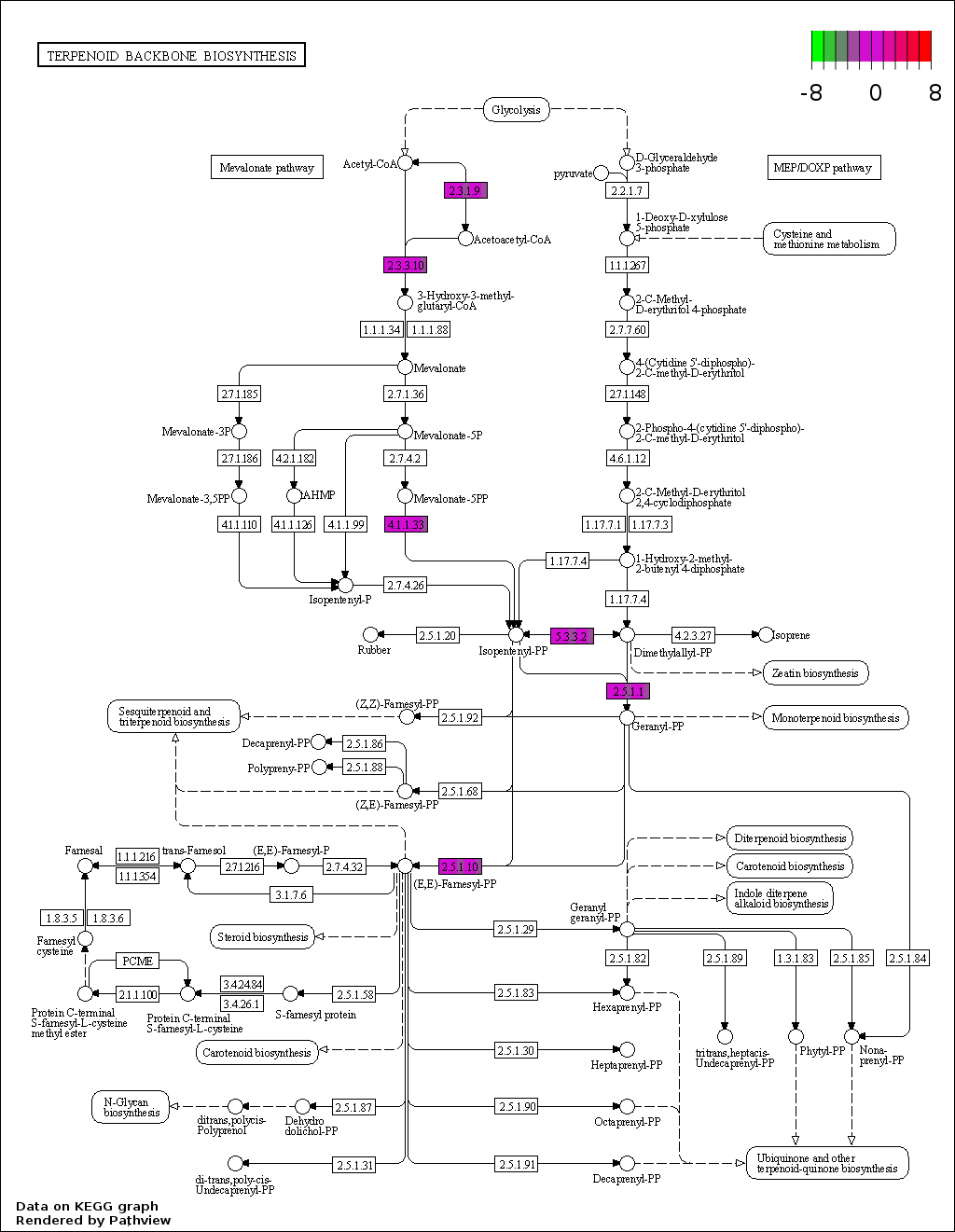

Supplement: Supplementary file 21 — Supplementary file21 (PNG 33 KB) [file 441_2024_3933_MOESM21_ESM.png]

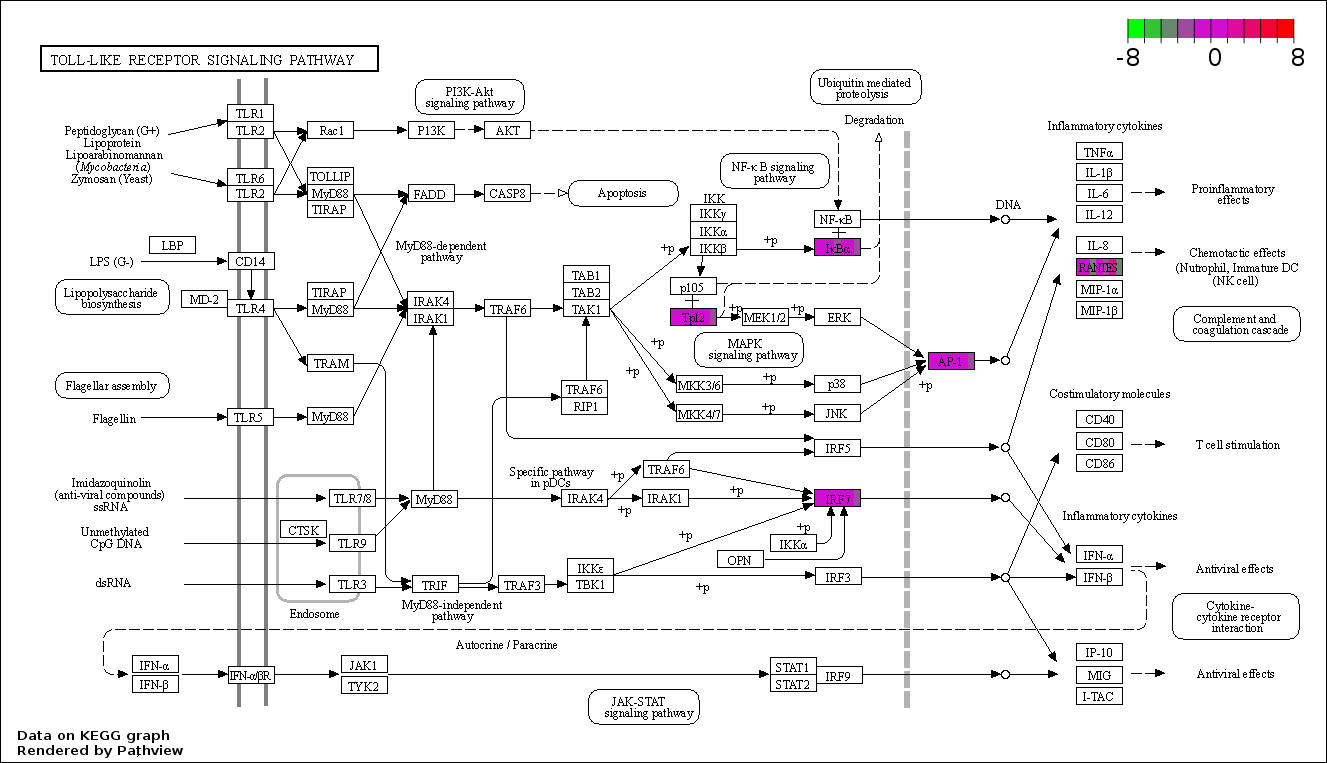

Supplement: Supplementary file 22 — Supplementary file22 (PNG 30 KB) [file 441_2024_3933_MOESM22_ESM.png]

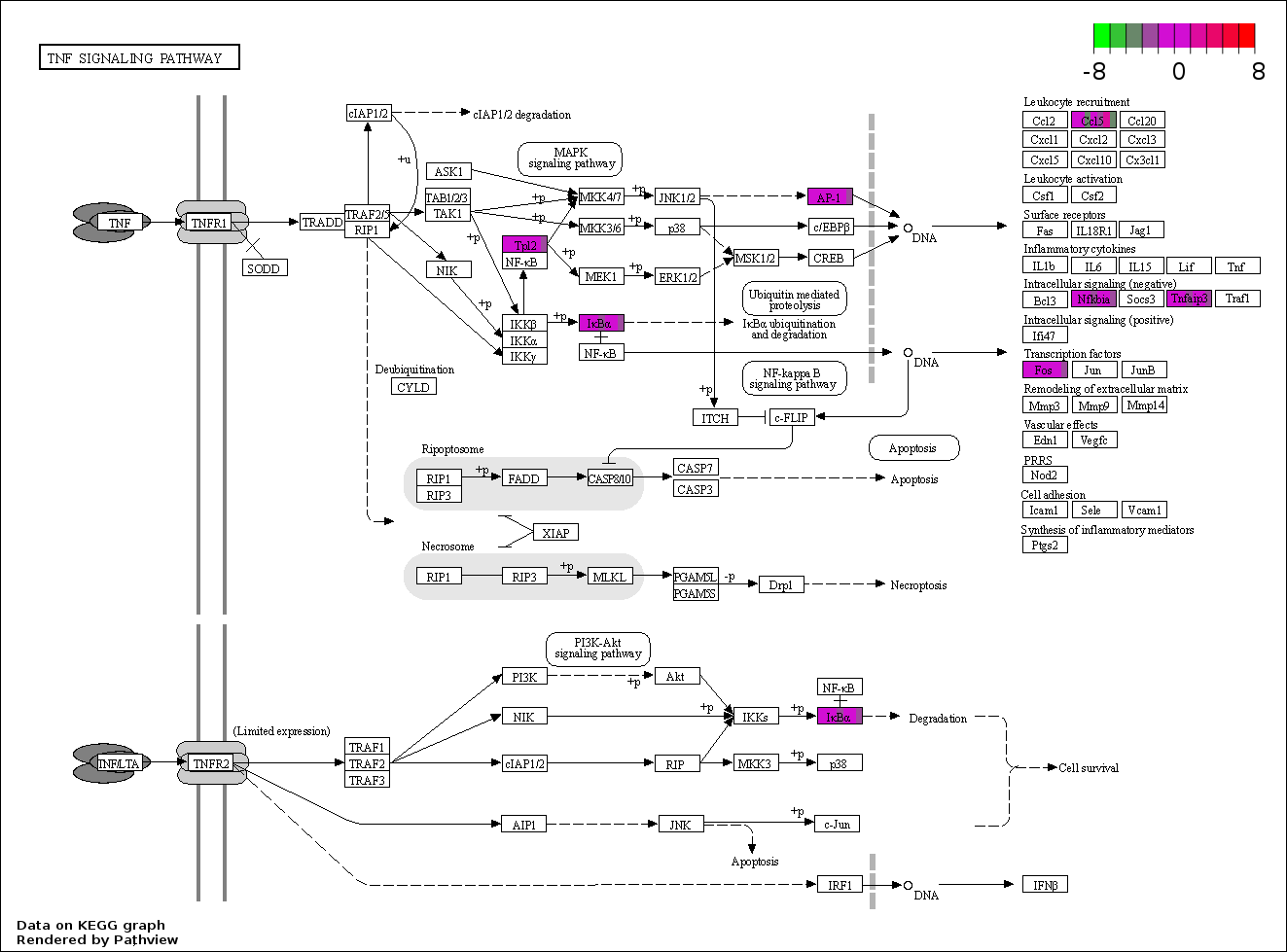

Supplement: Supplementary file 23 — Supplementary file23 (PNG 41 KB) [file 441_2024_3933_MOESM23_ESM.png]

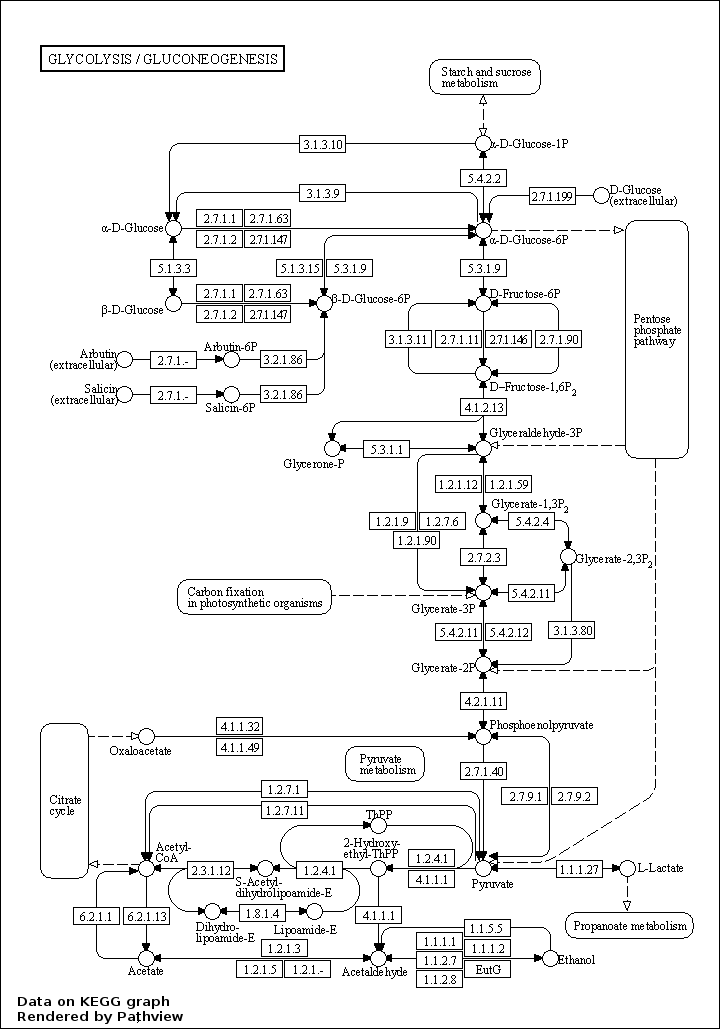

Supplement: Supplementary file 24 — Supplementary file24 (PNG 21 KB) [file 441_2024_3933_MOESM24_ESM.png]

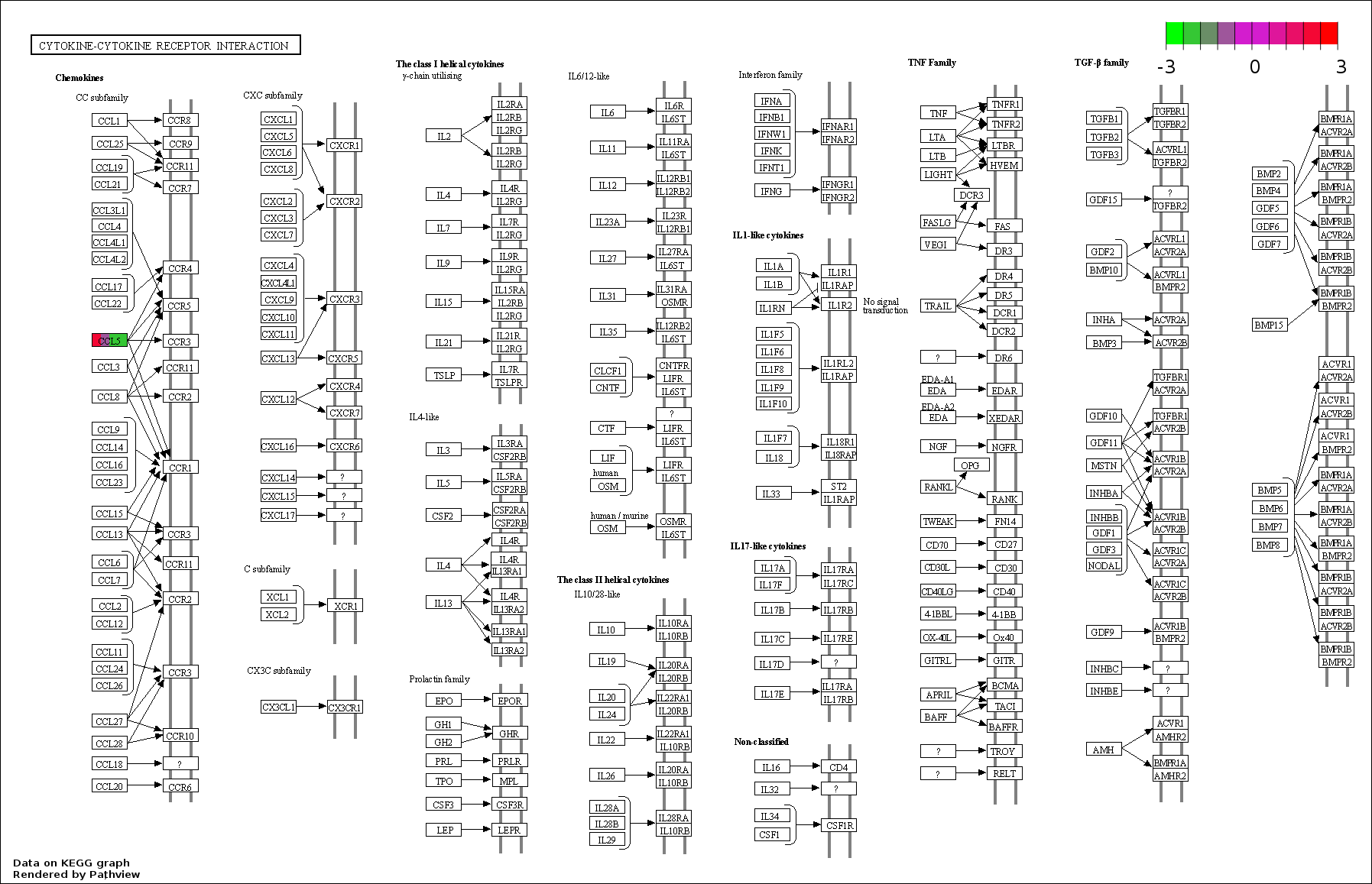

Supplement: Supplementary file 25 — Supplementary file25 (PNG 63 KB) [file 441_2024_3933_MOESM25_ESM.png]

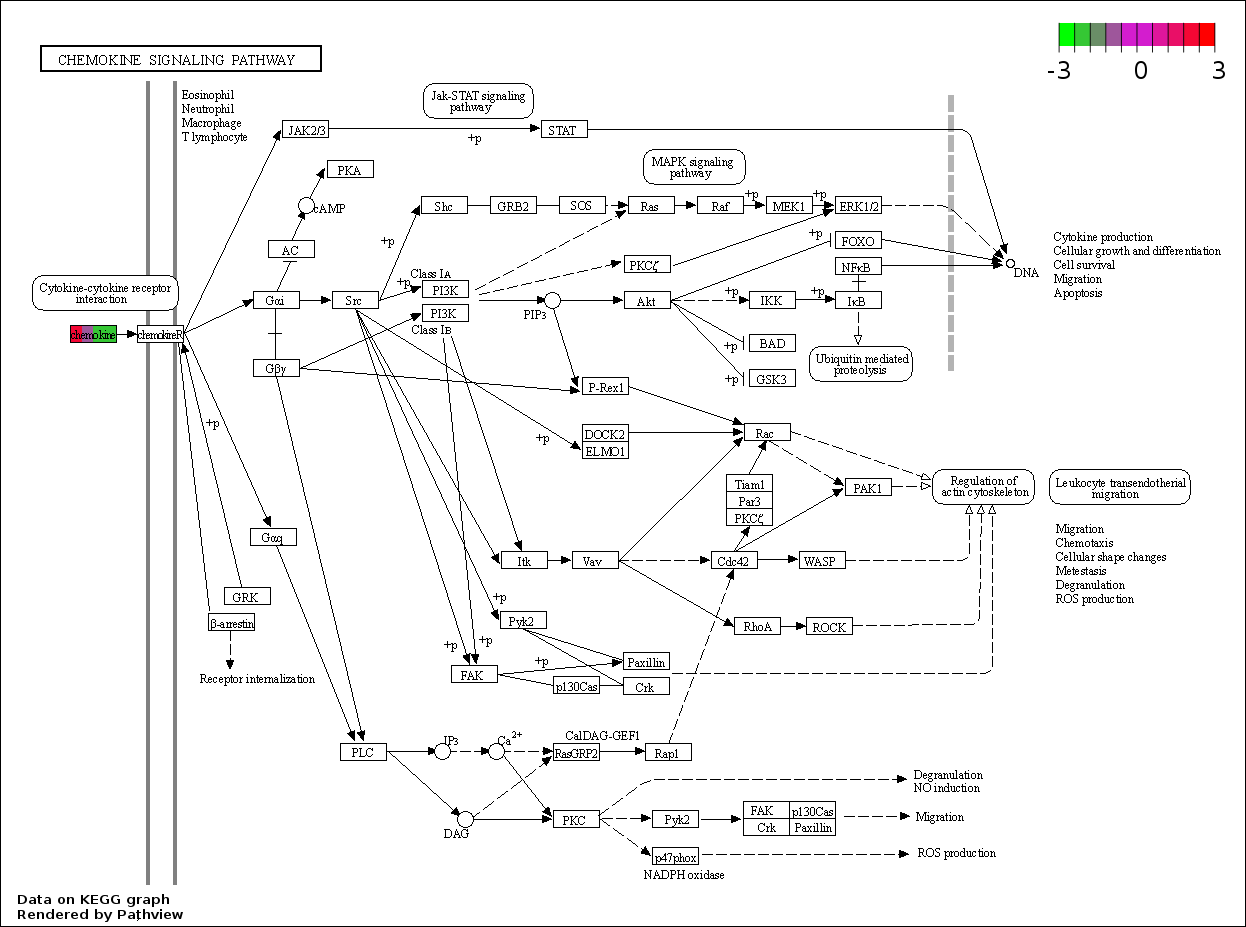

Supplement: Supplementary file 26 — Supplementary file26 (PNG 38 KB) [file 441_2024_3933_MOESM26_ESM.png]

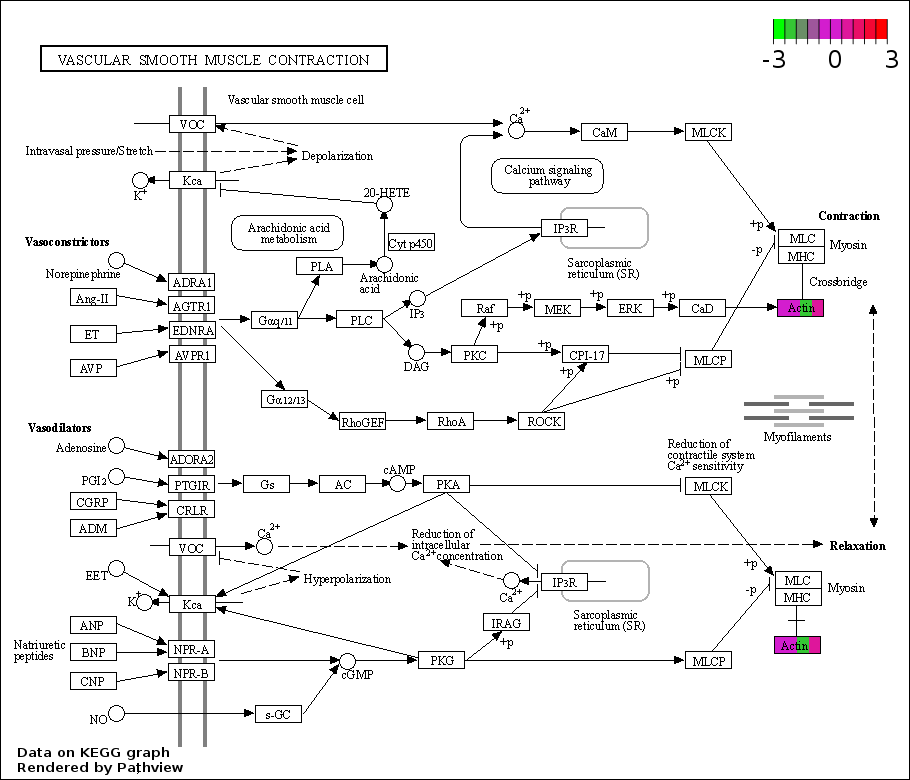

Supplement: Supplementary file 27 — Supplementary file27 (PNG 30 KB) [file 441_2024_3933_MOESM27_ESM.png]
