# Supplementary material for: Single cell transcriptomics profiling of the stromal cells in the pathologic association of ribosomal proteins in the ischemic myocardium and epicardial fat
Source: Cell Tissue Res. 2024 Dec 6;399(2):173–92. doi: 10.1007/s00441-024-03933-3 (PMC11787193; doi:10.1007/s00441-024-03933-3)
Supplement: Supplementary file 28 — Supplementary file28 (DOCX 71 KB) [file 441_2024_3933_MOESM28_ESM.docx]

**Supplementary Table 1**: Altered genes in the subpopulations of LVSCs and EATDS based on scRNA-seq analysis.

| **LVSC RPS18** | | | | |  |  |  |  |
| --- | --- | --- | --- | --- | --- | --- | --- | --- |
| **Cluster 1** | | | | |  |  |  |  |
| **Upregulated** | | | | |  |  |  |  |
| **SI/No** | **Feature ID** | **Genes** | **FC** | **P-Value** |  |  |  |  |
| 1 | ENSSSCG00000032795 | IL1RL1 | 5.4 | 8.93E-69 |  |  |  |  |
| 2 | ENSSSCG00000029326 | CCNB1 | 5.3 | 3.32E-91 |  |  |  |  |
| 3 | ENSSSCG00000017473 | TOP2A | 5.25 | 1.83E-90 |  |  |  |  |
| 4 | ENSSSCG00000017904 | ENO3 | 5.08 | 4.40E-83 |  |  |  |  |
| 5 | ENSSSCG00000004554 | PCLAF | 4.95 | 1.34E-91 |  |  |  |  |
| 6 | ENSSSCG00000009671 | PBK | 4.9 | 6.10E-80 |  |  |  |  |
| 7 | ENSSSCG00000005056 | DLGAP5 | 4.79 | 2.51E-79 |  |  |  |  |
| 8 | ENSSSCG00000038929 | CEMIP | 4.79 | 3.23E-63 |  |  |  |  |
| 9 | ENSSSCG00000017022 | HMMR | 4.73 | 2.26E-76 |  |  |  |  |
| 10 | ENSSSCG00000007423 | UBE2C | 4.73 | 6.33E-76 |  |  |  |  |
| 11 | ENSSSCG00000026748 | PLK1 | 4.68 | 1.07E-67 |  |  |  |  |
| 12 | ENSSSCG00000031741 | ENSSSCG00000031741 | 4.65 | 3.62E-72 |  |  |  |  |
| 13 | ENSSSCG00000015581 | CENPF | 4.58 | 5.63E-72 |  |  |  |  |
| 14 | ENSSSCG00000010471 | KIF11 | 4.36 | 1.93E-60 |  |  |  |  |
| 15 | ENSSSCG00000023296 | CENPE | 4.27 | 2.26E-64 |  |  |  |  |
| 16 | ENSSSCG00000009448 | DIAPH3 | 4.24 | 2.32E-67 |  |  |  |  |
| 17 | ENSSSCG00000004969 | KIF23 | 4.05 | 6.72E-61 |  |  |  |  |
| 18 | ENSSSCG00000037120 | TK1 | 4.03 | 6.97E-57 |  |  |  |  |
| 19 | ENSSSCG00000003949 | CDC20 | 4.03 | 1.79E-52 |  |  |  |  |
| 20 | ENSSSCG00000007235 | TPX2 | 4.01 | 5.65E-60 |  |  |  |  |
| 21 | ENSSSCG00000010190 | ENSSSCG00000010190 | 4 | 5.29E-34 |  |  |  |  |
| 22 | ENSSSCG00000007493 | ENSSSCG00000007493 | 3.92 | 7.54E-52 |  |  |  |  |
| 23 | ENSSSCG00000040486 | BIRC5 | 3.84 | 1.73E-55 |  |  |  |  |
| 24 | ENSSSCG00000007366 | MYBL2 | 3.84 | 1.06E-56 |  |  |  |  |
| 25 | ENSSSCG00000016092 | SGO2 | 3.8 | 5.64E-49 |  |  |  |  |
| 26 | ENSSSCG00000000217 | RACGAP1 | 3.52 | 6.49E-47 |  |  |  |  |
| 27 | ENSSSCG00000008747 | NCAPG | 3.51 | 2.24E-44 |  |  |  |  |
| 28 | ENSSSCG00000029005 | KPNA2 | 3.47 | 5.14E-51 |  |  |  |  |
| 29 | ENSSSCG00000017032 | ENSSSCG00000017032 | 3.46 | 6.78E-48 |  |  |  |  |
| 30 | ENSSSCG00000006333 | NUF2 | 3.28 | 6.84E-42 |  |  |  |  |
| 31 | ENSSSCG00000016658 | ANLN | 3.25 | 1.26E-45 |  |  |  |  |
| 32 | ENSSSCG00000010457 | KIF20B | 3.15 | 2.81E-38 |  |  |  |  |
| 33 | ENSSSCG00000035544 | ENSSSCG00000035544 | 3.12 | 1.33E-40 |  |  |  |  |
| 34 | ENSSSCG00000021161 | CKS2 | 3.11 | 7.34E-42 |  |  |  |  |
| 35 | ENSSSCG00000020785 | DES | 2.97 | 2.22E-37 |  |  |  |  |
| 36 | ENSSSCG00000006474 | NES | 2.75 | 1.70E-29 |  |  |  |  |
| 37 | ENSSSCG00000005983 | ATAD2 | 2.74 | 7.27E-29 |  |  |  |  |
| 38 | ENSSSCG00000032996 | SLC7A5 | 2.73 | 3.74E-30 |  |  |  |  |
| 39 | ENSSSCG00000010923 | UBE2T | 2.66 | 2.20E-29 |  |  |  |  |
| 40 | ENSSSCG00000010461 | ANKRD1 | 2.65 | 2.95E-30 |  |  |  |  |
| 41 | ENSSSCG00000009093 | BBS7 | 2.62 | 3.74E-28 |  |  |  |  |
| 42 | ENSSSCG00000008729 | LYAR | 2.56 | 1.28E-25 |  |  |  |  |
| 43 | ENSSSCG00000005047 | CDKN3 | 2.54 | 3.02E-27 |  |  |  |  |
| 44 | ENSSSCG00000033444 | SPC24 | 2.53 | 1.46E-28 |  |  |  |  |
| 45 | ENSSSCG00000009178 | H2AFZ | 2.47 | 3.07E-28 |  |  |  |  |
| 46 | ENSSSCG00000009704 | HMGB2 | 2.42 | 1.37E-25 |  |  |  |  |
| 47 | ENSSSCG00000010568 | NPM3 | 2.41 | 1.01E-24 |  |  |  |  |
| 48 | ENSSSCG00000035746 | ECT2 | 2.29 | 1.90E-21 |  |  |  |  |
| 49 | ENSSSCG00000011731 | SMC4 | 2.26 | 2.56E-22 |  |  |  |  |
| 50 | ENSSSCG00000032094 | DKK2 | 2.25 | 3.64E-19 |  |  |  |  |
| 51 | ENSSSCG00000007043 | GPCPD1 | 2.24 | 1.62E-20 |  |  |  |  |
| 52 | ENSSSCG00000008289 | MTHFD2 | 2.2 | 3.41E-21 |  |  |  |  |
| 53 | ENSSSCG00000037016 | ID1 | 2.19 | 6.20E-21 |  |  |  |  |
| 54 | ENSSSCG00000003789 | CTH | 2.19 | 2.59E-20 |  |  |  |  |
| 55 | ENSSSCG00000035908 | ENSSSCG00000035908 | 2.1 | 1.44E-18 |  |  |  |  |
| 56 | ENSSSCG00000028210 | NT5C | 2.09 | 5.06E-18 |  |  |  |  |
| 57 | ENSSSCG00000036256 | MAD2L1 | 2.09 | 2.46E-18 |  |  |  |  |
| 58 | ENSSSCG00000006051 | CTHRC1 | 2.04 | 1.45E-17 |  |  |  |  |
| 59 | ENSSSCG00000021966 | TOPBP1 | 2.04 | 1.92E-17 |  |  |  |  |
| 60 | ENSSSCG00000005287 | PSAT1 | 2.01 | 2.29E-18 |  |  |  |  |
| 61 | ENSSSCG00000004139 | ADGRG6 | 2.01 | 8.21E-18 |  |  |  |  |
| **Downregulated** | | | | |  |  |  |  |
| **SI/No** | **Feature ID** | **Genes** | **FC** | **P-Value** |  |  |  |  |
| 1 | ENSSSCG00000017723 | CCL2 | -2.03 | 9.70E-12 |  |  |  |  |
| 2 | ENSSSCG00000011928 | CCDC80 | -2.09 | 1.45E-12 |  |  |  |  |
| 3 | ENSSSCG00000008963 | AREG | -2.09 | 1.30E-07 |  |  |  |  |
| 4 | ENSSSCG00000001565 | CDKN1A | -2.13 | 2.76E-12 |  |  |  |  |
| 5 | ENSSSCG00000004241 | GJA1 | -2.19 | 1.55E-12 |  |  |  |  |
| 6 | ENSSSCG00000023784 | SEMA3C | -2.2 | 3.53E-13 |  |  |  |  |
| 7 | ENSSSCG00000000916 | LUM | -2.25 | 4.70E-14 |  |  |  |  |
| 8 | ENSSSCG00000005055 | LGALS3 | -2.38 | 6.15E-15 |  |  |  |  |
| 9 | ENSSSCG00000038912 | IFITM3 | -2.51 | 9.89E-17 |  |  |  |  |
| 10 | ENSSSCG00000004651 | GALK2 | -2.66 | 4.72E-17 |  |  |  |  |
| 11 | ENSSSCG00000024388 | BNIP3 | -2.68 | 6.73E-19 |  |  |  |  |
| 12 | ENSSSCG00000032436 | ENSSSCG00000032436 | -3.15 | 1.93E-23 |  |  |  |  |
| 13 | ENSSSCG00000037697 | MGP | -3.53 | 1.40E-23 |  |  |  |  |
| 14 | ENSSSCG00000035392 | IGFBP2 | -3.59 | 1.55E-27 |  |  |  |  |
| 15 | ENSSSCG00000034570 | ENSSSCG00000034570 | -4.16 | 2.21E-35 |  |  |  |  |
| **Cluster 2** | | | | |  |  |  |  |
| **Downregulated** | | | | |  |  |  |  |
| **SI/No** | **Feature ID** | **Genes** | **ISC/R FC** | **P-Value** |  |  |  |  |
| 1 | ENSSSCG00000009004 | SFRP2 | -2.24 | 7.72E-11 |  |  |  |  |
| 2 | ENSSSCG00000020785 | DES | -2.25 | 1.14E-11 |  |  |  |  |
| **Cluster 3** | | | | |  |  |  |  |
| **Upregulated** | | | | |  |  |  |  |
| **SI/No** | **Feature ID** | **Genes** | **ISC/R FC** | **P-Value** |  |  |  |  |
| 1 | ENSSSCG00000017705 | CCL5 | 2.49 | 7.47E-03 |  |  |  |  |
| 2 | ENSSSCG00000035392 | IGFBP2 | 2.23 | 2.03E-23 |  |  |  |  |
| 3 | ENSSSCG00000037697 | MGP | 2.18 | 1.35E-19 |  |  |  |  |
| **Downregulated** | | | | |  |  |  |  |
| **SI/No** | **Feature ID** | **Genes** | **ISC/R FC** | **P-Value** |  |  |  |  |
| 1 | ENSSSCG00000008963 | AREG | -2.28 | 1.87E-11 |  |  |  |  |
| **Cluster 4** | | | | |  |  |  |  |
| **Upregulated** | | | | |  |  |  |  |
| **SI/No** | **Feature ID** | **Genes** | **ISC FC** | **P-Value** |  |  |  |  |
| 1 | ENSSSCG00000012027 | ADAMTS5 | 5.18 | 1.50E-49 |  |  |  |  |
| 2 | ENSSSCG00000025924 | IGFBP5 | 4.93 | 1.75E-54 |  |  |  |  |
| 3 | ENSSSCG00000000857 | IGF1 | 4.09 | 3.26E-62 |  |  |  |  |
| 4 | ENSSSCG00000017380 | ARL4D | 4.02 | 1.50E-49 |  |  |  |  |
| 5 | ENSSSCG00000008963 | AREG | 3.91 | 3.75E-45 |  |  |  |  |
| 6 | ENSSSCG00000031661 | ENSSSCG00000031661 | 3.34 | 4.89E-42 |  |  |  |  |
| 7 | ENSSSCG00000031866 | TIMP3 | 3.01 | 6.08E-28 |  |  |  |  |
| 8 | ENSSSCG00000011147 | AKR1C2 | 2.96 | 7.95E-16 |  |  |  |  |
| 9 | ENSSSCG00000010948 | CTSL | 2.69 | 1.23E-10 |  |  |  |  |
| 10 | ENSSSCG00000004241 | GJA1 | 2.59 | 5.00E-29 |  |  |  |  |
| 11 | ENSSSCG00000009004 | SFRP2 | 2.59 | 1.26E-25 |  |  |  |  |
| 12 | ENSSSCG00000004651 | GALK2 | 2.54 | 1.14E-27 |  |  |  |  |
| 13 | ENSSSCG00000024791 | ENSSSCG00000024791 | 2.53 | 1.75E-26 |  |  |  |  |
| 14 | ENSSSCG00000005608 | ANGPTL2 | 2.48 | 2.55E-26 |  |  |  |  |
| 15 | ENSSSCG00000015271 | PRELP | 2.47 | 9.79E-27 |  |  |  |  |
| 16 | ENSSSCG00000014924 | CTSC | 2.41 | 2.95E-22 |  |  |  |  |
| 17 | ENSSSCG00000013599 | ANGPTL4 | 2.39 | 1.32E-19 |  |  |  |  |
| 18 | ENSSSCG00000024043 | ADAMTS2 | 2.26 | 1.69E-21 |  |  |  |  |
| 19 | ENSSSCG00000016718 | NPY | 2.26 | 2.39E-13 |  |  |  |  |
| 20 | ENSSSCG00000001873 | CSPG4 | 2.24 | 2.87E-21 |  |  |  |  |
| 21 | ENSSSCG00000036136 | BHLHE40 | 2.22 | 2.36E-21 |  |  |  |  |
| 22 | ENSSSCG00000001565 | CDKN1A | 2.21 | 1.45E-21 |  |  |  |  |
| 23 | ENSSSCG00000018081 | ATP6 | 2.18 | 1.70E-21 |  |  |  |  |
| 24 | ENSSSCG00000005055 | LGALS3 | 2.15 | 2.00E-20 |  |  |  |  |
| 25 | ENSSSCG00000035495 | KITLG | 2.13 | 9.52E-19 |  |  |  |  |
| 26 | ENSSSCG00000000010 | FBLN1 | 2.11 | 4.56E-16 |  |  |  |  |
| 27 | ENSSSCG00000004484 | COL12A1 | 2.09 | 5.38E-19 |  |  |  |  |
| 28 | ENSSSCG00000005423 | ABCA1 | 2.04 | 1.18E-16 |  |  |  |  |
| 29 | ENSSSCG00000011521 | PDZRN3 | 2.03 | 1.21E-17 |  |  |  |  |
| 30 | ENSSSCG00000022162 | RAB11FIP5 | 2.01 | 4.88E-17 |  |  |  |  |
| **Downregulated** | | | | |  |  |  |  |
| **SI/No** | **Feature ID** | **Genes** | **ISC FC** | **P-Value** |  |  |  |  |
|  | ENSSSCG00000029066 | IDI1 | -2.00 | 1.33E-10 |  |  |  |  |
|  | ENSSSCG00000008294 | ACTG2 | -2.06 | 1.05E-11 |  |  |  |  |
|  | ENSSSCG00000032436 | ENSSSCG00000032436 | -2.18 | 8.65E-13 |  |  |  |  |
|  | ENSSSCG00000035392 | IGFBP2 | -2.21 | 1.47E-12 |  |  |  |  |
|  | ENSSSCG00000038912 | IFITM3 | -2.32 | 1.79E-14 |  |  |  |  |
|  | ENSSSCG00000033727 | GPX1 | -2.57 | 2.33E-17 |  |  |  |  |
|  | ENSSSCG00000035297 | ISG12(A) | -2.67 | 1.33E-17 |  |  |  |  |
|  | ENSSSCG00000037697 | MGP | -2.70 | 2.86E-15 |  |  |  |  |
|  | ENSSSCG00000034570 | ENSSSCG00000034570 | -2.93 | 2.99E-20 |  |  |  |  |
|  | ENSSSCG00000040575 | ISG15 | -3.01 | 4.39E-20 |  |  |  |  |
|  | ENSSSCG00000036114 | RPL22L1 | -3.56 | 5.54E-29 |  |  |  |  |
| **LVSC RPSA** | | | | |  |  |  |  |
| **Cluster 1** | | | | |  |  |  |  |
| **Downregulated** | | | | |  |  |  |  |
| **SI/No** | **Feature ID** | **Gene** | **ISC/R FC** | **P-Value** |  |  |  |  |
| 1 | ENSSSCG00000009004 | SFRP2 | -2.17 | 3.94E-09 |  |  |  |  |
| 2 | ENSSSCG00000020785 | DES | -2.43 | 1.13E-12 |  |  |  |  |
| **Cluster 2** | | | | |  |  |  |  |
| **Upregulated** | | | | |  |  |  |  |
| **SI/No** | **Feature ID** | **Genes** | **ISC/R FC** | **P-Value** |  |  |  |  |
| 1 | ENSSSCG00000037697 | MGP | 2.26 | 1.93E-19 |  |  |  |  |
| 2 | ENSSSCG00000017705 | CCL5 | 2.15 | 7.81E-02 |  |  |  |  |
| 3 | ENSSSCG00000035392 | IGFBP2 | 2.08 | 8.70E-20 |  |  |  |  |
| 4 | ENSSSCG00000004154 | TNFAIP3 | 2.04 | 2.38E-16 |  |  |  |  |
| **Downregulated** | | | | |  |  |  |  |
| **SI/No** | **Feature ID** | **Genes** | **ISC/R FC** | **P-Value** |  |  |  |  |
| 1 | ENSSSCG00000008963 | AREG | -2.22 | 7.73E-10 |  |  |  |  |
| 2 | ENSSSCG00000009004 | SFRP2 | -2.95 | 2.16E-21 |  |  |  |  |
| **Cluster 3** | | | | |  |  |  |  |
| **Upregulated** | | | | |  |  |  |  |
| **SI/No** | **Feature ID** | **Genes** | **C FC** | **P-Value** |  |  |  |  |
| 1 | ENSSSCG00000033815 | SST | 9.44 | 2.30E-29 |  |  |  |  |
| 2 | ENSSSCG00000029326 | CCNB1 | 5.43 | 1.37E-85 |  |  |  |  |
| 3 | ENSSSCG00000002849 | SHCBP1 | 5.42 | 9.32E-67 |  |  |  |  |
| 4 | ENSSSCG00000000683 | CDCA3 | 5.27 | 9.90E-66 |  |  |  |  |
| 5 | ENSSSCG00000017473 | TOP2A | 5.25 | 7.07E-80 |  |  |  |  |
| 6 | ENSSSCG00000032795 | IL1RL1 | 5.22 | 3.38E-56 |  |  |  |  |
| 7 | ENSSSCG00000004554 | PCLAF | 5.16 | 9.46E-90 |  |  |  |  |
| 8 | ENSSSCG00000039216 | CENPN | 5.12 | 2.47E-69 |  |  |  |  |
| 9 | ENSSSCG00000017022 | HMMR | 4.97 | 5.38E-75 |  |  |  |  |
| 10 | ENSSSCG00000009671 | PBK | 4.92 | 7.48E-71 |  |  |  |  |
| 11 | ENSSSCG00000031741 | ENSSSCG00000031741 | 4.90 | 5.93E-68 |  |  |  |  |
| 12 | ENSSSCG00000017904 | ENO3 | 4.86 | 6.16E-70 |  |  |  |  |
| 13 | ENSSSCG00000005056 | DLGAP5 | 4.85 | 9.40E-72 |  |  |  |  |
| 14 | ENSSSCG00000007423 | UBE2C | 4.79 | 2.59E-70 |  |  |  |  |
| 15 | ENSSSCG00000026748 | PLK1 | 4.76 | 1.14E-61 |  |  |  |  |
| 16 | ENSSSCG00000038929 | CEMIP | 4.68 | 1.13E-50 |  |  |  |  |
| 17 | ENSSSCG00000015581 | CENPF | 4.61 | 1.52E-63 |  |  |  |  |
| 18 | ENSSSCG00000010190 | ENSSSCG00000010190 | 4.41 | 3.43E-38 |  |  |  |  |
| 19 | ENSSSCG00000010471 | KIF11 | 4.40 | 2.35E-52 |  |  |  |  |
| 20 | ENSSSCG00000037307 | PRC1 | 4.39 | 7.52E-52 |  |  |  |  |
| 21 | ENSSSCG00000023296 | CENPE | 4.27 | 1.10E-56 |  |  |  |  |
| 22 | ENSSSCG00000004969 | KIF23 | 4.24 | 1.31E-59 |  |  |  |  |
| 23 | ENSSSCG00000009448 | DIAPH3 | 4.15 | 2.58E-58 |  |  |  |  |
| 24 | ENSSSCG00000037120 | TK1 | 4.15 | 2.61E-50 |  |  |  |  |
| 25 | ENSSSCG00000003949 | CDC20 | 4.13 | 2.71E-48 |  |  |  |  |
| 26 | ENSSSCG00000007235 | TPX2 | 4.11 | 9.02E-56 |  |  |  |  |
| 27 | ENSSSCG00000007493 | ENSSSCG00000007493 | 4.01 | 9.92E-48 |  |  |  |  |
| 28 | ENSSSCG00000007366 | MYBL2 | 3.95 | 9.95E-54 |  |  |  |  |
| 29 | ENSSSCG00000040486 | BIRC5 | 3.84 | 6.29E-50 |  |  |  |  |
| 30 | ENSSSCG00000016092 | SGO2 | 3.84 | 4.17E-45 |  |  |  |  |
| 31 | ENSSSCG00000017032 | ENSSSCG00000017032 | 3.64 | 5.35E-48 |  |  |  |  |
| 32 | ENSSSCG00000029005 | KPNA2 | 3.62 | 1.21E-50 |  |  |  |  |
| 33 | ENSSSCG00000000217 | RACGAP1 | 3.62 | 2.34E-44 |  |  |  |  |
| 34 | ENSSSCG00000008747 | NCAPG | 3.54 | 1.85E-39 |  |  |  |  |
| 35 | ENSSSCG00000006333 | NUF2 | 3.43 | 3.07E-41 |  |  |  |  |
| 36 | ENSSSCG00000021161 | CKS2 | 3.28 | 4.81E-43 |  |  |  |  |
| 37 | ENSSSCG00000016658 | ANLN | 3.21 | 3.57E-41 |  |  |  |  |
| 38 | ENSSSCG00000035544 | ENSSSCG00000035544 | 3.12 | 4.95E-37 |  |  |  |  |
| 39 | ENSSSCG00000010457 | KIF20B | 3.09 | 8.43E-33 |  |  |  |  |
| 40 | ENSSSCG00000020785 | DES | 2.91 | 1.06E-32 |  |  |  |  |
| 41 | ENSSSCG00000009093 | BBS7 | 2.80 | 3.97E-29 |  |  |  |  |
| 42 | ENSSSCG00000032996 | SLC7A5 | 2.79 | 1.56E-28 |  |  |  |  |
| 43 | ENSSSCG00000005983 | ATAD2 | 2.76 | 1.78E-26 |  |  |  |  |
| 44 | ENSSSCG00000010923 | UBE2T | 2.75 | 4.14E-29 |  |  |  |  |
| 45 | ENSSSCG00000010461 | ANKRD1 | 2.73 | 4.99E-30 |  |  |  |  |
| 46 | ENSSSCG00000009178 | H2AFZ | 2.64 | 2.49E-30 |  |  |  |  |
| 47 | ENSSSCG00000005047 | CDKN3 | 2.59 | 4.69E-26 |  |  |  |  |
| 48 | ENSSSCG00000006474 | NES | 2.59 | 1.46E-23 |  |  |  |  |
| 49 | ENSSSCG00000008729 | LYAR | 2.56 | 1.27E-23 |  |  |  |  |
| 50 | ENSSSCG00000010568 | NPM3 | 2.55 | 8.76E-26 |  |  |  |  |
| 51 | ENSSSCG00000016600 | TMEM229A | 2.53 | 8.15E-23 |  |  |  |  |
| 52 | ENSSSCG00000033444 | SPC24 | 2.46 | 5.50E-25 |  |  |  |  |
| 53 | ENSSSCG00000009704 | HMGB2 | 2.45 | 2.70E-24 |  |  |  |  |
| 54 | ENSSSCG00000007043 | GPCPD1 | 2.38 | 5.10E-21 |  |  |  |  |
| 55 | ENSSSCG00000035746 | ECT2 | 2.38 | 3.99E-21 |  |  |  |  |
| 56 | ENSSSCG00000011731 | SMC4 | 2.35 | 1.97E-22 |  |  |  |  |
| 57 | ENSSSCG00000003789 | CTH | 2.28 | 2.80E-20 |  |  |  |  |
| 58 | ENSSSCG00000008289 | MTHFD2 | 2.25 | 1.03E-20 |  |  |  |  |
| 59 | ENSSSCG00000037016 | ID1 | 2.23 | 5.69E-20 |  |  |  |  |
| 60 | ENSSSCG00000035908 | ENSSSCG00000035908 | 2.22 | 2.13E-19 |  |  |  |  |
| 61 | ENSSSCG00000028210 | NT5C | 2.18 | 7.41E-18 |  |  |  |  |
| 62 | ENSSSCG00000003583 | RPA2 | 2.17 | 7.76E-18 |  |  |  |  |
| 63 | ENSSSCG00000032094 | DKK2 | 2.14 | 9.65E-16 |  |  |  |  |
| 64 | ENSSSCG00000036256 | MAD2L1 | 2.13 | 5.81E-18 |  |  |  |  |
| 65 | ENSSSCG00000011538 | LMCD1 | 2.11 | 9.78E-15 |  |  |  |  |
| 66 | ENSSSCG00000004139 | ADGRG6 | 2.11 | 2.23E-18 |  |  |  |  |
| 67 | ENSSSCG00000015340 | ASNS | 2.05 | 7.06E-18 |  |  |  |  |
| 68 | ENSSSCG00000021966 | TOPBP1 | 2.05 | 2.60E-16 |  |  |  |  |
| 69 | ENSSSCG00000006051 | CTHRC1 | 2.02 | 5.36E-16 |  |  |  |  |
| 70 | ENSSSCG00000005287 | PSAT1 | 2.01 | 2.37E-17 |  |  |  |  |
| 71 | ENSSSCG00000009531 | TEX30 | 2.00 | 5.49E-15 |  |  |  |  |
| 72 | ENSSSCG00000028019 | LRRC59 | 2.00 | 2.03E-16 |  |  |  |  |
| **Downregulated** | | | | |  |  |  |  |
| **SI/No** | **Feature ID** | **Gene** | **C FC** | **P-Value** |  |  |  |  |
| 1 | ENSSSCG00000017723 | CCL2 | -2.01 | 1.11E-10 |  |  |  |  |
| 2 | ENSSSCG00000000033 | TSPO | -2.10 | 1.13E-11 |  |  |  |  |
| 3 | ENSSSCG00000005055 | LGALS3 | -2.11 | 2.15E-11 |  |  |  |  |
| 4 | ENSSSCG00000011928 | CCDC80 | -2.12 | 6.54E-12 |  |  |  |  |
| 5 | ENSSSCG00000001565 | CDKN1A | -2.15 | 2.12E-11 |  |  |  |  |
| 6 | ENSSSCG00000008857 | MSMO1 | -2.16 | 2.17E-11 |  |  |  |  |
| 7 | ENSSSCG00000023784 | SEMA3C | -2.16 | 1.38E-11 |  |  |  |  |
| 8 | ENSSSCG00000008963 | AREG | -2.16 | 2.14E-06 |  |  |  |  |
| 9 | ENSSSCG00000004241 | GJA1 | -2.20 | 3.30E-11 |  |  |  |  |
| 10 | ENSSSCG00000000916 | LUM | -2.28 | 2.86E-13 |  |  |  |  |
| 11 | ENSSSCG00000004651 | GALK2 | -2.52 | 9.37E-14 |  |  |  |  |
| 12 | ENSSSCG00000038912 | IFITM3 | -2.59 | 2.66E-16 |  |  |  |  |
| 13 | ENSSSCG00000024388 | BNIP3 | -2.78 | 1.79E-18 |  |  |  |  |
| 14 | ENSSSCG00000032436 | ENSSSCG00000032436 | -3.13 | 1.70E-20 |  |  |  |  |
| 15 | ENSSSCG00000037697 | MGP | -3.47 | 4.49E-19 |  |  |  |  |
| 16 | ENSSSCG00000035392 | IGFBP2 | -3.74 | 1.25E-26 |  |  |  |  |
| 17 | ENSSSCG00000034570 | ENSSSCG00000034570 | -4.29 | 8.86E-33 |  |  |  |  |
| **Cluster 4** | | | | |  |  |  |  |
| **Upregulated** | | | | |  |  |  |  |
| **SI/No** | **Feature ID** | **Genes** | **ISC FC** | **P-Value** |  |  |  |  |
| 1 | ENSSSCG00000025924 | IGFBP5 | 5.25 | 3.36E-42 |  |  |  |  |
| 2 | ENSSSCG00000012027 | ADAMTS5 | 5.10 | 1.04E-38 |  |  |  |  |
| 3 | ENSSSCG00000000857 | IGF1 | 4.01 | 5.63E-54 |  |  |  |  |
| 4 | ENSSSCG00000017380 | ARL4D | 3.94 | 7.27E-42 |  |  |  |  |
| 5 | ENSSSCG00000008963 | AREG | 3.90 | 1.03E-37 |  |  |  |  |
| 6 | ENSSSCG00000031661 | ENSSSCG00000031661 | 3.21 | 1.65E-35 |  |  |  |  |
| 7 | ENSSSCG00000031866 | TIMP3 | 2.87 | 1.35E-25 |  |  |  |  |
| 8 | ENSSSCG00000011147 | AKR1C2 | 2.76 | 8.73E-13 |  |  |  |  |
| 9 | ENSSSCG00000009004 | SFRP2 | 2.73 | 1.75E-24 |  |  |  |  |
| 10 | ENSSSCG00000004241 | GJA1 | 2.58 | 1.14E-26 |  |  |  |  |
| 11 | ENSSSCG00000015271 | PRELP | 2.50 | 2.94E-25 |  |  |  |  |
| 12 | ENSSSCG00000005608 | ANGPTL2 | 2.49 | 1.75E-24 |  |  |  |  |
| 13 | ENSSSCG00000024791 | ENSSSCG00000024791 | 2.46 | 3.07E-23 |  |  |  |  |
| 14 | ENSSSCG00000004651 | GALK2 | 2.39 | 8.23E-23 |  |  |  |  |
| 15 | ENSSSCG00000001873 | CSPG4 | 2.37 | 6.36E-22 |  |  |  |  |
| 16 | ENSSSCG00000024043 | ADAMTS2 | 2.30 | 1.29E-20 |  |  |  |  |
| 17 | ENSSSCG00000018081 | ATP6 | 2.25 | 1.48E-21 |  |  |  |  |
| 18 | ENSSSCG00000036136 | BHLHE40 | 2.17 | 4.64E-19 |  |  |  |  |
| 19 | ENSSSCG00000001565 | CDKN1A | 2.16 | 2.50E-19 |  |  |  |  |
| 20 | ENSSSCG00000014924 | CTSC | 2.15 | 9.14E-17 |  |  |  |  |
| 21 | ENSSSCG00000004484 | COL12A1 | 2.09 | 1.17E-17 |  |  |  |  |
| 22 | ENSSSCG00000011521 | PDZRN3 | 2.06 | 9.14E-17 |  |  |  |  |
| 23 | ENSSSCG00000035495 | KITLG | 2.06 | 3.19E-16 |  |  |  |  |
| 24 | ENSSSCG00000011609 | FBLN2 | 2.03 | 2.87E-16 |  |  |  |  |
| 25 | ENSSSCG00000005423 | ABCA1 | 2.03 | 3.83E-15 |  |  |  |  |
| **Downregulated** | | | | |  |  |  |  |
| **SI/No** | **Feature ID** | **Genes** | **ISC FC** | **P-Value** |  |  |  |  |
| 1 | ENSSSCG00000000033 | TSPO | -2.05 | 4.51E-11 |  |  |  |  |
| 2 | ENSSSCG00000035392 | IGFBP2 | -2.10 | 1.57E-10 |  |  |  |  |
| 3 | ENSSSCG00000038912 | IFITM3 | -2.34 | 1.32E-13 |  |  |  |  |
| 4 | ENSSSCG00000032436 | ENSSSCG00000032436 | -2.43 | 6.73E-14 |  |  |  |  |
| 5 | ENSSSCG00000033727 | GPX1 | -2.64 | 3.00E-16 |  |  |  |  |
| 6 | ENSSSCG00000035297 | ISG12(A) | -2.94 | 1.09E-18 |  |  |  |  |
| 7 | ENSSSCG00000034570 | ENSSSCG00000034570 | -3.15 | 9.43E-21 |  |  |  |  |
| 8 | ENSSSCG00000037697 | MGP | -3.21 | 4.06E-17 |  |  |  |  |
| 9 | ENSSSCG00000040575 | ISG15 | -3.47 | 1.25E-22 |  |  |  |  |
| 10 | ENSSSCG00000036114 | RPL22L1 | -3.63 | 6.24E-27 |  |  |  |  |
| **LVSC RPL30** | | | | |  |  |  |  |
| **Cluster 1** | | | | |  |  |  |  |
| **Downregulated** | | | | |  |  |  |  |
| **SI/No** | **Feature ID** | **Genes** | **ISC/R FC** | **P-Value** |  |  |  |  |
| 1 | ENSSSCG00000009004 | SFRP2 | -2.18 | 4.06E-07 |  |  |  |  |
| **Cluster 4** | | | | |  |  |  |  |
| **Upregulated** | | | | |  |  |  |  |
| **SI/No** | **Feature ID** | **Genes** | **ISC FC** | **P-Value** |  |  |  |  |
| 1 | ENSSSCG00000025924 | IGFBP5 | 5.56 | 4.41E-49 |  |  |  |  |
| 2 | ENSSSCG00000012027 | ADAMTS5 | 4.50 | 1.61E-39 |  |  |  |  |
| 3 | ENSSSCG00000017380 | ARL4D | 4.23 | 3.09E-50 |  |  |  |  |
| 4 | ENSSSCG00000008963 | AREG | 3.96 | 5.76E-37 |  |  |  |  |
| 5 | ENSSSCG00000000857 | IGF1 | 3.86 | 1.35E-55 |  |  |  |  |
| 6 | ENSSSCG00000031661 | ENSSSCG00000031661 | 3.21 | 3.37E-38 |  |  |  |  |
| 7 | ENSSSCG00000009004 | SFRP2 | 3.19 | 2.65E-34 |  |  |  |  |
| 8 | ENSSSCG00000031866 | TIMP3 | 3.08 | 3.94E-29 |  |  |  |  |
| 9 | ENSSSCG00000011147 | AKR1C2 | 2.67 | 3.79E-12 |  |  |  |  |
| 10 | ENSSSCG00000015271 | PRELP | 2.66 | 1.25E-30 |  |  |  |  |
| 11 | ENSSSCG00000005608 | ANGPTL2 | 2.60 | 1.02E-28 |  |  |  |  |
| 12 | ENSSSCG00000024791 | ENSSSCG00000024791 | 2.48 | 3.22E-25 |  |  |  |  |
| 13 | ENSSSCG00000001873 | CSPG4 | 2.48 | 1.07E-25 |  |  |  |  |
| 14 | ENSSSCG00000004241 | GJA1 | 2.42 | 6.82E-26 |  |  |  |  |
| 15 | ENSSSCG00000011609 | FBLN2 | 2.41 | 3.49E-24 |  |  |  |  |
| 16 | ENSSSCG00000024043 | ADAMTS2 | 2.36 | 1.91E-23 |  |  |  |  |
| 17 | ENSSSCG00000004651 | GALK2 | 2.35 | 3.49E-24 |  |  |  |  |
| 18 | ENSSSCG00000004484 | COL12A1 | 2.35 | 6.27E-24 |  |  |  |  |
| 19 | ENSSSCG00000000010 | FBLN1 | 2.27 | 1.33E-16 |  |  |  |  |
| 20 | ENSSSCG00000010948 | CTSL | 2.25 | 1.68E-10 |  |  |  |  |
| 21 | ENSSSCG00000018081 | ATP6 | 2.19 | 5.42E-22 |  |  |  |  |
| 22 | ENSSSCG00000014924 | CTSC | 2.13 | 2.62E-18 |  |  |  |  |
| 23 | ENSSSCG00000013599 | ANGPTL4 | 2.06 | 8.78E-14 |  |  |  |  |
| 24 | ENSSSCG00000022162 | RAB11FIP5 | 2.06 | 1.50E-17 |  |  |  |  |
| 25 | ENSSSCG00000028814 | SOD3 | 2.05 | 2.32E-16 |  |  |  |  |
| 26 | ENSSSCG00000007056 | PLCB1 | 2.03 | 5.59E-18 |  |  |  |  |
| 27 | ENSSSCG00000035495 | KITLG | 2.00 | 2.94E-16 |  |  |  |  |
| **Downregulated** | | | | |  |  |  |  |
| **SI/No** | **Feature ID** | **Genes** | **ISC FC** | **P-Value** |  |  |  |  |
| 1 | ENSSSCG00000000033 | TSPO | -2.28 | 6.47E-14 |  |  |  |  |
| 2 | ENSSSCG00000029066 | IDI1 | -2.31 | 5.89E-13 |  |  |  |  |
| 3 | ENSSSCG00000035392 | IGFBP2 | -2.51 | 4.29E-15 |  |  |  |  |
| 4 | ENSSSCG00000032436 | ENSSSCG00000032436 | -2.59 | 1.84E-16 |  |  |  |  |
| 5 | ENSSSCG00000038912 | IFITM3 | -2.65 | 1.56E-17 |  |  |  |  |
| 6 | ENSSSCG00000033727 | GPX1 | -2.69 | 2.58E-17 |  |  |  |  |
| 7 | ENSSSCG00000037697 | MGP | -3.00 | 1.39E-17 |  |  |  |  |
| 8 | ENSSSCG00000035297 | ISG12(A) | -3.02 | 5.62E-21 |  |  |  |  |
| 9 | ENSSSCG00000034570 | ENSSSCG00000034570 | -3.28 | 8.97E-24 |  |  |  |  |
| 10 | ENSSSCG00000036114 | RPL22L1 | -3.58 | 3.52E-27 |  |  |  |  |
| 11 | ENSSSCG00000040575 | ISG15 | -3.59 | 5.90E-24 |  |  |  |  |
| **Cluster 5** | | | | |  |  |  |  |
| **Downregulated** | | | | |  |  |  |  |
| **SI/No** | **Feature ID** | **Genes** | **ISC/R FC** | **P-Value** |  |  |  |  |
| 1 | ENSSSCG00000009004 | SFRP2 | -2.08 | 3.07E-03 |  |  |  |  |
| **Cluster 6** | | | | |  |  |  |  |
| **Downregulated** | | | | |  |  |  |  |
| **SI/No** | **Feature ID** | **Genes** | **ISC/R FC** | **P-Value** |  |  |  |  |
| 1 | ENSSSCG00000009004 | SFRP2 | -2.22 | 1.15E-03 |  |  |  |  |
| **Cluster 7** | | | | | | |  |  |
| **Upregulated** | | | | | | |  |  |
| **SI/No** | **Feature ID** | **Genes** | **C FC** | **P-Value** | **ISC/R FC** | **P-Value** |  |  |
| 1 | ENSSSCG00000032795 | IL1RL1 | 5.69 | 2.70E-37 | -1.41 | 1 |  |  |
| 2 | ENSSSCG00000029326 | CCNB1 | 5.34 | 2.48E-44 | 4.18 | 0.00175 |  |  |
| 3 | ENSSSCG00000000683 | CDCA3 | 5.34 | 4.45E-38 | 4.46 | 0.000769 |  |  |
| 4 | ENSSSCG00000017473 | TOP2A | 5.13 | 4.45E-38 | 4.36 | 0.00114 |  |  |
| 5 | ENSSSCG00000039216 | CENPN | 5.11 | 3.19E-39 | 4.09 | 0.00249 |  |  |
| 6 | ENSSSCG00000002849 | SHCBP1 | 5.07 | 2.94E-35 | 4.37 | 0.00106 |  |  |
| 7 | ENSSSCG00000033443 | DEPDC1 | 5.03 | 1.06E-27 | 4.84 | 0.000336 |  |  |
| 8 | ENSSSCG00000038929 | CEMIP | 5.02 | 5.14E-37 | -0.72 | 1 |  |  |
| 9 | ENSSSCG00000004554 | PCLAF | 4.97 | 6.07E-47 | 3.83 | 0.00282 |  |  |
| 10 | ENSSSCG00000028924 | AURKB | 4.83 | 5.02E-24 | 4.79 | 0.000915 |  |  |
| 11 | ENSSSCG00000011207 | SGO1 | 4.79 | 1.31E-31 | 4.17 | 0.00274 |  |  |
| 12 | ENSSSCG00000007351 | FAM83D | 4.77 | 3.22E-21 | 4.65 | 0.00293 |  |  |
| 13 | ENSSSCG00000007423 | UBE2C | 4.73 | 6.47E-35 | 4.74 | 0.000075 |  |  |
| 14 | ENSSSCG00000017022 | HMMR | 4.71 | 1.20E-36 | 4.15 | 0.00114 |  |  |
| 15 | ENSSSCG00000009671 | PBK | 4.69 | 1.73E-35 | 4.17 | 0.00114 |  |  |
| 16 | ENSSSCG00000010190 | ENSSSCG00000010190 | 4.66 | 4.57E-17 | -3.30 | 1 |  |  |
| 17 | ENSSSCG00000017904 | ENO3 | 4.65 | 1.97E-34 | -1.06 | 1 |  |  |
| 18 | ENSSSCG00000026748 | PLK1 | 4.60 | 1.58E-33 | 3.77 | 0.00995 |  |  |
| 19 | ENSSSCG00000031741 | ENSSSCG00000031741 | 4.42 | 4.41E-31 | 3.42 | 0.0434 |  |  |
| 20 | ENSSSCG00000005056 | DLGAP5 | 4.41 | 6.35E-33 | 4.10 | 0.00114 |  |  |
| 21 | ENSSSCG00000010896 | ASPM | 4.38 | 6.07E-22 | 4.98 | 0.000104 |  |  |
| 22 | ENSSSCG00000015581 | CENPF | 4.34 | 2.06E-26 | 5.10 | 8.78E-06 |  |  |
| 23 | ENSSSCG00000009378 | CKAP2 | 4.21 | 2.18E-17 | 5.25 | 0.000075 |  |  |
| 24 | ENSSSCG00000004969 | KIF23 | 4.10 | 7.19E-34 | 3.55 | 0.00562 |  |  |
| 25 | ENSSSCG00000008125 | NCAPH | 4.04 | 2.77E-15 | 5.22 | 8.42E-05 |  |  |
| 26 | ENSSSCG00000012377 | KIF4A | 4.02 | 2.61E-19 | 4.58 | 0.000639 |  |  |
| 27 | ENSSSCG00000023296 | CENPE | 4.00 | 5.32E-30 | 3.95 | 0.00106 |  |  |
| 28 | ENSSSCG00000037307 | PRC1 | 3.98 | 1.19E-25 | 3.68 | 0.0109 |  |  |
| 29 | ENSSSCG00000003949 | CDC20 | 3.98 | 1.60E-24 | 3.99 | 0.00274 |  |  |
| 30 | ENSSSCG00000003697 | NDC80 | 3.96 | 7.23E-20 | 4.65 | 0.000291 |  |  |
| 31 | ENSSSCG00000009448 | DIAPH3 | 3.94 | 7.66E-33 | 3.25 | 0.0211 |  |  |
| 32 | ENSSSCG00000007235 | TPX2 | 3.94 | 1.50E-28 | 3.99 | 0.00104 |  |  |
| 33 | ENSSSCG00000014248 | LMNB1 | 3.93 | 6.35E-26 | 3.83 | 0.00359 |  |  |
| 34 | ENSSSCG00000010471 | KIF11 | 3.91 | 3.62E-23 | 4.58 | 9.21E-05 |  |  |
| 35 | ENSSSCG00000016092 | SGO2 | 3.75 | 1.77E-25 | 3.43 | 0.0194 |  |  |
| 36 | ENSSSCG00000007493 | ENSSSCG00000007493 | 3.74 | 4.01E-24 | 3.90 | 0.00181 |  |  |
| 37 | ENSSSCG00000037120 | TK1 | 3.69 | 1.73E-23 | 3.42 | 0.0237 |  |  |
| 38 | ENSSSCG00000040486 | BIRC5 | 3.63 | 5.36E-27 | 3.33 | 0.0143 |  |  |
| 39 | ENSSSCG00000007366 | MYBL2 | 3.61 | 6.36E-28 | 2.98 | 0.057 |  |  |
| 40 | ENSSSCG00000028981 | ZNF367 | 3.59 | 4.67E-13 | 5.09 | 8.42E-05 |  |  |
| 41 | ENSSSCG00000017032 | ENSSSCG00000017032 | 3.54 | 5.69E-25 | 3.78 | 0.00143 |  |  |
| 42 | ENSSSCG00000000217 | RACGAP1 | 3.45 | 5.46E-24 | 3.57 | 0.00359 |  |  |
| 43 | ENSSSCG00000026257 | STMN1 | 3.38 | 2.81E-20 | 3.55 | 0.00923 |  |  |
| 44 | ENSSSCG00000029005 | KPNA2 | 3.38 | 3.14E-26 | 2.92 | 0.0399 |  |  |
| 45 | ENSSSCG00000009009 | MND1 | 3.36 | 1.31E-18 | 3.76 | 0.00396 |  |  |
| 46 | ENSSSCG00000008747 | NCAPG | 3.31 | 4.92E-20 | 4.10 | 0.000291 |  |  |
| 47 | ENSSSCG00000029509 | KIF22 | 3.27 | 6.36E-19 | 3.64 | 0.00445 |  |  |
| 48 | ENSSSCG00000021161 | CKS2 | 3.21 | 2.43E-24 | 3.14 | 0.0113 |  |  |
| 49 | ENSSSCG00000006333 | NUF2 | 3.16 | 1.66E-20 | 3.51 | 0.00359 |  |  |
| 50 | ENSSSCG00000016658 | ANLN | 3.05 | 6.21E-22 | 3.14 | 0.0111 |  |  |
| 51 | ENSSSCG00000020785 | DES | 3.01 | 1.45E-19 | -1.85 | 1 |  |  |
| 52 | ENSSSCG00000035544 | ENSSSCG00000035544 | 2.96 | 1.42E-19 | 3.54 | 0.00166 |  |  |
| 53 | ENSSSCG00000008677 | ENSSSCG00000008677 | 2.84 | 1.71E-11 | 4.23 | 0.000552 |  |  |
| 54 | ENSSSCG00000010457 | KIF20B | 2.78 | 5.61E-15 | 3.83 | 0.000639 |  |  |
| 55 | ENSSSCG00000010461 | ANKRD1 | 2.77 | 3.00E-17 | -0.34 | 1 |  |  |
| 56 | ENSSSCG00000015924 | SPC25 | 2.76 | 4.63E-12 | 3.86 | 0.00181 |  |  |
| 57 | ENSSSCG00000009178 | H2AFZ | 2.72 | 2.76E-19 | 1.74 | 1 |  |  |
| 58 | ENSSSCG00000010923 | UBE2T | 2.71 | 1.34E-16 | 2.91 | 0.0342 |  |  |
| 59 | ENSSSCG00000032996 | SLC7A5 | 2.70 | 8.17E-16 | 1.21 | 1 |  |  |
| 60 | ENSSSCG00000005047 | CDKN3 | 2.66 | 8.73E-16 | 2.84 | 0.0483 |  |  |
| 61 | ENSSSCG00000009093 | BBS7 | 2.63 | 1.91E-15 | 3.02 | 0.022 |  |  |
| 62 | ENSSSCG00000033593 | CIP2A | 2.62 | 2.79E-12 | 3.20 | 0.0298 |  |  |
| 63 | ENSSSCG00000004518 | SKA1 | 2.59 | 8.69E-12 | 3.89 | 0.000639 |  |  |
| 64 | ENSSSCG00000010568 | NPM3 | 2.57 | 2.91E-15 | -0.13 | 1 |  |  |
| 65 | ENSSSCG00000016600 | TMEM229A | 2.51 | 2.13E-13 | 0.52 | 1 |  |  |
| 66 | ENSSSCG00000005983 | ATAD2 | 2.48 | 2.37E-12 | 3.45 | 0.00311 |  |  |
| 67 | ENSSSCG00000015785 | CENPU | 2.48 | 2.50E-11 | 3.31 | 0.012 |  |  |
| 68 | ENSSSCG00000011538 | LMCD1 | 2.47 | 6.99E-11 | -1.30 | 1 |  |  |
| 69 | ENSSSCG00000008729 | LYAR | 2.35 | 1.45E-11 | 2.66 | 0.123 |  |  |
| 70 | ENSSSCG00000032094 | DKK2 | 2.34 | 3.81E-10 | -1.36 | 1 |  |  |
| 71 | ENSSSCG00000007043 | GPCPD1 | 2.33 | 1.48E-11 | -0.49 | 1 |  |  |
| 72 | ENSSSCG00000034522 | TRIP13 | 2.32 | 1.81E-10 | 2.94 | 0.0483 |  |  |
| 73 | ENSSSCG00000008289 | MTHFD2 | 2.26 | 5.35E-12 | 0.59 | 1 |  |  |
| 74 | ENSSSCG00000033444 | SPC24 | 2.25 | 1.12E-11 | 2.72 | 0.0509 |  |  |
| 75 | ENSSSCG00000037016 | ID1 | 2.24 | 7.01E-11 | -0.60 | 1 |  |  |
| 76 | ENSSSCG00000035746 | ECT2 | 2.21 | 2.03E-10 | 2.88 | 0.0368 |  |  |
| 77 | ENSSSCG00000003789 | CTH | 2.21 | 1.76E-10 | 1.10 | 1 |  |  |
| 78 | ENSSSCG00000003583 | RPA2 | 2.19 | 2.49E-10 | 1.61 | 1 |  |  |
| 79 | ENSSSCG00000011731 | SMC4 | 2.17 | 9.36E-11 | 2.95 | 0.0172 |  |  |
| 80 | ENSSSCG00000035908 | ENSSSCG00000035908 | 2.13 | 5.47E-10 | 2.25 | 0.41 |  |  |
| 81 | ENSSSCG00000032180 | MCM7 | 2.12 | 2.45E-08 | 2.33 | 0.552 |  |  |
| 82 | ENSSSCG00000009704 | HMGB2 | 2.11 | 4.52E-10 | 3.26 | 0.00253 |  |  |
| 83 | ENSSSCG00000004139 | ADGRG6 | 2.11 | 1.50E-10 | 0.72 | 1 |  |  |
| 84 | ENSSSCG00000006051 | CTHRC1 | 2.04 | 4.15E-09 | -1.34 | 1 |  |  |
| 85 | ENSSSCG00000021966 | TOPBP1 | 2.03 | 6.98E-09 | 1.71 | 1 |  |  |
| 86 | ENSSSCG00000006153 | FABP5 | 2.00 | 9.37E-08 | -1.67 | 1 |  |  |
| **Downregulated** | | | | | | |  |  |
| **SI/No** | **Feature ID** | **Genes** | **C FC** | **P-Value** | **ISC/R FC** | **P-Value** |  |  |
| 1 | ENSSSCG00000026044 | FDFT1 | -2.07 | 5.47E-04 | -0.03 | 1 |  |  |
| 2 | ENSSSCG00000016261 | SP110 | -2.07 | 7.17E-04 | 0.14 | 1 |  |  |
| 3 | ENSSSCG00000004241 | GJA1 | -2.08 | 6.58E-04 | -2.13 | 1 |  |  |
| 4 | ENSSSCG00000011928 | CCDC80 | -2.13 | 6.15E-05 | -0.93 | 1 |  |  |
| 5 | ENSSSCG00000000033 | TSPO | -2.14 | 5.46E-05 | 0.01 | 1 |  |  |
| 6 | ENSSSCG00000035249 | GADD45G | -2.15 | 3.21E-04 | 0.11 | 1 |  |  |
| 7 | ENSSSCG00000008496 | EIF2AK2 | -2.16 | 1.69E-04 | 0.02 | 1 |  |  |
| 8 | ENSSSCG00000009240 | ENSSSCG00000009240 | -2.16 | 3.09E-04 | -0.04 | 1 |  |  |
| 9 | ENSSSCG00000008957 | AMCF-II | -2.16 | 1.36E-02 | 0.24 | 1 |  |  |
| 10 | ENSSSCG00000008959 | CXCL2 | -2.19 | 1.03E-03 | 0.32 | 1 |  |  |
| 11 | ENSSSCG00000038918 | CTSF | -2.20 | 1.49E-04 | -0.39 | 1 |  |  |
| 12 | ENSSSCG00000017614 | TRIM25 | -2.21 | 2.51E-04 | 0.48 | 1 |  |  |
| 13 | ENSSSCG00000008857 | MSMO1 | -2.23 | 1.05E-04 | -0.44 | 1 |  |  |
| 14 | ENSSSCG00000040184 | LMO7 | -2.26 | 2.18E-04 | 0.24 | 1 |  |  |
| 15 | ENSSSCG00000000916 | LUM | -2.28 | 5.29E-05 | -1.04 | 1 |  |  |
| 16 | ENSSSCG00000015301 | STEAP1 | -2.30 | 5.99E-05 | -0.01 | 1 |  |  |
| 17 | ENSSSCG00000005981 | FBXO32 | -2.33 | 7.41E-05 | -1.28 | 1 |  |  |
| 18 | ENSSSCG00000020705 | MAP3K8 | -2.35 | 6.42E-05 | -0.27 | 1 |  |  |
| 19 | ENSSSCG00000001912 | PML | -2.36 | 7.42E-05 | 0.19 | 1 |  |  |
| 20 | ENSSSCG00000004651 | GALK2 | -2.42 | 5.63E-05 | -1.11 | 1 |  |  |
| 21 | ENSSSCG00000001952 | NFKBIA | -2.43 | 5.76E-05 | 0.06 | 1 |  |  |
| 22 | ENSSSCG00000027157 | SLC40A1 | -2.46 | 2.03E-05 | -1.04 | 1 |  |  |
| 23 | ENSSSCG00000036213 | FGF2 | -2.50 | 4.05E-05 | -1.50 | 1 |  |  |
| 24 | ENSSSCG00000015595 | ATF3 | -2.53 | 4.78E-05 | -0.49 | 1 |  |  |
| 25 | ENSSSCG00000029066 | IDI1 | -2.57 | 9.27E-06 | -0.84 | 1 |  |  |
| 26 | ENSSSCG00000030484 | AHR | -2.63 | 4.28E-06 | -0.29 | 1 |  |  |
| 27 | ENSSSCG00000004049 | ACAT2 | -2.65 | 6.80E-06 | 0.01 | 1 |  |  |
| 28 | ENSSSCG00000016322 | ACKR3 | -2.67 | 8.36E-06 | -0.03 | 1 |  |  |
| 29 | ENSSSCG00000036383 | LGALS3BP | -2.68 | 1.21E-05 | 0.04 | 1 |  |  |
| 30 | ENSSSCG00000016872 | HMGCS1 | -2.69 | 6.09E-06 | -0.91 | 1 |  |  |
| 31 | ENSSSCG00000024388 | BNIP3 | -2.70 | 1.19E-06 | -0.42 | 1 |  |  |
| 32 | ENSSSCG00000038912 | IFITM3 | -2.70 | 1.34E-06 | 0.00 | 1 |  |  |
| 33 | ENSSSCG00000040317 | SOD2 | -2.75 | 2.64E-06 | -0.31 | 1 |  |  |
| 34 | ENSSSCG00000001910 | ISLR | -2.75 | 3.86E-06 | -0.22 | 1 |  |  |
| 35 | ENSSSCG00000015311 | CYP51A1 | -2.78 | 2.31E-06 | 0.05 | 1 |  |  |
| 36 | ENSSSCG00000023379 | UBE2L6 | -2.78 | 2.50E-06 | -0.06 | 1 |  |  |
| 37 | ENSSSCG00000037572 | EPSTI1 | -2.80 | 3.25E-05 | 1.05 | 1 |  |  |
| 38 | ENSSSCG00000031356 | HES1 | -2.85 | 9.25E-07 | -0.37 | 1 |  |  |
| 39 | ENSSSCG00000024018 | SLC16A3 | -2.88 | 6.32E-07 | -0.20 | 1 |  |  |
| 40 | ENSSSCG00000036136 | BHLHE40 | -2.95 | 6.15E-07 | -1.05 | 1 |  |  |
| 41 | ENSSSCG00000015897 | IFIH1 | -2.98 | 1.96E-06 | 1.02 | 1 |  |  |
| 42 | ENSSSCG00000015798 | ANKRD37 | -2.99 | 1.48E-06 | 0.49 | 1 |  |  |
| 43 | ENSSSCG00000027660 | IFI44L | -3.08 | 1.42E-07 | -0.16 | 1 |  |  |
| 44 | ENSSSCG00000032436 | ENSSSCG00000032436 | -3.23 | 2.00E-08 | 0.00 | 1 |  |  |
| 45 | ENSSSCG00000025822 | SFRP1 | -3.25 | 3.75E-08 | -1.23 | 1 |  |  |
| 46 | ENSSSCG00000006002 | CCN3 | -3.26 | 7.65E-06 | -1.12 | 1 |  |  |
| 47 | ENSSSCG00000030548 | HERC5 | -3.28 | 6.06E-07 | 0.61 | 1 |  |  |
| 48 | ENSSSCG00000031262 | TXNIP | -3.29 | 2.06E-08 | -0.61 | 1 |  |  |
| 49 | ENSSSCG00000006512 | FDPS | -3.34 | 4.10E-08 | -0.44 | 1 |  |  |
| 50 | ENSSSCG00000017886 | FBXO39 | -3.36 | 4.57E-08 | 0.15 | 1 |  |  |
| 51 | ENSSSCG00000037697 | MGP | -3.40 | 1.22E-07 | 0.29 | 1 |  |  |
| 52 | ENSSSCG00000035392 | IGFBP2 | -3.64 | 1.39E-10 | -0.09 | 1 |  |  |
| 53 | ENSSSCG00000033453 | BST2 | -3.66 | 6.98E-09 | 0.06 | 1 |  |  |
| 54 | ENSSSCG00000014336 | EGR1 | -3.72 | 1.03E-10 | 0.02 | 1 |  |  |
| 55 | ENSSSCG00000002383 | FOS | -3.76 | 1.79E-09 | 0.49 | 1 |  |  |
| 56 | ENSSSCG00000009881 | OAS2 | -3.82 | 6.23E-10 | 0.13 | 1 |  |  |
| 57 | ENSSSCG00000021712 | HERC6 | -3.87 | 5.98E-09 | 0.57 | 1 |  |  |
| 58 | ENSSSCG00000034570 | ENSSSCG00000034570 | -4.47 | 1.16E-13 | 0.04 | 1 |  |  |
| 59 | ENSSSCG00000031538 | RNASE4 | -4.55 | 7.50E-13 | -1.02 | 1 |  |  |
| 60 | ENSSSCG00000012077 | MX1 | -4.55 | 2.01E-13 | 0.15 | 1 |  |  |
| 61 | ENSSSCG00000016718 | NPY | -4.78 | 4.57E-08 | -1.62 | 1 |  |  |
| 62 | ENSSSCG00000035297 | ISG12(A) | -4.80 | 3.16E-15 | -0.20 | 1 |  |  |
| 63 | ENSSSCG00000008647 | CMPK2 | -4.82 | 5.66E-11 | 0.90 | 1 |  |  |
| 64 | ENSSSCG00000012076 | MX2 | -5.29 | 6.36E-13 | 0.46 | 1 |  |  |
| 65 | ENSSSCG00000040575 | ISG15 | -5.94 | 2.15E-17 | -0.22 | 1 |  |  |
| 66 | ENSSSCG00000010452 | IFIT1 | -6.35 | 1.43E-18 | -0.38 | 1 |  |  |
| 67 | ENSSSCG00000037358 | HPS5 | -6.64 | 2.70E-14 | -1.66 | 1 |  |  |
| **EATDS RPL30** | | | | | | | | |
| **Cluster 1** | | | | | | | | |
| **Upregulated** | | | | | | | | |
| **SI/No** | **Feature ID** | **Genes** | **C FC** | **P-Value** | **ISC FC** | **P-Value** | **ISC/R FC** | **P-Value** |
| 1 | ENSSSCG00000017705 | CCL5 | 3.28 | 4.46E-01 | -3.35 | 1.00E+00 | -0.32 | 1.00E+00 |
| 2 | ENSSSCG00000010452 | IFIT1 | 2.64 | 5.15E-07 | -1.10 | 8.14E-01 | -0.88 | 1.00E+00 |
| 3 | ENSSSCG00000037358 | HPS5 | 2.46 | 2.02E-01 | -0.32 | 1.00E+00 | -1.21 | 1.00E+00 |
| 4 | ENSSSCG00000040575 | ISG15 | 2.02 | 4.65E-06 | -1.86 | 7.15E-02 | 0.09 | 1.00E+00 |
| **Cluster 4** | | | | | | | | |
| **Downregulated** | | | | | | | | |
| **SI/No** | **Feature ID** | **Genes** | **C FC** | **P-Value** | **ISC FC** | **P-Value** | **ISC/R FC** | **P-Value** |
| 1 | ENSSSCG00000028996 | ALDH1A1 | -0.40 | 1.00E+00 | 0.62 | 7.22E-01 | -2.43 | 1.00E+00 |
| 2 | ENSSSCG00000003439 | DHRS3 | -0.45 | 1.00E+00 | -1.13 | 7.56E-01 | -2.88 | 1.00E+00 |
